# Correction: *Use of varenicline and nicotine replacement therapy in people with and without general practitioner-recorded dementia: retrospective cohort study of routine electronic medical records*

**DOI:** 10.1136/bmjopen-2018-027569corr1

**Published:** 2019-10-17

Itani T, Martin R, Rai D, *et al*. Use of varenicline and nicotine replacement therapy in people with and without general practitioner-recorded dementia: retrospective cohort study of routine electronic medical records. *BMJ Open* 2019;9:e027569. doi: 10.1136/bmjopen-2018-027569.

This article was previously published with an error.

Amy Taylor's MRC Funding (Grant Number: MC_UU_12013/6) is not acknowledged.

