# Supplementary material for: Use of varenicline and nicotine replacement therapy in people with and without general practitioner-recorded dementia: retrospective cohort study of routine electronic medical records
Source: BMJ Open. 2019 Aug 30;9(8):e027569. doi: 10.1136/bmjopen-2018-027569 (PMC6720236; doi:10.1136/bmjopen-2018-027569)
Supplement: Supplementary data [file bmjopen-2018-027569supp001.pdf]

eFigure 1. Flow chart of eligible study participants as per protocol restrictions

Assessed for eligibility
N Records=2,877,296
N Patients=566,936

Excluded from analysis due to protocol restrictions (Davies et al. 2015)

	Prescriptions	Patients
Prescriptions issued to patients under the age of 15	6580	1944
Prescription issued after patients' registration period ended	0	0
Prescription issued before patients' registration period began	296490	53529
Prescription issued to patient aged 16 or 17	13154	4425
Prescription issued before 1st September 2006	822244	145382
Both varenicline and NRT were prescribed on the same day	8289	264
Smoking cessation medication was not prescribed by a general practitioner (GP)	141810	16168
Smoking cessation medication prescription had less than one year of historical follow-up data prior to prescription	163821	41112
Bupropion prescriptions	32997	6981
Prescriptions issued to patients who received a smoking cessation medication issued in the previous 18 months	1069188	30278
Patient had previously received an eligible smoking cessation medication prescription	55870	0
Prescribing GP had seen less than 10 patients.	18494	18494
Patients had less than 180 days of follow-up after 1st prescription.	13045	13045
Total excluded N=	2,641,982	331,622

Included in analysis
N patients=235,314

People with dementia
N patients=447

People without dementia
N patients=234,867

Itani T, *et al. BMJ Open* 2019; 9:e027569. doi: 10.1136/bmjopen-2018-027569

eFigure 2. Age and sex standardised percentage (%) of primary care patients with an electronic medical record indicating smoking, from 2007 to 2015, in people with or without dementia

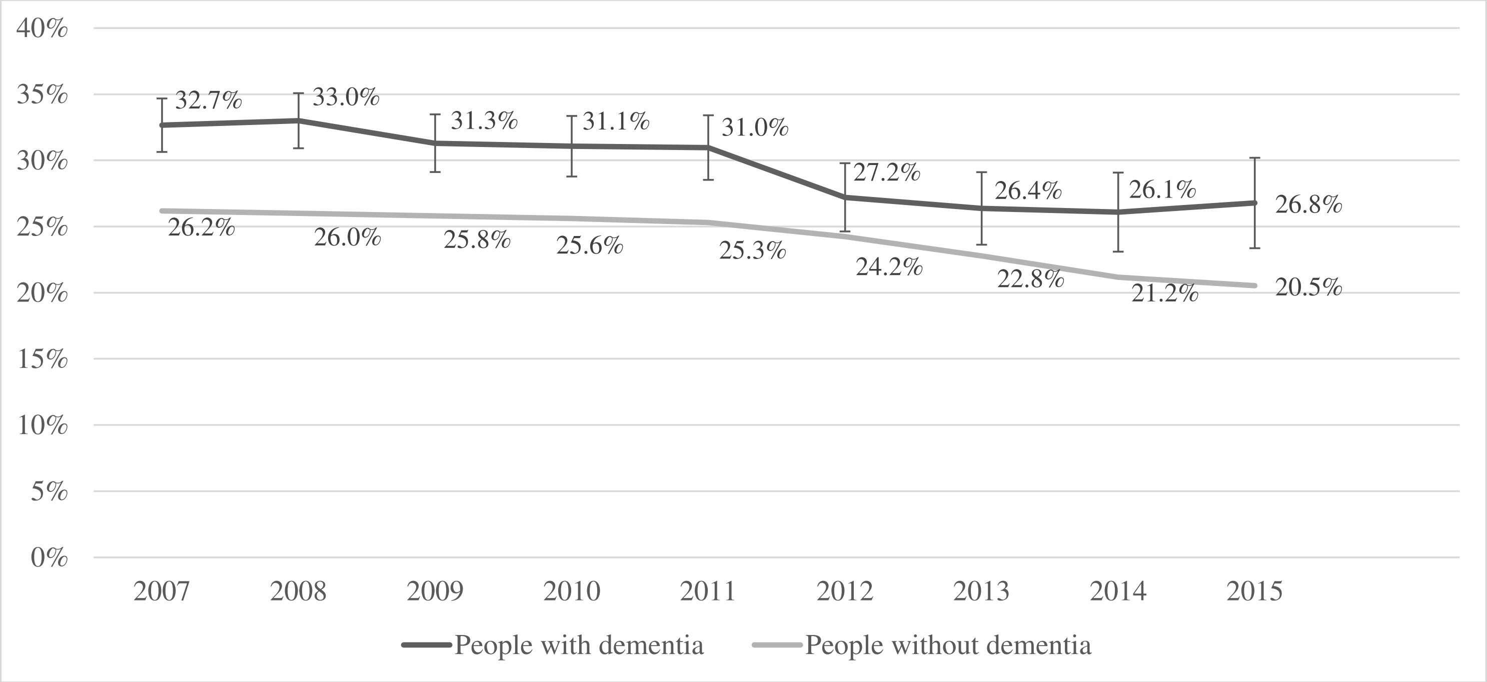

Note on age and sex standardisation

Smoking prevalence rates were directly age- and sex- standardised to account for differences in age and sex between groups with and without dementia. We used the CPRD population in 2015 as our standard population for calculating standardised smoking rates. Age was grouped into three categories (18-49, 50-59, 60+ years).

Itani T, *et al. BMJ Open* 2019; 9:e027569. doi: 10.1136/bmjopen-2018-027569

eTable 1. List of dementia medications codes used in study cohort

Prodcode	Product name
39363	Ebixa 20mg tablets (Lundbeck Ltd)
58937	Exelon 13.3mg/24hours transdermal patches (Novartis Pharmaceuticals UK Ltd)
6225	Memantine 10mg tablets
11751	Rivastigmine 3mg capsules
7329	Galantamine 20mg/5ml oral solution sugar free
60723	Rivastigmine 6mg capsules (Waymade Healthcare Plc)
58780	Voleze 9.5mg/24hours transdermal patches (Focus Pharmaceuticals Ltd)
39362	Ebixa tablets treatment initiation pack (Lundbeck Ltd)
56631	Rivastigmine 13.3mg/24hours transdermal patches
37132	Rivastigmine 9.5mg/24hours transdermal patches
56771	Rivastigmine 3mg capsules (Dr Reddy's Laboratories (UK) Ltd)
20404	Exelon 4.5mg capsules (Novartis Pharmaceuticals UK Ltd)
57171	Erastig 9.5mg/24hours transdermal patches (Teva UK Ltd)
61676	Donepezil 1mg/ml oral solution sugar free
24088	Reminyl XL 24mg capsules (Shire Pharmaceuticals Ltd)
11635	Galantamine 12mg tablets
60192	Galantex XL 8mg capsules (Creo Pharma Ltd)
57627	Erastig 4.6mg/24hours transdermal patches (Teva UK Ltd)
11654	Galantamine 8mg tablets
2930	Donepezil 5mg tablets
5616	Exelon 6mg capsules (Novartis Pharmaceuticals UK Ltd)
58969	Rivastigmine 4.6mg/24hours transdermal patches (A A H Pharmaceuticals Ltd)
48482	Galsya XL 8mg capsules (Consilient Health Ltd)
48442	Donepezil 5mg orodispersible tablets
55928	Exelon 4.5mg capsules (Waymade Healthcare Plc)
53882	Rivastigmine 2mg/ml oral solution
58709	Donepezil 10mg tablets (A A H Pharmaceuticals Ltd)
59871	Donepezil 10mg/5ml oral suspension

55720	Gatalin XL 24mg capsules (Aspire Pharma Ltd)
7361	Galantamine 24mg modified-release capsules
61476	Acumor XL 24mg capsules (Generics (UK) Ltd)
57139	Ebixa 10mg tablets (DE Pharmaceuticals)
62164	Alzest 9.5mg/24hours transdermal patches (Dr Reddy's Laboratories (UK) Ltd)
53922	Donepezil 10mg orodispersible tablets (Consilient Health Ltd)
36848	Aricept Evess 5mg orodispersible tablets (Eisai Ltd)
60493	Galantex XL 24mg capsules (Creo Pharma Ltd)
29288	Reminyl 4mg/ml oral solution (Shire Pharmaceuticals Ltd)
9966	Ebixa 5mg/pump actuation oral solution (Lundbeck Ltd)
58947	Donepezil 10mg tablets (Accord Healthcare Ltd)
5247	Aricept 10mg tablets (Eisai Ltd)
11716	Exelon 3mg capsules (Novartis Pharmaceuticals UK Ltd)
61920	Luventa XL 8mg capsules (Fontus Health Ltd)
10187	Galantamine 4mg tablets
37444	Exelon 4.6mg/24hours transdermal patches (Novartis Pharmaceuticals UK Ltd)
18587	Reminyl XL 8mg capsules (Shire Pharmaceuticals Ltd)
56421	Gatalin XL 8mg capsules (Aspire Pharma Ltd)
10255	Galantamine 8mg modified-release capsules
4597	Rivastigmine 1.5mg capsules
9854	Reminyl 4mg tablets (Shire Pharmaceuticals Ltd)
61385	Nemdatine 10mg tablets (Actavis UK Ltd)
11546	Exelon 1.5mg capsules (Novartis Pharmaceuticals UK Ltd)
14309	Galantamine 16mg modified-release capsules
37188	Aricept Evess 10mg orodispersible tablets (Eisai Ltd)
5334	Reminyl 12mg tablets (Shire Pharmaceuticals Ltd)
56709	Gatalin XL 16mg capsules (Aspire Pharma Ltd)
2931	Donepezil 10mg tablets
61921	Luventa XL 24mg capsules (Fontus Health Ltd)
11827	Rivastigmine 2mg/ml oral solution sugar free
37957	Exelon 9.5mg/24hours transdermal patches (Novartis Pharmaceuticals UK Ltd)

Itani T, *et al. BMJ Open* 2019; 9:e027569. doi: 10.1136/bmjopen-2018-027569

48015	Galsya XL 24mg capsules (Consilient Health Ltd)
53842	Aricept 5mg tablets (Waymade Healthcare Plc)
18800	Ebixa 10mg tablets (Lundbeck Ltd)
59993	Galantex XL 16mg capsules (Creo Pharma Ltd)
36976	Rivastigmine 4.6mg/24hours transdermal patches
56600	Donepezil 5mg tablets (Zentiva)
35088	Donepezil 10mg orodispersible tablets sugar free
5400	Aricept 5mg tablets (Eisai Ltd)
59330	Voleze 4.6mg/24hours transdermal patches (Focus Pharmaceuticals Ltd)
18062	Reminyl 8mg tablets (Shire Pharmaceuticals Ltd)
11752	Rivastigmine 4.5mg capsules
38976	Memantine 5mg+10mg+15mg+20mg Tablet
35179	Donepezil 5mg orodispersible tablets sugar free
60107	Donepezil 5mg tablets (Alliance Healthcare (Distribution) Ltd)
20140	Reminyl XL 16mg capsules (Shire Pharmaceuticals Ltd)
39240	Memantine 20mg tablets
18556	Exelon 2mg/ml oral solution (Novartis Pharmaceuticals UK Ltd)
9786	Rivastigmine 6mg capsules
11837	Memantine 10mg/ml oral solution sugar free
48443	Donepezil 10mg orodispersible tablets
61618	Nemdatine 20mg tablets (Actavis UK Ltd)
12843	Ginkyo 120mg tablets (Ceuta Healthcare Ltd)
30120	Ginkyo 50mg tablets (Ceuta Healthcare Ltd)
61128	HealthAid Ginko Vital (Biloba) 5g capsules (HealthAid Ltd)

Itani T, *et al. BMJ Open* 2019; 9:e027569. doi: 10.1136/bmjopen-2018-027569

eTable 2. List of dementia diagnoses codes used in study cohort

medcode	readcode	readterm
26270	Eu02500	[X]Lewy body dementia
44674	E002.00	Senile dementia with depressive or paranoid features
19393	Eu01z00	[X]Vascular dementia, unspecified
4693	Eu02z00	[X] Unspecified dementia
25704	Eu00011	[X]Presenile dementia,Alzheimer's type
19477	E004.00	Arteriosclerotic dementia
55313	Eu01y00	[X]Other vascular dementia
30032	E001200	Presenile dementia with paranoia
18386	E002000	Senile dementia with paranoia
56912	E004100	Arteriosclerotic dementia with delirium
33707	E00..00	Senile and presenile organic psychotic conditions
4357	Eu02z14	[X] Senile dementia NOS
12710	6AB..00	Dementia annual review
41089	E002z00	Senile dementia with depressive or paranoid features NOS
9509	Eu02300	[X]Dementia in Parkinson's disease
15165	E001.00	Presenile dementia
49513	E001100	Presenile dementia with delirium
42602	E001000	Uncomplicated presenile dementia
7572	F116.00	Lewy body disease
30706	Eu00200	[X]Dementia in Alzheimer's dis, atypical or mixed type
1916	E00..11	Senile dementia
55467	E004200	Arteriosclerotic dementia with paranoia
9565	Eu01.11	[X]Arteriosclerotic dementia
8934	Eu01200	[X]Subcortical vascular dementia
31016	Eu01300	[X]Mixed cortical and subcortical vascular dementia
43089	E004000	Uncomplicated arteriosclerotic dementia
11175	Eu01100	[X]Multi-infarct dementia
42279	E004z00	Arteriosclerotic dementia NOS
29386	Eu00z00	[X]Dementia in Alzheimer's disease, unspecified
38438	E001z00	Presenile dementia NOS
8634	E004.11	Multi infarct dementia
1917	F110.00	Alzheimer's disease
61528	Eu00013	[X]Alzheimer's disease type 2
1350	E00..12	Senile/presenile dementia
38678	Eu00100	[X]Dementia in Alzheimer's disease with late onset
27677	E001300	Presenile dementia with depression
46762	Eu00111	[X]Alzheimer's disease type 1
11379	Eu00112	[X]Senile dementia,Alzheimer's type
21887	E002100	Senile dementia with depression
2882	E00z.00	Senile or presenile psychoses NOS
25386	E041.00	Dementia in conditions EC
49263	Eu00000	[X]Dementia in Alzheimer's disease with early onset
59122	Fyu3000	[X]Other Alzheimer's disease
34944	Eu02z13	[X] Primary degenerative dementia NOS

Itani T, *et al. BMJ Open* 2019; 9:e027569. doi: 10.1136/bmjopen-2018-027569

40805	9hD1.00	Excepted from dementia quality indicators: Informed dissent
37015	E003.00	Senile dementia with delirium
6578	Eu01.00	[X]Vascular dementia
7323	E000.00	Uncomplicated senile dementia
55838	Eu01111	[X]Predominantly cortical dementia
29512	F112.00	Senile degeneration of brain
12621	Eu02.00	[X]Dementia in other diseases classified elsewhere
7664	Eu00.00	[X]Dementia in Alzheimer's disease
60059	Eu00012	[X]Primary degen dementia, Alzheimer's type, presenile onset
15249	E00y.00	Other senile and presenile organic psychoses
43346	Eu00113	[X]Primary degen dementia of Alzheimer's type, senile onset
53446	Eu04100	[X]Delirium superimposed on dementia
43292	E004300	Arteriosclerotic dementia with depression
32057	F110100	Alzheimer's disease with late onset
8195	Eu00z11	[X]Alzheimer's dementia unspec
64267	Eu02y00	[X]Dementia in other specified diseases classif elsewhere
46488	Eu01000	[X]Vascular dementia of acute onset
27759	Eu02z16	[X] Senile dementia, depressed or paranoid type
16797	F110000	Alzheimer's disease with early onset
48501	Eu02z11	[X] Presenile dementia NOS
109047	8BPa.00	Antipsychotic drug therapy for dementia
106311	8CMZ.00	Dementia care plan
103445	8Hla.00	Referral to dementia care advisor
44341	9hD..00	Exception reporting: dementia quality indicators
28402	Eu02000	[X]Dementia in Pick's disease
54106	Eu02100	[X]Dementia in Creutzfeldt-Jakob disease
37014	Eu02200	[X]Dementia in Huntington's disease
41185	Eu02400	[X]Dementia in human immunodef virus [HIV] disease
5931	1461	H/O: dementia
11136	F111.00	Pick's disease
62132	E02y100	Drug-induced dementia
2731	F11z.11	Cerebral atrophy
54744	F11x200	Cerebral degeneration due to cerebrovascular disease

Itani T, *et al. BMJ Open* 2019; 9:e027569. doi: 10.1136/bmjopen-2018-027569

eTable 3. Distributions of imputed characteristics in the imputation datasets

Characteristic	People with dementia (N=447)							People without dementia (N=234,867)						
	NRT (N=409)		Varenicline (N=38)		Total		% of data imputed	NRT (N=159,327)		Varenicline (N=75,540)		Total		% of data imputed
	Mean	Standard error	Mean	Standard error	Mean	Standard error		Mean	Standard error	Mean	Standard error	Mean	Standard error	
Body mass index	24.6	0.28	26.6	1.02	24.7	0.27	10.3	26.4	0.02	26.5	0.02	26.4	0.01	14.1
Index of multiple deprivation	3.3	0.08	3.3	0.3	3.3	0.09	53.2	3.3	0.004	3.2	0.007	3.3	0.003	43.6

Itani T, *et al. BMJ Open* 2019; 9:e027569. doi: 10.1136/bmjopen-2018-027569

eTable 4. Raw numbers for smoking prevalence calculations amongst people with and without dementia, 2007-2015

**Year**	2007	2008	2009	2010	2011	2012	2013	2014	2015
People with dementia									
Numerator (number of smokers with dementia)	2,965	3,126	3,326	3,495	3,678	3,513	3,335	3,033	2,690
Denominator (number people with dementia)	27,432	29664	32,090	34,391	36,284	38,251	40,043	39,665	36,249
People without dementia									
Numerator (number of smokers without dementia)	1,010,530	1,013,985	1,018,065	1,015,538	987,193	930,783	866,310	748,084	628,444
Denominator (number of people without dementia)	3,860,169	3,895,542	3,936,638	3,955,107	3,892,231	3,830,563	3,795,481	3,536,957	3,068,743

Itani T, *et al. BMJ Open* 2019; 9:e027569. doi: 10.1136/bmjopen-2018-027569

eTable 5. The likelihood of smokers with ever dementia diagnosis being prescribed varenicline versus NRT, as compared to smokers with no ever dementia diagnosis, N=235,314

	**Partially adjusted odds ratio (95% confidence interval) †**	**Fully adjusted odds ratio (95% confidence interval) ††**
Ever dementia diagnosis	0.21 (0.15 to 0.29)	0.26 (0.18 to 0.36)

‡Partially adjusted models were adjusted for: age, sex, year of first prescription.
†† Fully adjusted models were adjusted for: age, sex, days in history, IMD, number of GP visits 1-year prior to first prescription, BMI, year of first prescription, history of major physical morbidity (Charlson Index), alcohol misuse ever, drug misuse ever, depression ever, neurotic disorder ever, self-harm ever, antidepressant prescription ever, antipsychotic prescription ever, hypnotics/anxiolytics prescription ever, other psychotropic medication ever, and other behavioral/neurologic disorder ever. Missing BMI and IMD values were imputed using multiple imputation.
Models were estimated using cluster robust standard errors to account for potential clustering of patients between practices.

Itani T, *et al. BMJ Open* 2019; 9:e027569. doi: 10.1136/bmjopen-2018-027569

eTable 6. Number and percentage (%) of people with an electronic medical record indicating smoking cessation at 3, 6 and 9-months, and 1, 2, and 4-years follow-up, in people with and without dementia

	**3-months**	**6-months**	**9-months**	**1-year**	**2-years**	**4-years**
People without dementia N=234867	36223 (15.4%)	45796 (19.5%)	50193 (21.4%)	53263 (22.7%)	60257 (25.7%)	69842 (29.7%)
People with dementia N=447	51 (11.4%)	77 (17.2%)	92 (20.6%)	102 (22.8%)	137 (30.6%)	155 (34.7%)

Itani T, *et al. BMJ Open* 2019; 9:e027569. doi: 10.1136/bmjopen-2018-027569

eTable 7. Fully adjusted odds ratios and 95% confidence intervals for the association between the diagnosis of dementia and smoking cessation at 3, 6 and 9-months and 1, 2, and 4-years after prescription

**Fully adjusted odds ratio (95% confidence interval) †**						
	3-months	6-months	9-months	1-year	2-years	4-years
(N=235,314)	0.56 (0.41 to 0.75)	0.64 (0.49 to 0.83)	0.71 (0.56 to 0.90)	0.75 (0.60 to 0.94)	1.0 (0.81 to 1.23)	1.04 (0.85 to 1.26)

† Fully adjusted models were adjusted for: type of prescription, age, sex, days in history, IMD, number of GP visits 1-year prior to first prescription, BMI, year of first prescription, history of major physical morbidity (Charlson Index), alcohol misuse ever, drug misuse ever, depression ever, neurotic disorder ever, self-harm ever, antidepressant prescription ever, antipsychotic prescription ever, hypnotics/anxiolytics prescription ever, other psychotropic medication ever, and other behavioral/neurologic disorder ever. Missing BMI and IMD values were imputed using multiple imputation.

Itani T, *et al. BMJ Open* 2019; 9:e027569. doi: 10.1136/bmjopen-2018-027569
