# Supplementary material for: Use of varenicline and nicotine replacement therapy in people with and without general practitioner-recorded dementia: retrospective cohort study of routine electronic medical records
Source: BMJ Open. 2019 Aug 30;9(8):e027569. doi: 10.1136/bmjopen-2018-027569 (PMC6720236; doi:10.1136/bmjopen-2018-027569)
Supplement: Author's manuscript [file bmjopen-2018-027569.draft_revisions.pdf]

BMJ Open is committed to open peer review. As part of this commitment we make the peer review history of every article we publish publicly available.

When an article is published we post the peer reviewers' comments and the authors' responses online. We also post the versions of the paper that were used during peer review. These are the versions that the peer review comments apply to.

The versions of the paper that follow are the versions that were submitted during the peer review process. They are not the versions of record or the final published versions. They should not be cited or distributed as the published version of this manuscript.

BMJ Open is an open access journal and the full, final, typeset and author-corrected version of record of the manuscript is available on our site with no access controls, subscription charges or pay-per-view fees (http://bmjopen.bmj.com).

If you have any questions on BMJ Open's open peer review process please email info.bmjopen@bmj.com

BMJ Open

Prescribing rates and long-term effectiveness of smoking cessation agents in people with and without dementia: prospective cohort study of electronic medical records.

Journal:	*BMJ Open*
Manuscript ID	bmjopen-2018-027569
Article Type:	Research
Date Submitted by the Author:	29-Oct-2018
Complete List of Authors:	Itani, Taha; School of Psychological Science,<br>Martin, Richard; University of Bristol, Social Medicine<br>Rai, Dheeraj; University of Bristol, Centre for Academic Mental Health, School of Social & Community Medicine<br>Jones, Tim; NIHR CLAHRC West,<br>Taylor, Gemma; University of Birmingham, Department of Primary Care Clinical Sciences<br>Thomas, Kyla; University of Bristol, School of Social and Community Medicine<br>Munafo, Marcus; University of Bristol, Experimental Psychology<br>Davies, Neil; University of Bristol,<br>Taylor, Amy; 5. School of Experimental Psychology University of Bristol,
Keywords:	Dementia < NEUROLOGY, Smoking cessation, Smoking prevalence

SCHOLARONE™
Manuscripts

PAPER TITLE

Prescribing rates and long-term effectiveness of smoking cessation agents in people with and without dementia: prospective cohort study of electronic medical records.

Authors

Taha Itani PhD[1,2]; Richard M Martin PhD [1,3,4]; Dheeraj Rai PhD[3,4,5], Tim Jones PhD[6]; Gemma MJ Taylor PhD[7] Kyla H Thomas PhD[4]; Marcus R Munafò PhD[1,2]; Neil M Davies PhD[1,4] and Amy E Taylor PhD[3,4]

Author affiliations

1 Medical Research Council Integrative Epidemiology Unit at the University of Bristol, BS8 2BN, United Kingdom.

2 UK Centre for Tobacco and Alcohol Studies, School of Psychological Science, University of Bristol, 12a Priory Road, Bristol, BS8 1TU, United Kingdom.

3 NIHR Biomedical Research Centre at the University Hospitals Bristol NHS Foundation Trust and the University of Bristol, United Kingdom.

4 Population Health Sciences, Bristol Medical School, University of Bristol, Oakfield Grove, Bristol, BS8 2BN, United Kingdom.5 Centre for Academic Mental Health; Bristol Medical School, University of Bristol, Barley House, Oakfield Grove, Bristol, BS8 2BN, United Kingdom.

5 Avon & Wiltshire Partnership NHS Mental Health Trust, Bristol, UK

6 National Institute for Health Research Collaboration for Leadership in Applied Health Research and Care West (NIHR CLAHRC West) at University Hospitals Bristol NHS Foundation Trust, 9th Floor Whitefriars, Lewins Mead, Bristol, BS1 2NT, United Kingdom.

7 Department of Psychology, University of Bath, Claverton Down, Bath, BA2 7AY.

For peer review only - http://bmjopen.bmj.com/site/about/guidelines.xhtml

Author Positions

Taha Itani, Research Associate in Epidemiology; Richard M Martin, Professor of Clinical Epidemiology; Dheeraj Rai, Consultant Senior Lecturer in Psychiatry; Tim Jones, Senior Research Associate; Gemma MJ Taylor, Lecturer in Clinical Psychology; Kyla H Thomas, Consultant Senior Lecturer in Public Health Medicine and NIHR Postdoctoral Fellow; Marcus R Munafò, Professor of Biological Psychology; Neil M Davies, Senior Research Fellow and Amy E Taylor, Research Fellow.

Correspondence

Dr Taha Itani, ti17926@bristol.ac.uk

Word count

2,666

review only

For peer review only - http://bmjopen.bmj.com/site/about/guidelines.xhtml

ABSTRACT

Objectives: Our primary objective was to determine the long-term effectiveness in terms of smoking quit rates of varenicline and nicotine replacement therapy (NRT) in people with dementia compared to those without the disease. Our secondary objectives were to estimate smoking prevalence and prescribing rates of varenicline and NRT in people with and without dementia.

Design: A prospective cohort study based on the analysis of electronic medical records within the Clinical Practice Research Datalink (2007-2015).

Setting: 683 general practices in England.

Participants: People with and without dementia, aged 18 years and have a code indicating that they are a current smoker.

Intervention: Prescription of varenicline or NRT.

Outcome measures: The primary effectiveness outcome was smoking cessation at 2-years, and the secondary effectiveness outcomes were smoking cessation at 3, 6, 9 months and 1, and 4 years.

Results: There were 235,314 people aged 18 years and above prescribed NRT or varenicline. Amongst smokers with dementia (N=447), 409 were prescribed NRT and 38 varenicline. Overall, we observed a steady increase in quit rates in people with and without dementia who were prescribed either varenicline or NRT throughout the study's follow-up period (3-months to 4-years). Smokers with dementia were 74% less likely (95% confidence interval: 64% to 82%) to be prescribed varenicline than NRT, compared to smokers without dementia. Smoking prevalence amongst people without dementia (N=628,116/3,062,917) was almost three times (21% vs. 7%) that in people with dementia (N=3,018/42,075) in 2015. Compared to people without dementia, people with dementia had consistently lower prescribing rates of varenicline from 2007 to 2015.

Conclusions: People with dementia are less likely to be prescribed varenicline despite some evidence of its long-term effectiveness. These findings highlight the need to provide more smoking cessation interventions for people with dementia.

For peer review only - http://bmjopen.bmj.com/site/about/guidelines.xhtml

Strengths and limitations of this study

- This study used primary care data from the Clinical Practice Research Datalink (CPRD) which are representative of the UK population.
- Expert-reviewed codelists were developed to define both the exposure and the outcome which would reduce misclassification bias.
- Due to the relatively small sample size of people with dementia, we were not able to test the relative effectiveness of varenicline versus NRT on smoking cessation using regression models.
- Data on smokers who purchase over-the-counter NRT were not available, and therefore the prevalence of NRT might be underestimated.

For peer review only

INTRODUCTION

Smoking is a leading cause of mortality and morbidity worldwide. About 12% of global deaths were attributed to smoking in 2015.[1] There is substantial evidence that smoking is associated with an increased risk of developing dementia.[2 3] For instance, it is estimated that 14% of Alzheimer's disease (AD) cases worldwide are attributable to smoking.[4] Smoking is thought to accelerate the onset of dementia mainly via vascular risk factors such as narrowing of blood vessels in the heart and the brain, thereby triggering oxidative stress.[4 5]

However, few studies report smoking prevalence among people with dementia. In a cross-sectional analysis of patients treated for AD in a neurology clinic during a 2-year period, past smoking prevalence was 29% (N=21/72).[6] In a case-control study of patients with vascular dementia, the rate of current tobacco use was 9% (N=17/198) as compared to 6% (N=11/199) in the control group.[7] Beyond the harmful health effects of smoking in people with dementia, there are concerns that smokers in this group may have a higher likelihood of fire accidents due to their compromised cognitive state.[8]

Since there are currently no available treatments to cure dementia, there is a growing interest in identifying modifiable risk factors for reducing the occurrence of the disease, to delay dementia onset, and reduce its burden.[4 9] Smoking cessation could potentially decrease the risk or slow the development of dementia[10] and could improve the quality of life of older adults through improved physical, and mental wellbeing.[9 11] Little is known about whether people with dementia are prescribed smoking cessation agents and whether they are effective in this group.

peer review only

For peer review only - http://bmjopen.bmj.com/site/about/guidelines.xhtml

Data from observational studies [12 13] and meta-analyses of randomized controlled trials[14] indicate that varenicline is more effective than nicotine replacement therapy (NRT) for smoking cessation in the general population. However, the effectiveness of varenicline for smoking cessation amongst people with dementia remains unknown.

Therefore in this study we aimed to: 1) estimate the relative effectiveness (quit rates) of varenicline and NRT on smoking cessation in people with dementia, compared to those without dementia, at 3, 6, 9 months and 1, 2, 4 years after first prescription; and 2) describe the rates of smoking prevalence and smoking cessation medication prescribing amongst people with and without dementia in UK primary care settings from 2007 to 2015.

For peer review only

For peer review only - http://bmjopen.bmj.com/site/about/guidelines.xhtml

METHODS

Data source and population

We conducted a prospective cohort study using electronic medical records from 683 general practices in England from 2007 to 2015 using data from the Clinical Practice Research Database (CPRD). Patient data from the CPRD are broadly representative of the UK general population in terms of age, sex and ethnicity.[15] These data have been validated, audited, and quality checked.[16] The study's protocol (15_115RMn2AMn) was approved by the Independent Scientific Advisory Committee for MHRA Database Research (https://www.cprd.com/isac/).

Code lists

We defined variables using medical and product codes within the CPRD. All code lists were developed using a list from a previously published study.[12] These codelists were derived from the the British National Formulary (BNF) and the International Classification of Diseases (ICD-10) and then agreed upon by field experts (DR, KHT). A previous systematic review that checked the validity of coding of various diagnoses in what was then the General Practice Research Database (now CPRD) suggests that coding for dementia and Alzheimer's is relatively accurate.[17]

Study subjects

During the study period (2007 to 2015), we included people (aged ≥18 years) with information about their smoking status (either smokers or non-smokers) for smoking prevalence estimates, and we included smokers prescribed either varenicline or NRT for prescribing prevalence and

For peer review only - http://bmjopen.bmj.com/site/about/guidelines.xhtml

effectiveness estimates. More detail about the inclusion and exclusion criteria are published in the study protocol.[18]

Patients were categorised as having dementia if: i) they had been diagnosed with dementia within 365-days prior to first varenicline/NRT prescription (based on ICD-10 diagnoses F00-F03), or ii) if they were prescribed dementia medications 365-days prior to smoking cessation medication prescription: (BNF chapter 4.11). Patients with no records of the above-mentioned diagnosis/prescriptions were considered to have no dementia.

Variables

Smoking and prescribing prevalence estimates

For smoking prevalence estimates, a patient's smoking status (aged ≥18 years) was defined by a record indicating smoking/non-smoking or prescription of NRT/varenicline in that year. In case of missing information about smoking, the patient's smoking status was carried forward until there was evidence of a change in smoking status and then carried backward. Records that were outside the registration period for each patient were excluded.

Prevalence of prescriptions of varenicline and NRT was calculated by dividing the number of prescriptions each year from 2007 to 2015 (there were very few varenicline prescriptions for patients with mental disorders in 2006) by the number of current smokers in each year. In both instances, prevalence was estimated for people with and without dementia. Individuals with missing smoking information were excluded from the denominators.

For peer review only - http://bmjopen.bmj.com/site/about/guidelines.xhtml

Exposure

Exposure was defined as prescription of varenicline or NRT (e.g., patches, etc.). Prescriptions used to define exposure groups were issued between September 1st, 2006 and August 31st, 2016, with no prior record of use of a related product in the preceding 18-months. We used the first treatment episode to ensure that intervention groups were "new users" of the medication.[19]

Outcome: smoking cessation

Smoking cessation was defined as having an electronic record indicating a non-smoking status. The closest smoking record to each follow-up period was selected to determine each study participant's smoking status; i.e., the most recent smoking record identified between cohort entry and each follow-up period (e.g., 3-months, 6-months). People with missing smoking data (beyond 180 days) were assumed to be continuing smokers[20] which has been previously found to be robust in sensitivity analyses.[12]

For peer review only

Covariates

Covariates included patients' age at time of prescription, sex, index of multiple deprivation score (IMD), mean number of GP visits one year prior to first prescription, year of first prescription of a smoking cessation medication, body mass index (BMI), days registered in the CPRD, the Charlson Index (a measure of chronic illness),[21] alcohol misuse, history of mental disorder or psychoactive medication prescriptions, evidence of other psychoactive medication prescription or other less common psychiatric disorders. We used multiple imputation to handle missing data on BMI and IMD. This was done using the ICE command in Stata where we produced 20 imputed datasets (eTable 1). We included all exposures, covariates, and outcomes in the imputation model.[22]

Follow-up

The primary effectiveness outcome was smoking cessation at 2-years, and the secondary effectiveness outcomes were smoking cessation at 3, 6, 9 months and 1, and 4 years after first prescription of varenicline or NRT.

Statistical analysis

Smoking prevalence

Smoking rates were calculated by dividing the number of people with dementia who had Read codes indicating current smoking for each year between 2007 and 2015 by the total number of people with a smoking status code (indicating current or non/ex-smoking) each year between

For peer review only - http://bmjopen.bmj.com/site/about/guidelines.xhtml

2007 and 2015. For comparison, smoking prevalence was also estimated amongst people without dementia.

Prescribing prevalence

The prevalence of varenicline and NRT prescribing amongst current smokers was calculated by dividing the number of prescriptions each year from 2007 to 2015 (there were no varenicline prescriptions for patients with dementia in 2006) by the number of current smokers in each year. Prevalence was estimated for people with and without dementia.

Effectiveness of varenicline and NRT

The effectiveness of varenicline and NRT on smoking cessation was determined by estimating quit rates at each follow-up period. This was calculated by dividing the number of non-smokers in each group by the total number of people in that group at each follow-up period. All analyses were conducted using Stata 14 MP.

For peer review only

RESULTS

Smoking prevalence and smoking cessation medication prescribing estimates

Smoking prevalence amongst people with dementia steadily decreased from 12% (N=10,121/84,647 in 2007 to 7% (N=3,018/42,075) in 2015. These estimates were consistently lower than in people without dementia, 26% (N= 1,003,374/ 3,802,954) in 2007 and 21% (N=628,116/3,062,917) in 2015 respectively. Smoking prevalence amongst people without dementia was almost three times that in people with dementia in 2015 (Figure 1).

The rate of NRT prescribing in people without dementia was 5% (51,367/1,007,563) in 2008 which decreased to 2% (N=13,607/628,116) by 2015, whereas little fluctuation was observed (remained at 5%) in NRT prescribing rates amongst people with dementia during the same period. Compared to people without dementia, people with dementia had lower prescribing rates of varenicline (Figure 2).

Effectiveness of varenicline and NRT for smoking cessation

The baseline characteristics of those eligible for the effectiveness analysis are presented in Table 1. Of the 235,314 people included in this analysis, 447 were people with dementia, whereas 234,867 were people without (eFigure 1). Overall, 159,736 smokers were prescribed NRT and 75,578 prescribed varenicline. The mean age of people with dementia at the time of smoking medication prescription was about 72 years (SD=12.6). People with dementia were about 25 years older, had more number GP visits 1-year prior to the first prescription, suffered from more comorbidities, and received more mental health-related prescriptions than those without dementia (Table 1).

For peer review only - http://bmjopen.bmj.com/site/about/guidelines.xhtml

Smokers with dementia were 74% (95% confidence interval: 64% to 82%) less likely to be prescribed varenicline than NRT, compared to smokers without dementia (eTable 2). We observed a steady increase in quit rates in people with and without dementia who were prescribed either varenicline or NRT throughout the study's follow-up period (3-months to 4-years). At 2-years follow-up, people with dementia were more likely to quit smoking than those without when prescribed either varenicline or NRT (30.6%, 95% CI: 25.8% to 35.1%) versus (25.7%, 95% CI: 25.4% to 25.8%) (Figure 3) (eTable 3).

For peer review only

For peer review only - http://bmjopen.bmj.com/site/about/guidelines.xhtml

DISCUSSION

We observed sustained higher quit rates in people with dementia after being prescribed either varencline or NRT as compared to those without dementia. Smoking prevalence was lower in people with dementia than those without, and people with dementia were less likely to be prescribed varenicline.

A strength of this study is that we used primary care data from the CPRD which are representative of the UK population.[15] Hence, smoking rates in people with dementia in this study are likely to be generalisable to the dementia population in the UK and in similar countries. Additionally, we used expert-reviewed codelists to define both the exposure and the outcome which would reduce misclassification bias (i.e., classifying people with dementia as people without and vice versa).

Due to the relatively small sample size of people with dementia, we were not able to test the relative effectiveness of varenicline versus NRT on smoking cessation using regression models, for example, and also because of this we were unable to adjust for confounders. We had no data on smokers who purchase over-the-counter NRT, therefore we might be underestimating the prevalence of NRT use, particularly amongst people without dementia as. Hence, it is likely that the prevalence of NRT use amongst people without dementia is much larger than what the prevalence of NRT prescribing in this study. Other limitations include having no information about patient compliance in taking their prescribed smoking cessation medications.

The lower rate of smoking in people with dementia merits further discussion. The smoking rates in the general population has been steadily decreasing, so it is not surprising to observe a similar trend over time in people with dementia, especially considering that a relatively greater

For peer review only - http://bmjopen.bmj.com/site/about/guidelines.xhtml

proportion of smokers might be dying before they get to the age where dementia develops. Other potential explanations for the decreased smoking rates in people with dementia may be that some of them had experienced smoking-related accidents or injuries and they may be under greater supervision by caregivers than before the onset of their illness.[23]

We are not aware of previous population-based studies that estimated the smoking rates amongst people with dementia as the available evidence has been limited to small cross-sectional studies. For instance, a community study in China found that about 17% (N=69/186) of the elderly sample with dementia were current smokers compared to 25% (N=415/1664) in people without dementia.[24] Results from the Toyama dementia survey in Japan show that only 4% of people with dementia smoked compared to 10% in those without.[25] This high variability in the results points to the need for larger and more representative studies in people with dementia to be conducted.

We observed a low prevalence of varenicline prescribing during our study period in people with dementia. This was consistent with findings from a previous study that examined the use of varenicline for smoking cessation treatment in UK primary care using data from THIN database in 2011. Compared to our results from that year, our estimates appear slightly lower (1.1% versus 1.8% in the THIN study).[26] This further corroborates the evidence that varenicline is being underused in people with dementia.[26]

Our study is among the first to estimate the effectiveness of varenicline and NRT for long-term smoking cessation in people with dementia in a real world setting. Our results suggest that both varenicline and NRT are effective in producing long term smoking cessation in people with and without dementia. Almost one third of smokers with dementia quit smoking after 2-years follow-up. Regardless of the smoking cessation medication prescribed to people with dementia, it is

For peer review only - http://bmjopen.bmj.com/site/about/guidelines.xhtml

important to acknowledge that achieving smoking cessation in this group may carry health benefits which would potentially improve their general health status and and/or extend life expectancy.[27] This should ideally be coupled with improving diet quality and increaing physical activites that may shield quitters from weight gain after smoking cessation.[28]

Previous clinical and observational studies have established that varenicline is superior to NRT in achieving smoking cessation in different groups.[12 29 30] Additionally, varenicline did not seem to be associated with an increased risk of documented cardiovascular events, depression, or self-harm when compared with NRT in primary care in England.[31] However, according to a recent systematic review of the literature, older patients with dementia were found to have a low level of medication adherence which raises concerns about the increased risk of hospitalisation or death with non-adherence in this population.[32] On the other hand, a recent study based on CPRD data concluded that NRT appears to increase cardiovascular events for patients prescribed NRT, compared with those receiving smoking cessation advice after 52 weeks of follow-up.[33] This was consistent with the evidence shown by a meta-analysis of 120 studies involving 177, 390 individuals.[34] Further experimental studies are warranted to investigate the safest treatments available for patients attempting smoking cessation.

In summary, people with dementia are more likely to quit smoking when prescribed either varenicline or NRT, as compared to those without dementia. Despite this evidence of effectiveness, we found that people with dementia were less likely to be prescribed varenicline than people without dementia. These findings highlight the need to offer more smoking cessation medication for people with dementia in primary care settings.

For peer review only - http://bmjopen.bmj.com/site/about/guidelines.xhtml

Funding

This research was supported by Global Research Awards for Nicotine Dependence (GRAND), an independently reviewed competitive grants program supported by Pfizer, to the University of Bristol. The funder had no role in study design, data collection and analysis, decision to publish, or preparation of the manuscript.

KHT is funded by a National Institute for Health Research postdoctoral fellowship. TJ receives funding from the National Institute for Health Research Collaboration for Leadership in Applied Health Research and Care (NIHR CLAHRC) West. The views expressed are those of the authors and not necessarily those of the NHS, the NIHR or the Department of Health. RMM is supported by the NIHR Bristol Biomedical Research Centre, a partnership between the University Hospitals Bristol NHS Foundation Trust and the University of Bristol; and by a Cancer Research UK Programme Grant (C18281/A19169).

Data statement

This study is based in part on data from the Clinical Practice Research Datalink obtained under licence from the UK Medicines and Healthcare products Regulatory Agency. The data are provided by patients and collected by the NHS as part of their care and support. The interpretation and conclusions contained in this study are those of the authors alone.

Data and analytic code availability

Codelists that were used for this study are available upon request from the corresponding author.

For peer review only

References

1. Reitsma MB, Fullman N, Ng M, et al. Smoking prevalence and attributable disease burden in 195 countries and territories, 1990-2015: a systematic analysis from the Global Burden of Disease Study 2015. *The Lancet* 2017;389(10082):1885-906. doi: 10.1016/S0140-6736(17)30819-X
2. Almeida OP, Hulse GK, Lawrence D, et al. Smoking as a risk factor for Alzheimer's disease: contrasting evidence from a systematic review of case-control and cohort studies. *Addiction* 2002;97(1):15-28.
3. Anstey KJ, von Sanden C, Salim A, et al. Smoking as a risk factor for dementia and cognitive decline: a meta-analysis of prospective studies. *Am J Epidemiol* 2007;166(4):367-78. doi: 10.1093/aje/kwm116
4. McKenzie J, Bhatti L, Tursan d'Espaignet E. WHO Tobacco Knowledge Summaries: Tobacco and dementia: World Health Organization, 2014.
5. Swan GE, Lessov-Schlaggar CN. The effects of tobacco smoke and nicotine on cognition and the brain. *Neuropsychol Rev* 2007;17(3):259-73. doi: 10.1007/s11065-007-9035-9
6. Saito EK, Diaz N, Chung J, et al. Smoking history and Alzheimer's disease risk in a community-based clinic population. *Journal of Education and Health Promotion* 2017;6:24. doi: 10.4103/jehp.jehp_45_15
7. Takahashi PY, Caldwell CR, Targonski PV. Effect of alcohol and tobacco use on vascular dementia: a matched case control study. *Vascular health and risk management* 2011;7:685-91. doi: 10.2147/vhrm.s26194 [published Online First: 2011/12/06]
8. Heward M, Kelly F. Research and education to understand fire risks associated with dementia: A collaborative case study (innovative practice). *Dementia (London, England)* 2018:1471301218790850. doi: 10.1177/1471301218790850 [published Online First: 2018/08/03]
9. Cataldo JK. Clinical implications of smoking and aging: breaking through the barriers. *J Gerontol Nurs* 2007;33(8):32-41.
10. Choi D, Choi S, Park SM. Effect of smoking cessation on the risk of dementia: a longitudinal study. *Annals of Clinical and Translational Neurology* 2018;5(10):1192-99. doi: doi:10.1002/acn3.633
11. Taylor G, McNeill A, Girling A, et al. Change in mental health after smoking cessation: systematic review and meta-analysis. *BMJ* 2014;348:g1151. doi: 10.1136/bmj.g1151
12. Taylor GMJ, Taylor AE, Thomas KH, et al. The effectiveness of varenicline versus nicotine replacement therapy on long-term smoking cessation in primary care: a prospective cohort study of electronic medical records. *Int J Epidemiol* 2017;46(6):1948-57. doi: 10.1093/ije/dyx109
13. Brose LS, West R, Stapleton JA. Comparison of the effectiveness of varenicline and combination nicotine replacement therapy for smoking cessation in clinical practice. *Mayo Clin Proc* 2013;88(3):226-33. doi: 10.1016/j.mayocp.2012.11.013
14. Cahill K, Stevens S, Perera R, et al. Pharmacological interventions for smoking cessation: an overview and network meta-analysis. *Cochrane Database Syst Rev* 2013(5):CD009329. doi: 10.1002/14651858.CD009329.pub2
15. Herrett E, Gallagher AM, Bhaskaran K, et al. Data Resource Profile: Clinical Practice Research Datalink (CPRD). *Int J Epidemiol* 2015;44(3):827-36. doi: 10.1093/ije/dyv098

For peer review only - http://bmjopen.bmj.com/site/about/guidelines.xhtml

16. Herrett E, Thomas SL, Schoonen WM, et al. Validation and validity of diagnoses in the General Practice Research Database: a systematic review. *Br J Clin Pharmacol* 2010;69(1):4-14. doi: 10.1111/j.1365-2125.2009.03537.x
17. Khan NF, Harrison SE, Rose PW. Validity of diagnostic coding within the General Practice Research Database: a systematic review. *Br J Gen Pract* 2010;60(572):e128-36. doi: 10.3399/bjgp10X483562
18. Davies NM, Taylor G, Taylor AE, et al. What are the effects of varenicline compared with nicotine replacement therapy on long-term smoking cessation and clinically important outcomes? Protocol for a prospective cohort study. *BMJ Open* 2015;5(11)
19. Hernan MA, Alonso A, Logan R, et al. Observational studies analyzed like randomized experiments: an application to postmenopausal hormone therapy and coronary heart disease. *Epidemiology* 2008;19(6):766-79. doi: 10.1097/EDE.0b013e3181875e61
20. West R, Hajek P, Stead L, et al. Outcome criteria in smoking cessation trials: proposal for a common standard. *Addiction* 2005;100(3):299-303. doi: 10.1111/j.1360-0443.2004.00995.x
21. Khan NF, Perera R, Harper S, et al. Adaptation and validation of the Charlson Index for Read/OXMIS coded databases. *BMC Fam Pract* 2010;11:1. doi: 10.1186/1471-2296-11-1
22. Sterne JA, White IR, Carlin JB, et al. Multiple imputation for missing data in epidemiological and clinical research: potential and pitfalls. *BMJ* 2009;338:b2393. doi: 10.1136/bmj.b2393
23. Lester PE, Lyubarova R, Kirtani V, et al. Smoking rates in dementia patients in the outpatient setting. *Arch Gerontol Geriatr* 2011;52(3):281-3. doi: 10.1016/j.archger.2010.04.018
24. Deng J, Cao C, Jiang Y, et al. Prevalence and effect factors of dementia among the community elderly in Chongqing, China. *Psychogeriatrics : the official journal of the Japanese Psychogeriatric Society* 2018;18(5):412-20. doi: 10.1111/psyg.12343 [published Online First: 2018/05/16]
25. Nakahori N, Sekine M, Yamada M, et al. A pathway from low socioeconomic status to dementia in Japan: results from the Toyama dementia survey. *BMC Geriatr* 2018;18(1):102. doi: 10.1186/s12877-018-0791-6
26. Huang Y, Lewis S, Britton J. Use of varenicline for smoking cessation treatment in UK primary care: an association rule mining analysis. *BMC Public Health* 2014;14:1024. doi: 10.1186/1471-2458-14-1024
27. Jha P, Ramasundarahettige C, Landsman V, et al. 21st-century hazards of smoking and benefits of cessation in the United States. *N Engl J Med* 2013;368(4):341-50. doi: 10.1056/NEJMsa1211128
28. Hu Y, Zong G, Liu G, et al. Smoking Cessation, Weight Change, Type 2 Diabetes, and Mortality. *N Engl J Med* 2018;379(7):623-32. doi: 10.1056/NEJMoa1803626
29. Anthenelli RM, Benowitz NL, West R, et al. Neuropsychiatric safety and efficacy of varenicline, bupropion, and nicotine patch in smokers with and without psychiatric disorders (EAGLES): a double-blind, randomised, placebo-controlled clinical trial. *Lancet* 2016;387(10037):2507-20. doi: 10.1016/S0140-6736(16)30272-0
30. Chang PY, Shiu MN, Yuan YT, et al. Comparative effectiveness of varenicline and nicotine replacement therapy for smoking cessation in older and younger smokers: a prospective cohort in Taiwan. *Nicotine Tob Res* 2017 doi: 10.1093/ntr/ntx275

For peer review only - http://bmjopen.bmj.com/site/about/guidelines.xhtml

31. Kotz D, Viechtbauer W, Simpson C, et al. Cardiovascular and neuropsychiatric risks of varenicline: a retrospective cohort study. *Lancet Respir Med* 2015;3(10):761-8. doi: 10.1016/S2213-2600(15)00320-3
32. El-Saifi N, Moyle W, Jones C, et al. Medication Adherence in Older Patients With Dementia: A Systematic Literature Review. *J Pharm Pract* 2018;31(3):322-34. doi: 10.1177/0897190017710524
33. Dollerup J, Vestbo J, Murray-Thomas T, et al. Cardiovascular risks in smokers treated with nicotine replacement therapy: a historical cohort study. *Clinical Epidemiology* 2017;9:231-43. doi: 10.2147/CLEP.S127775
34. Mills EJ, Wu P, Lockhart I, et al. Adverse events associated with nicotine replacement therapy (NRT) for smoking cessation. A systematic review and meta-analysis of one hundred and twenty studies involving 177,390 individuals. *Tob Induc Dis* 2010;8:8. doi: 10.1186/1617-9625-8-8

For peer review only

For peer review only - http://bmjopen.bmj.com/site/about/guidelines.xhtml

Table 1. Baseline characteristics of people with or without dementia by exposure group, N (%)

Characteristic	People with dementia (N=447)			People without dementia (N=234,867)		
	NRT (N=409)	Varenicline (N=38)	Total	NRT (N=159,327)	Varenicline (N=75,540)	Total
Age at time of first prescription[1]	71.1 (12.2)	66.2 (15.1)	70.7 (12.6)	46.2 (15.5)	44.4 (13.2)	45.6 (14.8)
Sex (male)	186 (45.5%)	19 (50.0%)	205 (45.9%)	73,674 (46.2%)	37,676 (49.9%)	111,350 (47.4%)
Index of multiple deprivation score (IMD)[2†]	3	4	3	3	3	3
Number of GP visits 1-year prior to first prescription[1]	11.5 (9.0)	15.3 (9.9)	11.8 (9.1)	8.9 (7.4)	7.3 (6.1)	8.4 (7.0)
BMI[1†]	24.6 (5.1)	25.7 (6.3)	24.7 (5.4)	26.5 (5.7)	26.5 (5.4)	26.5 (5.6)
Year of first prescription[2]	2010	2010	2010	2009	2010	2009
Days of history[1]	3,573.8 (2181.2)	3,450.3 (2327.4)	3,563.3 (2191.5)	3,052.9 (1907.1)	3,164.8 (1986.2)	3,088.9 (1933.6)
Comorbidity ever (Charlson Index)	354 (86.6%)	28 (73.7%)	382 (85.5%)	59,489 (37.3%)	24,017 (31.8%)	83,506 (35.6%)
Alcohol misuse ever	104 (25.4%)	11 (29.0%)	115 (25.7%)	13,890 (8.7%)	4,759 (6.3%)	18,649 (7.9%)
Self-harm ever	67 (16.4%)	9 (23.7%)	76 (17.0%)	17,232 (10.8)	6,652 (8.8%)	23,884 (10.2%)
Ever anxiety and stress related disorders	151 (36.9%)	16 (42.1%)	167 (37.4%)	44,381 (27.9%)	17,377 (23.0%)	61,758 (26.3%)
Other behavioural/neurologic disorder ever	30 (7.3%)	6 (15.8%)	36 (8.1%)	8,693 (5.5%)	2,956 (3.9%)	11,649 (5.0%)
Ever depression	217 (53.1%)	26 (68.4%)	243 (54.4%)	65,343 (41.0%)	26,097 (34.6%)	91,440 (38.9%)
Ever antidepressants	273 (66.7%)	28 (73.7%)	301 (67.3%)	79.584 (50.0%)	32,230 (42.7%)	111,814 (47.6%)
Ever antipsychotics	175 (42.8%)	13 (34.2%)	188 (42.1%)	28,972 (18.2%)	9,792 (13.0%)	38,764 (16.5%)
Ever hypnotics/anxiolytics	238 (58.2%)	20 (52.6%)	258 (57.7%)	60,092 (37.7%)	25,134 (33.3%)	85,226 (36.3%)

1 Data presented are mean and standard deviation. 2 Data presented are median. †Missing data: BMI data was missing for 14.1% (N= 33,059); IMD data was missing for 43.6% (N= 102,657).

For peer review only - http://bmjopen.bmj.com/site/about/guidelines.xhtml

Figure 1. Percentage (%) of primary care patients with an electronic medical record indicating smoking, from 2007 to 2015, in people with or without dementia

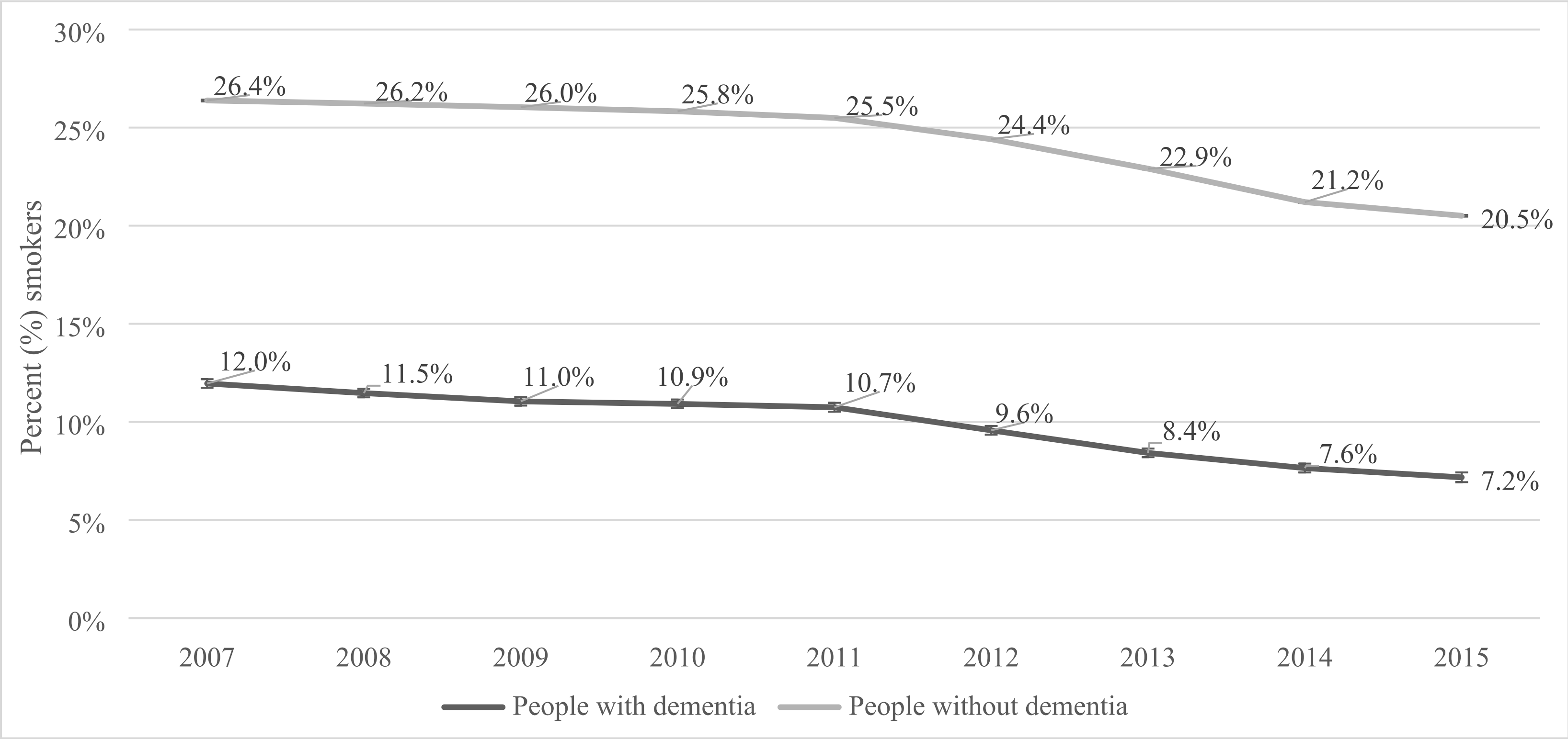

1

Figure 2. Prescription prevalence of varenicline or NRT in primary care, from 2007 to 2015, in smokers with dementia, compared to smokers without dementia

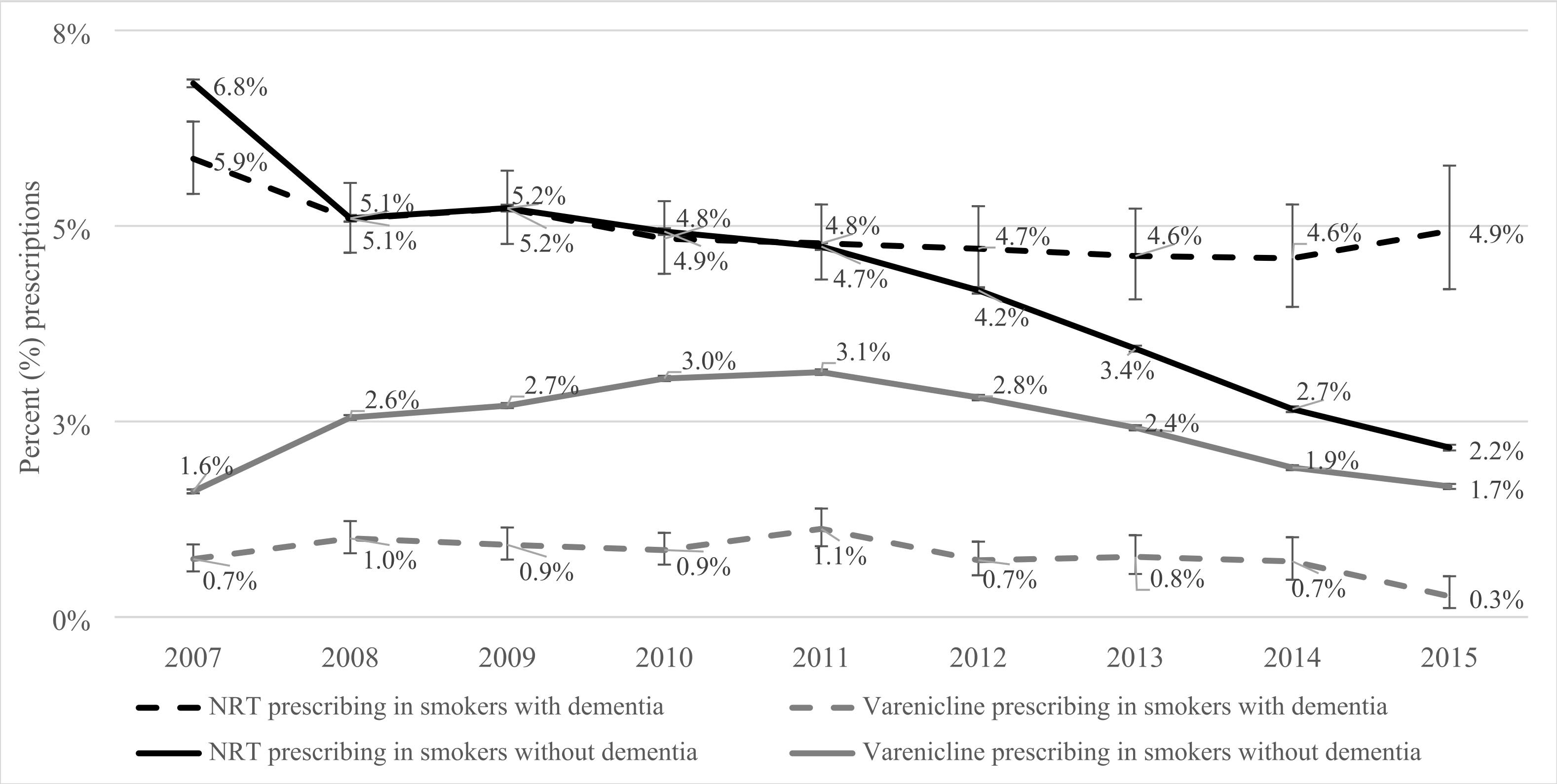

2

Figure 3. Percentage (%) of people with an electronic medical record indicating smoking cessation at up to 2-years follow-up, in people with and without dementia

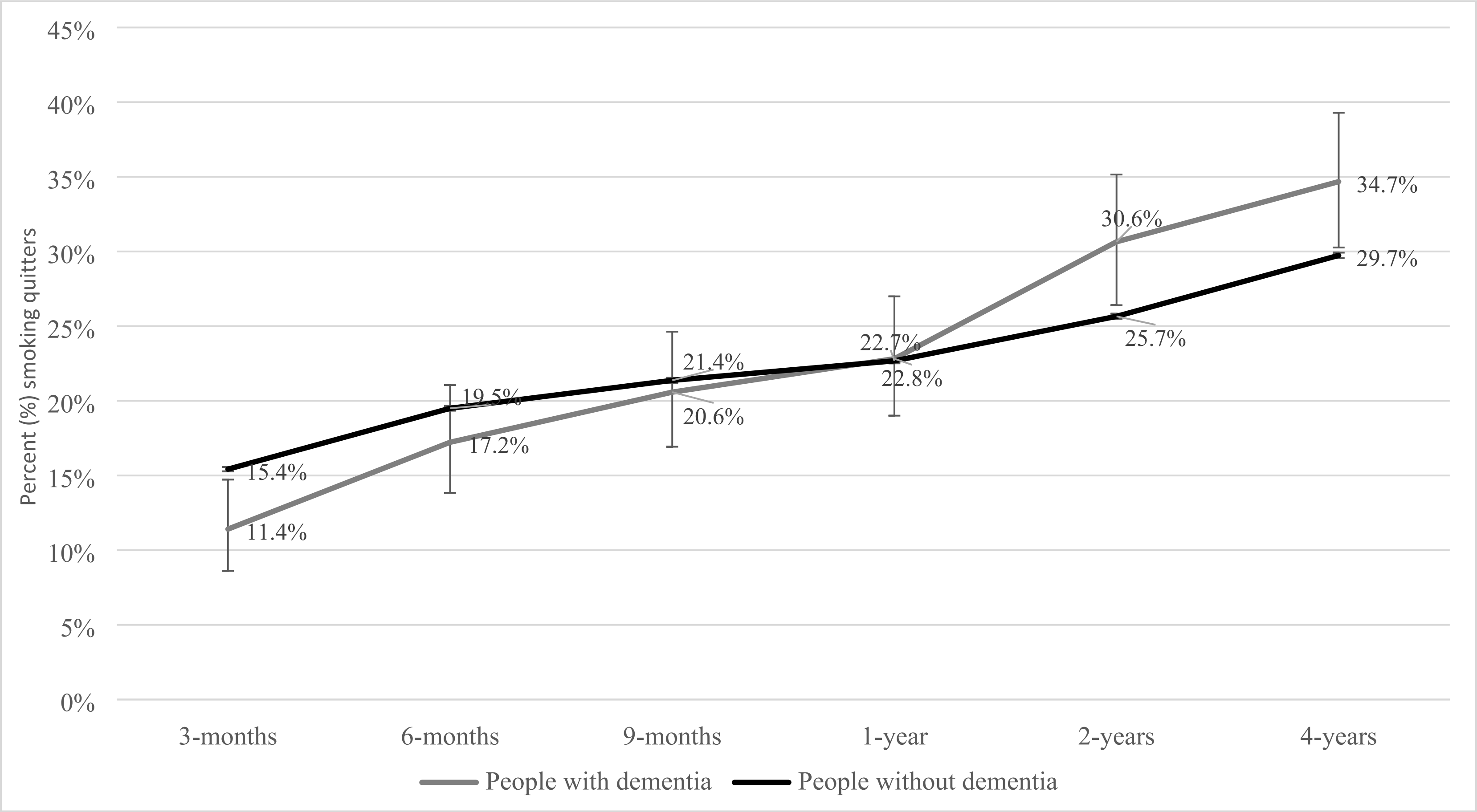

eFigure 1. Flow chart of eligible study participants as per protocol restrictions

Assessed for eligibility
N Records=2,877,296
N Patients=566,936

Excluded from analysis due to protocol restrictions (Davies et al. 2015)

	Prescriptions	Patients
Prescriptions issued to patients under the age of 15	6580	1944
Prescription issued after patients' registration period ended	0	0
Prescription issued before patients' registration period began	296490	53529
Prescription issued to patient aged 16 or 17	13154	4425
Prescription issued before 1st September 2006	822244	145382
Both varenicline and NRT were prescribed on the same day	8289	264
Smoking cessation medication was not prescribed by a general practitioner (GP)	141810	16168
Smoking cessation medication prescription had less than one year of historical follow-up data prior to prescription	163821	41112
Bupropion prescriptions	32997	6981
Prescriptions issued to patients who received a smoking cessation medication issued in the previous 18 months	1069188	30278
Patient had previously received an eligible smoking cessation medication prescription	55870	0
Prescribing GP had seen less than 10 patients.	18494	18494
Patients had less than 180 days of follow-up after 1st prescription.	13045	13045
Total excluded N=	2,641,982	331,622

Included in analysis
N patients=235,314

People with dementia
N patients=447

People without dementia
N patients=234,867

For peer review only - http://bmjopen.bmj.com/site/about/guidelines.xhtml

eTable 1. Distributions of imputed characteristics in the imputation datasets

Characteristic	People with dementia (N=447)							People without dementia (N=234,867)						
	NRT (N=409)		Varenicline (N=38)		Total		% of data imputed	NRT (N=159,327)		Varenicline (N=75,540)		Total		% of data imputed
	Mean	Standard error	Mean	Standard error	Mean	Standard error		Mean	Standard error	Mean	Standard error	Mean	Standard error	
Body mass index	24.6	0.28	26.6	1.02	24.7	0.27	10.3	26.4	0.02	26.5	0.02	26.4	0.01	14.1
Index of multiple deprivation	3.3	0.08	3.3	0.3	3.3	0.09	53.2	3.3	0.004	3.2	0.007	3.3	0.003	43.6

For peer review only - http://bmjopen.bmj.com/site/about/guidelines.xhtml

eTable 2. The likelihood of smokers with ever dementia diagnosis being prescribed varenicline versus NRT, as compared to smokers with no ever dementia diagnosis, N=235,314

	Partially adjusted odds ratio (95% confidence interval) †	Fully adjusted odds ratio (95% confidence interval) ††
Ever dementia diagnosis	0.21 (0.15 to 0.29)	0.26 (0.18 to 0.36)

‡Partially adjusted models were adjusted for: age, sex, year of first prescription.
†† Fully adjusted models were adjusted for: age, sex, days in history, IMD, number of GP visits 1-year prior to first prescription, BMI, year of first prescription, history of major physical morbidity (Charlson Index), alcohol misuse ever, drug misuse ever, depression ever, neurotic disorder ever, self-harm ever, antidepressant prescription ever, antipsychotic prescription ever, hypnotics/anxiolytics prescription ever, other psychotropic medication ever, and other behavioral/neurologic disorder ever. Missing BMI and IMD values were imputed using multiple imputation.
Models were estimated using cluster robust standard errors to account for potential clustering of patients between practices.

For peer review only

eTable 3. Number and percentage (%) of people with an electronic medical record indicating smoking cessation at 3, 6 and 9-months, and 1, 2, and 4-years follow-up, in people with and without dementia

	3-months	6-months	9-months	1-year	2-years	4-years
People without dementia N=234867	36223 (15.4%)	45796 (19.5%)	50193 (21.4%)	53263 (22.7%)	60257 (25.7%)	69842 (29.7%)
People with dementia N=447	51 (11.4%)	77 (17.2%)	92 (20.6%)	102 (22.8%)	137 (30.6%)	155 (34.7%)

For peer review only

For peer review only - http://bmjopen.bmj.com/site/about/guidelines.xhtml

BMJ Open

Use of varenicline and nicotine replacement therapy in people with and without general practitioner-recorded dementia: retrospective cohort study of routine electronic medical records.

Journal:	*BMJ Open*
Manuscript ID	bmjopen-2018-027569.R1
Article Type:	Research
Date Submitted by the Author:	21-Feb-2019
Complete List of Authors:	Itani, Taha; School of Psychological Science,<br>Martin, Richard; University of Bristol, Social Medicine<br>Rai, Dheeraj; University of Bristol, Centre for Academic Mental Health, School of Social & Community Medicine<br>Jones, Tim; NIHR CLAHRC West,<br>Taylor, Gemma; University of Birmingham, Department of Primary Care Clinical Sciences<br>Thomas, Kyla; University of Bristol, School of Social and Community Medicine<br>Munafo, Marcus; University of Bristol, Experimental Psychology<br>Davies, Neil; University of Bristol,<br>Taylor, Amy; 5. School of Experimental Psychology University of Bristol,
**Primary Subject Heading**:	Smoking and tobacco
Secondary Subject Heading:	Epidemiology
Keywords:	Dementia < NEUROLOGY, Smoking cessation, Smoking prevalence

SCHOLARONE™
Manuscripts

PAPER TITLE

Use of varenicline and nicotine replacement therapy in people with and without general practitioner-recorded dementia: retrospective cohort study of routine electronic medical records.

Authors

Taha Itani PhD[1,2]; Richard M Martin PhD [1,3,4]; Dheeraj Rai PhD[3,5,6], Tim Jones PhD[7]; Gemma MJ Taylor PhD[8] Kyla H Thomas PhD[9]; Marcus R Munafò PhD[1,2]; Neil M Davies PhD[1,4] and Amy E Taylor PhD[3,4]

Author affiliations

1 Medical Research Council Integrative Epidemiology Unit at the University of Bristol, BS8 2BN, United Kingdom.

2 UK Centre for Tobacco and Alcohol Studies, School of Psychological Science, University of Bristol, 12a Priory Road, Bristol, BS8 1TU, United Kingdom.

3 NIHR Biomedical Research Centre at the University Hospitals Bristol NHS Foundation Trust and the University of Bristol, United Kingdom.

4 Population Health Sciences, Bristol Medical School, University of Bristol, Oakfield Grove, Bristol, BS8 2BN, United Kingdom.

5 Centre for Academic Mental Health; Bristol Medical School, University of Bristol, Barley House, Oakfield Grove, Bristol, BS8 2BN, United Kingdom.

6 Avon & Wiltshire Partnership NHS Mental Health Trust, Bristol, UK

7 National Institute for Health Research Collaboration for Leadership in Applied Health Research and Care West (NIHR CLAHRC West) at University Hospitals Bristol NHS Foundation Trust, 9th Floor Whitefriars, Lewins Mead, Bristol, BS1 2NT, United Kingdom.

8 Addiction and Mental Health Group (AIM) Department of Psychology, University of Bath, Claverton Down, Bath, BA2 7AY.

9 Population Health Sciences, Bristol Medical School, University of Bristol, Canynge Hall, Bristol, BS8 2PS, United Kingdom

For peer review only - http://bmjopen.bmj.com/site/about/guidelines.xhtml

Author Positions

Taha Itani, Research Associate in Epidemiology; Richard M Martin, Professor of Clinical Epidemiology; Dheeraj Rai, Consultant Senior Lecturer in Psychiatry; Tim Jones, Senior Research Associate; Gemma MJ Taylor, Lecturer in Clinical Psychology; Kyla H Thomas, Consultant Senior Lecturer in Public Health Medicine and NIHR Postdoctoral Fellow; Marcus R Munafò, Professor of Biological Psychology; Neil M Davies, Senior Research Fellow and Amy E Taylor, Research Fellow.

Correspondence

Dr Taha Itani, ti17926@bristol.ac.uk

Word count

2,991

review only

For peer review only - http://bmjopen.bmj.com/site/about/guidelines.xhtml

ABSTRACT

Objectives: Our primary objective was to estimate smoking prevalence and prescribing rates of varenicline and NRT in people with and without GP-recorded dementia. Our secondary objective was to assess and compare quit rates of smokers with versus without general practitioner (GP)-recorded dementia who were prescribed varenicline or nicotine replacement therapy (NRT) for smoking cessation.

Design: A retrospective cohort study based on the analysis of electronic medical records within the Clinical Practice Research Datalink (2007-2015).

Setting: 683 general practices in England.

Participants: People with and without GP-recorded dementia, aged 18 years and have a code indicating that they are a current smoker.

Intervention: Index prescription of varenicline or NRT (from 1st September 2006).

Outcome measures: The primary outcomes were smoking prevalence and prescribing rates of varenicline and NRT (2007-2015). The secondary outcome was smoking cessation at 2 years.

Results: Age and sex-standardised prevalence of smoking was slightly higher in people with GP-recorded dementia than in those without. There were 235,314 people aged 18 years and above prescribed NRT or varenicline. Amongst smokers with GP-recorded dementia (N=447), 409 were prescribed NRT and 38 varenicline. Smokers with GP-recorded dementia were 74% less likely (95% confidence interval: 64% to 82%) to be prescribed varenicline than NRT, compared to smokers without GP-recorded dementia. Compared to people without GP-recorded dementia, people with GP-recorded dementia had consistently lower prescribing rates of varenicline from 2007 to 2015.

Two years after prescription, there was no clear evidence for a difference in the likelihood of smoking cessation after prescription of these medications between individuals with and without dementia (OR 1.0, 0.8, 1.2).

For peer review only - http://bmjopen.bmj.com/site/about/guidelines.xhtml

Conclusions: Between 2007 and 2015, people with GP-recorded dementia were less likely to be prescribed varenicline than those without dementia. Quit rates following prescription of either NRT or varenicline were similar in those with and without dementia.

Strengths and limitations of this study

- This study used primary care data from the Clinical Practice Research Datalink (CPRD) which are representative of the UK primary care population.
- Expert-reviewed codelists were developed to define both the exposure and the outcome which would reduce misclassification bias.
- Due to the small sample size of people with GP-recorded dementia, we were not able to test the relative effectiveness of varenicline versus NRT on smoking cessation using regression models.
- Data on smokers who purchase over-the-counter NRT were not available, and therefore the prevalence of NRT might be underestimated.

For peer review only

INTRODUCTION

Smoking is a leading cause of mortality and morbidity worldwide. About 12% of global deaths were linked to smoking in 2015.[1] There is substantial evidence that smoking is associated with an increased risk of developing dementia.[2 3] For instance, it is estimated that 14% of Alzheimer's disease (AD) cases worldwide are attributable to smoking.[4] Smoking is thought to accelerate the onset of dementia mainly via vascular risk factors such as narrowing of blood vessels in the heart and the brain, thereby triggering oxidative stress.[4 5]

Few studies report smoking prevalence among people with dementia. In a cross-sectional analysis of patients treated for AD in a neurology clinic during a 2-year period, past smoking prevalence was 29% (N=21/72).[6] In a case-control study of patients with vascular dementia, the rate of current tobacco use was 9% (N=17/198) as compared to 6% (N=11/199) in the control group.[7] Beyond the harmful health effects of smoking in people with dementia, there are concerns that smokers in this group may have a higher likelihood of fire accidents due to their compromised cognitive state.[8]

Since there are currently no available treatments to cure dementia, there is a growing interest in identifying modifiable risk factors for reducing the occurrence of the disease, to delay dementia onset, and reduce its burden.[4 9] Smoking cessation could potentially decrease the risk or slow the development of dementia[10] and could improve the quality of life of older adults through improved physical, and mental wellbeing.[9 11] Little is known about whether people with dementia are prescribed smoking cessation agents and whether they are effective in this group.

For peer review only - http://bmjopen.bmj.com/site/about/guidelines.xhtml

Based on a Cochrane review of 136 trials, it was reported that NRT (compared to placebo or no treatment) can help people who make a quit attempt to increase their chances of successfully stopping smoking (Hartmann-Boyce et al., 2018). Data from observational studies [12 13] and meta-analyses of randomized controlled trials[14] indicate that varenicline is more effective than single form nicotine replacement therapy (NRT) for smoking cessation in the general population. However, it is unclear whether varenicline or NRT could help smokers with dementia to quit smoking.

Therefore in this study we aimed to: 1) describe the rates of smoking prevalence and smoking cessation medication prescribing amongst people with and without GP-recorded dementia in UK primary care settings from 2007 to 2015; and; 2) assess and compare quit rates of varenicline and NRT on smoking cessation in people with GP-recorded dementia, compared to those without, at 3, 6, 9 months and 1, 2, 4 years after first prescription.

For peer review only - http://bmjopen.bmj.com/site/about/guidelines.xhtml

METHODS

Data source and population

We conducted a retrospective cohort study using electronic medical records from 683 general practices in England from 2007 to 2015 using data from the Clinical Practice Research Database (CPRD). Patient data from the CPRD are broadly representative of the UK primary care population in terms of age, sex and ethnicity.[15] These data have been validated, audited, and quality checked.[16] The study's protocol (15_115R) was approved by the Independent Scientific Advisory Committee for MHRA Database Research (https://www.cprd.com/isac/).

Code lists

We defined variables using medical and product codes within the CPRD. All code lists were developed using a list from a previously published study.[12] These codelists were derived from the the British National Formulary (BNF) and the International Classification of Diseases (ICD-10) and then agreed upon by field experts (DR, KHT). A previous systematic review that checked the validity of coding of various diagnoses in what was then the General Practice Research Database (now CPRD) suggests that coding for dementia and Alzheimer's is relatively accurate.[17]

Study subjects

During the study period (2007 to 2015), we included people (aged ≥18 years) with information about their smoking status (either smokers or non-smokers) for smoking prevalence estimates, and we included smokers prescribed either varenicline or NRT for prescribing prevalence and for

For peer review only - http://bmjopen.bmj.com/site/about/guidelines.xhtml

comparing quit rates. We used an open cohort design, with new patients entering the cohort throughout the observation period.

For the primary objective, people were categorised as having dementia if: i) they had ever been diagnosed with dementia (based on ICD-10 diagnoses F00-F03), or ii) if they were prescribed dementia medications: (BNF chapter 4.11) (see supplementary file for a list of all Read codes used in this study). Patients with no records of the above-mentioned diagnosis/prescriptions were considered to have no GP-recorded dementia (for clarity, we hence forth refer to this as dementia).

For the secondary objective, we constructed a cohort of eligible first varenicline/NRT prescriptions (see eFigure 1 for a flow chart of numbers of patients excluded and reasons for exclusion). Within that cohort of eligible prescriptions, we categorised dementia for those with recorded Read codes for ever dementia/dementia medications prior to first varenicline/NRT prescription; we did this to ensure that a diagnosis of dementia preceded the exposure (prescription of a smoking cessation medicine).

Variables

Smoking and prescribing prevalence estimates

For smoking prevalence estimates, a patient's smoking status (aged ≥18 years) was defined by a record indicating smoking/non-smoking or prescription of NRT/varenicline in that year. In case of missing information about smoking, the patient's smoking status was carried forward until there was evidence of a change in smoking status or carried backwards if smoking status was

For peer review only - http://bmjopen.bmj.com/site/about/guidelines.xhtml

For peer review only

only recorded in the final year of registration. Records that were outside the registration period for each patient were excluded.

Prevalence of prescriptions of varenicline and NRT was calculated by dividing the number of prescriptions each year from 2007 to 2015 (there were very few varenicline prescriptions for patients with dementia in 2006) by the number of current smokers in each year. In both instances, prevalence was estimated for people with and without dementia. Individuals with missing smoking information were excluded from the denominators.

For peer review only

Exposure

Exposure was defined as prescription of varenicline or NRT (e.g., patches, etc. on prescription as opposed to over-the-counter, hence forth we refer to this as NRT).

Prescriptions used to define exposure groups were issued between September 1st, 2006 and August 31st, 2016, with no prior record of use of a related product in the preceding 18-months. We used the first treatment episode to ensure that intervention groups were "new users" of the medication.[18] We did not model multiple and repeated prescriptions of smoking cessation medications during follow-up because this is likely to be strongly related to patient characteristics.

Outcome: smoking cessation

Smoking cessation was defined as having an electronic record indicating a non-smoking status. The closest smoking record to each follow-up period was selected to determine each study participant's smoking status; i.e., the most recent smoking record identified between cohort entry and each follow-up period (e.g., 3-months, 6-months). People with missing smoking data (beyond 180 days) were assumed to be continuing smokers[19] which has been previously found to be robust in sensitivity analyses.[12]

For peer review only

For peer review only - http://bmjopen.bmj.com/site/about/guidelines.xhtml

Covariates

Covariates included patients' age at time of prescription, sex, index of multiple deprivation score (IMD), mean number of GP visits one year prior to first prescription, year of first prescription of a smoking cessation medication, body mass index (BMI), days registered in the CPRD, the Charlson Index (a measure of chronic illness),[20] alcohol misuse, history of mental disorder or psychoactive medication prescriptions, evidence of other psychoactive medication prescription or other less common psychiatric disorders. We used multiple imputation to handle missing data on BMI and IMD. This was done using the ICE command in Stata where we produced 20 imputed datasets (eTable 1). We included all exposures, covariates, and outcomes in the imputation model.[21]

Follow-up

The secondary outcome was smoking cessation at 2 years, and this was also assessed at 3, 6, 9 months and 1, and 4 years after first prescription of varenicline or NRT.

Statistical analysis

Smoking prevalence

Smoking rates were calculated by dividing the number of people with dementia who had Read codes indicating current smoking for each year between 2007 and 2015 by the total number of people with dementia and a smoking status code (indicating current or non/ex-smoking) each year between 2007 and 2015. For comparison, smoking prevalence was also estimated amongst people without dementia.

For peer review only - http://bmjopen.bmj.com/site/about/guidelines.xhtml

Prescribing prevalence

The prevalence of varenicline and NRT prescribing amongst current smokers was calculated by dividing the number of prescriptions each year from 2007 to 2015 (there were no varenicline prescriptions for patients with dementia in 2006) by the number of current smokers in each year. Prevalence was estimated for people with and without dementia.

Association of varenicline and NRT prescriptions with smoking cessation

The effectiveness of varenicline and NRT on smoking cessation was determined by estimating quit rates at each follow-up period. This was calculated by dividing the number of non-smokers in each group by the total number of people in that group at each follow-up period. All analyses were conducted using Stata 14 MP.

Patient and Public Involvement

This study was based on the analysis of anonymised primary care data. No patients were involved during the design and analysis of this study.

For peer review only

For peer review only - http://bmjopen.bmj.com/site/about/guidelines.xhtml

RESULTS

Smoking prevalence and smoking cessation medication prescribing estimates

Unadjusted smoking prevalence amongst people with dementia steadily decreased from 12% (N=10,121/84,647 in 2007 to 7% (N=3,018/42,075) in 2015 (Figure 1). These estimates were consistently lower than in people without dementia, 26% (N= 1,003,374/ 3,802,954) in 2007 and 21% (N=628,116/3,062,917) in 2015 respectively (eTable 2). However, after age and sex standardization, the smoking prevalence amongst people with dementia was slightly higher than in people without (eFigure 2).

The rate of NRT prescribing in people without dementia was 5% (51,367/1,007,563) in 2008 which decreased to 2% (N=13,607/628,116) by 2015, whereas little fluctuation was observed (remained at 5%) in NRT prescribing rates amongst people with dementia during the same period. Compared to people without dementia, people with dementia had lower prescribing rates of varenicline (Figure 2).

Smoking cessation amongst individuals prescribed NRT and Varenicline

Of the 235,314 people included in this analysis, 447 were people with dementia, whereas 234,867 were people without (eFigure 1). Overall, 159,736 smokers were prescribed NRT and 75,578 prescribed varenicline (Table 1). The mean age of people with dementia at the time of smoking medication prescription was about 72 years (SD=12.6), while that of people without dementia was 46 years (SD=14.8) People with dementia were about 25 years older, had more GP visits 1-year prior to the first prescription, suffered from more comorbidities, and received more mental health-related prescriptions than those without dementia (Table 1).

For peer review only - http://bmjopen.bmj.com/site/about/guidelines.xhtml

Smokers with dementia were 74% (95% confidence interval: 64% to 82%) less likely to be prescribed varenicline than NRT, compared to smokers without dementia (eTable 3. We observed a steady increase in quit rates in people with and without dementia who were prescribed either varenicline or NRT throughout the study's follow-up period (3-months to 4-years). At 2 years follow-up, people with dementia were more likely to quit smoking (30.6%, 95% CI: 25.8% to 35.1%) than those without (25.7%, 95% CI: 25.4% to 25.8%) when prescribed either varenicline or NRT   (Figure 3) (eTable 4). After adjusting for all covariates, we found no evidence for a difference in quit rates between individuals with and without dementia (OR=1.0, 95% CI: 0.81-1.23) (eTable 5).

For peer review only

For peer review only - http://bmjopen.bmj.com/site/about/guidelines.xhtml

DISCUSSION

People with dementia were less likely to be prescribed varenicline compared to those without dementia. There was no clear evidence for a difference in quit rates in individuals with and without dementia following prescription of NRT or varenicline.

A strength of this study is that we used primary care data from the CPRD which are representative of the UK primary care population.[15] Hence, smoking rates in people with dementia in this study are likely to be generalisable to the dementia population in the UK and in similar countries. Additionally, we used expert-reviewed codelists to define both the exposure and the outcome which would reduce misclassification bias (i.e., classifying people with dementia as people without and vice versa).

There are several limitations to this research. Due to the small sample size of people with dementia, we were not able to test the relative effectiveness of varenicline versus NRT on smoking cessation using regression models. We had no data on smokers who purchase over-the-counter NRT, therefore we might be underestimating the prevalence of NRT use, particularly amongst people without dementia. Hence, it is likely that the prevalence of NRT use amongst people without dementia is larger than the prevalence of NRT prescribing in this study. Moreover, outcome definition (smoking vs. non-smoking status) was based on self-reported data rather than biochemical verification of smoking status. Additionally, social desirability bias may occur when unsuccessful quitters don't disclose their smoking status truthfully. We also relied on point estimates (i.e. smoking status reported at a single timepoint) for making conclusions about smoking status. This may not have captured long-term abstinence. In other words, it is possible that smoking status may have fluctuated between GP visitsA further limitation is having no information about patient adherence in taking their prescribed smoking cessation medications.

For peer review only - http://bmjopen.bmj.com/site/about/guidelines.xhtml

We are not aware of previous population-based studies that estimated the smoking rates amongst people with dementia as the available evidence has been limited to small cross-sectional studies. For instance, a community study in China found that about 17% (N=69/186) of the elderly sample with dementia were current smokers compared to 25% (N=415/1664) in people without dementia.[22] Results from the Toyama dementia survey in Japan show that only 4% of people with dementia smoked compared to 10% in those without.[23] This high variability in the results points to the need for larger and more representative studies in people with dementia to be conducted. Meanwhile, we found that smoking prevalence has decreased steadily amongst people without dementia, from 26.4% in 2007 to 20.5% in 2015. This was fairly similar to the general population in England as evidenced by results from the Smoking Toolkit Study (24.2% in 2007 to 18.7% in 2015)[24] and therefore speaks to the external validity of our study.

We observed a low prevalence of varenicline prescribing during our study period in people with dementia. Our estimate for individuals without dementia was consistent with findings from a previous study that examined the use of varenicline for smoking cessation treatment in UK primary care using data from THIN database in 2011. Compared to our results from that year, our estimates appear slightly lower (1.1% versus 1.8% in the THIN study).[25] While NRT prescribing rates remained at similar levels in people with dementia between 2007 and 2015, these rates declined over time in people without dementia. A recent report from the British Lung Foundation found that NRT prescribing through primary care in England has dropped about 75% during the last 10 years. That was mainly due to cuts to public health funding that would have adversely impacted specialist stop smoking services.[26]

Our study is among the first to investigate longer term smoking cessation after being prescribed varenicline and NRT amongst individuals with dementia. Our results suggest that both

For peer review only - http://bmjopen.bmj.com/site/about/guidelines.xhtml

varenicline and NRT could produce long term smoking cessation in people with and without dementia. Almost one third of smokers with dementia quit smoking after 2 years follow-up. Regardless of the smoking cessation medication prescribed to people with dementia, it is important to acknowledge that achieving smoking cessation in this group may carry health benefits which would potentially improve their general health status and and/or extend life expectancy.[27] This should ideally be coupled with improving diet quality and increaing physical activites that may shield quitters from weight gain after smoking cessation.[28]

It is not clear why individuals with dementia are less likely to be prescribed varenicline than NRT compared to individuals without. Previous clinical and observational studies have established that varenicline is superior to single form NRT in achieving smoking cessation in different groups.[12 29 30] Additionally, varenicline did not seem to be associated with an increased risk of documented cardiovascular events, depression, or self-harm when compared with NRT in primary care in England.[31] On the other hand, a recent study based on CPRD data concluded that NRT appears to increase cardiovascular events for patients prescribed NRT, compared with those receiving smoking cessation advice after 52 weeks of follow-up.[32] This was consistent with the evidence shown by a meta-analysis of 120 studies involving 177, 390 individuals.[33] It is possible that GPs are less likely to prescribe varenicline to individuals with dementia because of lower likelihood of adherence; in a recent systematic review of the literature, older patients with dementia were found to have a low level of medication adherence.[34]

In summary, age- and sex-adjusted smoking prevalence amongst individuals with dementia was similar to those without dementia and smoking cessation rates were similar following prescription of smoking cessation medications between these groups. However, individuals with dementia were less likely to be prescribed varenlicline than individuals without dementia.

For peer review only - http://bmjopen.bmj.com/site/about/guidelines.xhtml

Funding

This research was supported by Global Research Awards for Nicotine Dependence (GRAND), an independently reviewed competitive grants program supported by Pfizer, to the University of Bristol. The funder had no role in study design, data collection and analysis, decision to publish, or preparation of the manuscript.

KHT is funded by a National Institute for Health Research postdoctoral fellowship (PDF-2017-10-068). TJ receives funding from the National Institute for Health Research Collaboration for Leadership in Applied Health Research and Care (NIHR CLAHRC) West. The views expressed are those of the authors and not necessarily those of the NHS, the NIHR or the Department of Health. RMM is supported by the NIHR Bristol Biomedical Research Centre, a partnership between the University Hospitals Bristol NHS Foundation Trust and the University of Bristol; and by a Cancer Research UK Programme Grant (C18281/A19169). GT is funded by a Cancer Research UK Population Researcher Postdoctoral Fellowship Award (C56067/A21330).

Data statement

This study is based in part on data from the Clinical Practice Research Datalink obtained under licence from the UK Medicines and Healthcare Products Regulatory Agency. The data are provided by patients and collected by the NHS as part of their care and support. The interpretation and conclusions contained in this study are those of the authors alone.

Data and analytic code availability

Codelists that were used for this study are available in the supplementary file (eTables 6 and 7).

For peer review only - http://bmjopen.bmj.com/site/about/guidelines.xhtml

Contributorship Statement

TI contributed to data cleaning, data analysis, interpretation of results and writing the manuscript. RMM, GT, ND, AT and KT contributed to study conceptualization, study design, interpretation of results, data analysis and writing the manuscript. MM, and DR contributed to study conceptualization, study design, interpretation of results and writing the manuscript. TJ extracted the data and contributed to writing the manuscript. TI, AT, and ND had full access to all of the data in the study and takes responsibility for the integrity of the data and the accuracy of the data analysis.

Figure legends

Figure 1. Percentage (%) of primary care patients with an electronic medical record indicating smoking, from 2007 to 2015, in people with or without dementia

Figure 2. Prescription prevalence of varenicline or NRT in primary care, from 2007 to 2015, in smokers with dementia, compared to smokers without dementia

Figure 3. Percentage (%) of people with an electronic medical record indicating smoking cessation at up to 2 years follow-up, in people with and without dementia

For peer review only

For peer review only - http://bmjopen.bmj.com/site/about/guidelines.xhtml

References

1. Reitsma MB, Fullman N, Ng M, et al. Smoking prevalence and attributable disease burden in 195 countries and territories, 1990-2015: a systematic analysis from the Global Burden of Disease Study 2015. *The Lancet* 2017;389(10082):1885-906. doi: 10.1016/S0140-6736(17)30819-X
2. Almeida OP, Hulse GK, Lawrence D, et al. Smoking as a risk factor for Alzheimer's disease: contrasting evidence from a systematic review of case-control and cohort studies. *Addiction* 2002;97(1):15-28.
3. Anstey KJ, von Sanden C, Salim A, et al. Smoking as a risk factor for dementia and cognitive decline: a meta-analysis of prospective studies. *Am J Epidemiol* 2007;166(4):367-78. doi: 10.1093/aje/kwm116
4. McKenzie J, Bhatti L, Tursan d'Espaignet E. WHO Tobacco Knowledge Summaries: Tobacco and dementia: World Health Organization, 2014.
5. Swan GE, Lessov-Schlaggar CN. The effects of tobacco smoke and nicotine on cognition and the brain. *Neuropsychol Rev* 2007;17(3):259-73. doi: 10.1007/s11065-007-9035-9
6. Saito EK, Diaz N, Chung J, et al. Smoking history and Alzheimer's disease risk in a community-based clinic population. *Journal of Education and Health Promotion* 2017;6:24. doi: 10.4103/jehp.jehp_45_15
7. Takahashi PY, Caldwell CR, Targonski PV. Effect of alcohol and tobacco use on vascular dementia: a matched case control study. *Vascular health and risk management* 2011;7:685-91. doi: 10.2147/vhrm.s26194 [published Online First: 2011/12/06]
8. Heward M, Kelly F. Research and education to understand fire risks associated with dementia: A collaborative case study (innovative practice). *Dementia (London, England)* 2018:1471301218790850. doi: 10.1177/1471301218790850 [published Online First: 2018/08/03]
9. Cataldo JK. Clinical implications of smoking and aging: breaking through the barriers. *J Gerontol Nurs* 2007;33(8):32-41.
10. Choi D, Choi S, Park SM. Effect of smoking cessation on the risk of dementia: a longitudinal study. *Annals of Clinical and Translational Neurology* 2018;5(10):1192-99. doi: doi:10.1002/acn3.633
11. Taylor G, McNeill A, Girling A, et al. Change in mental health after smoking cessation: systematic review and meta-analysis. *BMJ* 2014;348:g1151. doi: 10.1136/bmj.g1151
12. Taylor GMJ, Taylor AE, Thomas KH, et al. The effectiveness of varenicline versus nicotine replacement therapy on long-term smoking cessation in primary care: a prospective cohort study of electronic medical records. *Int J Epidemiol* 2017;46(6):1948-57. doi: 10.1093/ije/dyx109
13. Brose LS, West R, Stapleton JA. Comparison of the effectiveness of varenicline and combination nicotine replacement therapy for smoking cessation in clinical practice. *Mayo Clin Proc* 2013;88(3):226-33. doi: 10.1016/j.mayocp.2012.11.013
14. Cahill K, Stevens S, Perera R, et al. Pharmacological interventions for smoking cessation: an overview and network meta-analysis. *Cochrane Database Syst Rev* 2013(5):CD009329. doi: 10.1002/14651858.CD009329.pub2
15. Herrett E, Gallagher AM, Bhaskaran K, et al. Data Resource Profile: Clinical Practice Research Datalink (CPRD). *Int J Epidemiol* 2015;44(3):827-36. doi: 10.1093/ije/dyv098

For peer review only - http://bmjopen.bmj.com/site/about/guidelines.xhtml

16. Herrett E, Thomas SL, Schoonen WM, et al. Validation and validity of diagnoses in the General Practice Research Database: a systematic review. *Br J Clin Pharmacol* 2010;69(1):4-14. doi: 10.1111/j.1365-2125.2009.03537.x
17. Khan NF, Harrison SE, Rose PW. Validity of diagnostic coding within the General Practice Research Database: a systematic review. *Br J Gen Pract* 2010;60(572):e128-36. doi: 10.3399/bjgp10X483562
18. Hernan MA, Alonso A, Logan R, et al. Observational studies analyzed like randomized experiments: an application to postmenopausal hormone therapy and coronary heart disease. *Epidemiology* 2008;19(6):766-79. doi: 10.1097/EDE.0b013e3181875e61
19. West R, Hajek P, Stead L, et al. Outcome criteria in smoking cessation trials: proposal for a common standard. *Addiction* 2005;100(3):299-303. doi: 10.1111/j.1360-0443.2004.00995.x
20. Khan NF, Perera R, Harper S, et al. Adaptation and validation of the Charlson Index for Read/OXMIS coded databases. *BMC Fam Pract* 2010;11:1. doi: 10.1186/1471-2296-11-1
21. Sterne JA, White IR, Carlin JB, et al. Multiple imputation for missing data in epidemiological and clinical research: potential and pitfalls. *BMJ* 2009;338:b2393. doi: 10.1136/bmj.b2393
22. Deng J, Cao C, Jiang Y, et al. Prevalence and effect factors of dementia among the community elderly in Chongqing, China. *Psychogeriatrics : the official journal of the Japanese Psychogeriatric Society* 2018;18(5):412-20. doi: 10.1111/psyg.12343 [published Online First: 2018/05/16]

23. Nakahori N, Sekine M, Yamada M, et al. A pathway from low socioeconomic status to dementia in Japan: results from the Toyama dementia survey. *BMC Geriatr* 2018;18(1):102. doi: 10.1186/s12877-018-0791-6
24. Smoking in England (2017). Latest trends on smoking in England from the Smoking Toolkit Study. Available at: http://www.smokinginengland.info/sts-documents/
25. Huang Y, Lewis S, Britton J. Use of varenicline for smoking cessation treatment in UK primary care: an association rule mining analysis. *BMC Public Health* 2014;14:1024. doi: 10.1186/1471-2458-14-102426. British Lung Foundation (2018). Available at: https://cdn.shopify.com/s/files/1/0221/4446/files/Tobacco_Report_July_2018_PDF.pdf?578912944904678086 9&_ga=2.142198583.422598499.1547211481-1798507517.1544436747
27. Jha P, Ramasundarahettige C, Landsman V, et al. 21st-century hazards of smoking and benefits of cessation in the United States. *N Engl J Med* 2013;368(4):341-50. doi: 10.1056/NEJMsa1211128
28. Hu Y, Zong G, Liu G, et al. Smoking Cessation, Weight Change, Type 2 Diabetes, and Mortality. *N Engl J Med* 2018;379(7):623-32. doi: 10.1056/NEJMoa1803626
29. Anthenelli RM, Benowitz NL, West R, et al. Neuropsychiatric safety and efficacy of varenicline, bupropion, and nicotine patch in smokers with and without psychiatric disorders (EAGLES): a double-blind, randomised, placebo-controlled clinical trial. *Lancet* 2016;387(10037):2507-20. doi: 10.1016/S0140-6736(16)30272-0

For peer review only - http://bmjopen.bmj.com/site/about/guidelines.xhtml

30. Chang PY, Shiu MN, Yuan YT, et al. Comparative effectiveness of varenicline and nicotine replacement therapy for smoking cessation in older and younger smokers: a prospective cohort in Taiwan. *Nicotine Tob Res* 2017 doi: 10.1093/ntr/ntx275
31. Kotz D, Viechtbauer W, Simpson C, et al. Cardiovascular and neuropsychiatric risks of varenicline: a retrospective cohort study. *Lancet Respir Med* 2015;3(10):761-8. doi: 10.1016/S2213-2600(15)00320-3
32. Dollerup J, Vestbo J, Murray-Thomas T, et al. Cardiovascular risks in smokers treated with nicotine replacement therapy: a historical cohort study. *Clinical Epidemiology* 2017;9:231-43. doi: 10.2147/CLEP.S127775
33. Hartmann-Boyce J, Chepkin SC, et al. Nicotine replacement therapy versus control for smoking cessation.*Cochrane Database of Systematic Reviews* 2018. doi: 10.1002/14651858.CD000146.pub5
34. El-Saifi N, Moyle W, Jones C, et al. Medication Adherence in Older Patients With Dementia: A Systematic Literature Review. *J Pharm Pract* 2018;31(3):322-34. doi: 10.1177/0897190017710524

For peer review only

Table 1. Baseline characteristics of people with or without dementia by exposure group, N (%)

Characteristic	People with dementia (N=447)			People without dementia (N=234,867)		
	**NRT (N=409)**	**Varenicline (N=38)**	**Total**	**NRT (N=159,327)**	**Varenicline (N=75,540)**	**Total**
Age at time of first prescription[1]	71.1 (12.2)	66.2 (15.1)	70.7 (12.6)	46.2 (15.5)	44.4 (13.2)	45.6 (14.8)
Sex (male)	186 (45.5%)	19 (50.0%)	205 (45.9%)	73,674 (46.2%)	37,676 (49.9%)	111,350 (47.4%)
Index of multiple deprivation score (IMD)[2†]	3	4	3	3	3	3
Number of GP visits 1-year prior to first prescription[1]	11.5 (9.0)	15.3 (9.9)	11.8 (9.1)	8.9 (7.4)	7.3 (6.1)	8.4 (7.0)
BMI[1†]	24.6 (5.1)	25.7 (6.3)	24.7 (5.4)	26.5 (5.7)	26.5 (5.4)	26.5 (5.6)
Year of first prescription[2]	2010	2010	2010	2009	2010	2009
Days of history[1]	3,573.8 (2181.2)	3,450.3 (2327.4)	3,563.3 (2191.5)	3,052.9 (1907.1)	3,164.8 (1986.2)	3,088.9 (1933.6)
Comorbidity ever (Charlson Index)	354 (86.6%)	28 (73.7%)	382 (85.5%)	59,489 (37.3%)	24,017 (31.8%)	83,506 (35.6%)
Alcohol misuse ever	104 (25.4%)	11 (29.0%)	115 (25.7%)	13,890 (8.7%)	4,759 (6.3%)	18,649 (7.9%)
Self-harm ever	67 (16.4%)	9 (23.7%)	76 (17.0%)	17,232 (10.8)	6,652 (8.8%)	23,884 (10.2%)
Ever anxiety and stress related disorders	151 (36.9%)	16 (42.1%)	167 (37.4%)	44,381 (27.9%)	17,377 (23.0%)	61,758 (26.3%)
Other behavioural/neurologic disorder ever	30 (7.3%)	6 (15.8%)	36 (8.1%)	8,693 (5.5%)	2,956 (3.9%)	11,649 (5.0%)
Ever depression	217 (53.1%)	26 (68.4%)	243 (54.4%)	65,343 (41.0%)	26,097 (34.6%)	91,440 (38.9%)
Ever antidepressants	273 (66.7%)	28 (73.7%)	301 (67.3%)	79.584 (50.0%)	32,230 (42.7%)	111,814 (47.6%)
Ever antipsychotics	175 (42.8%)	13 (34.2%)	188 (42.1%)	28,972 (18.2%)	9,792 (13.0%)	38,764 (16.5%)
Ever hypnotics/anxiolytics	238 (58.2%)	20 (52.6%)	258 (57.7%)	60,092 (37.7%)	25,134 (33.3%)	85,226 (36.3%)

1 Data presented are mean and standard deviation. 2 Data presented are median. †Missing data: BMI data was missing for 14.1% (N= 33,059); IMD data was missing for 43.6% (N= 102,657).

For peer review only - http://bmjopen.bmj.com/site/about/guidelines.xhtml

Figure 1. Percentage (%) of primary care patients with an electronic medical record indicating smoking, from 2007 to 2015, in people with or without dementia

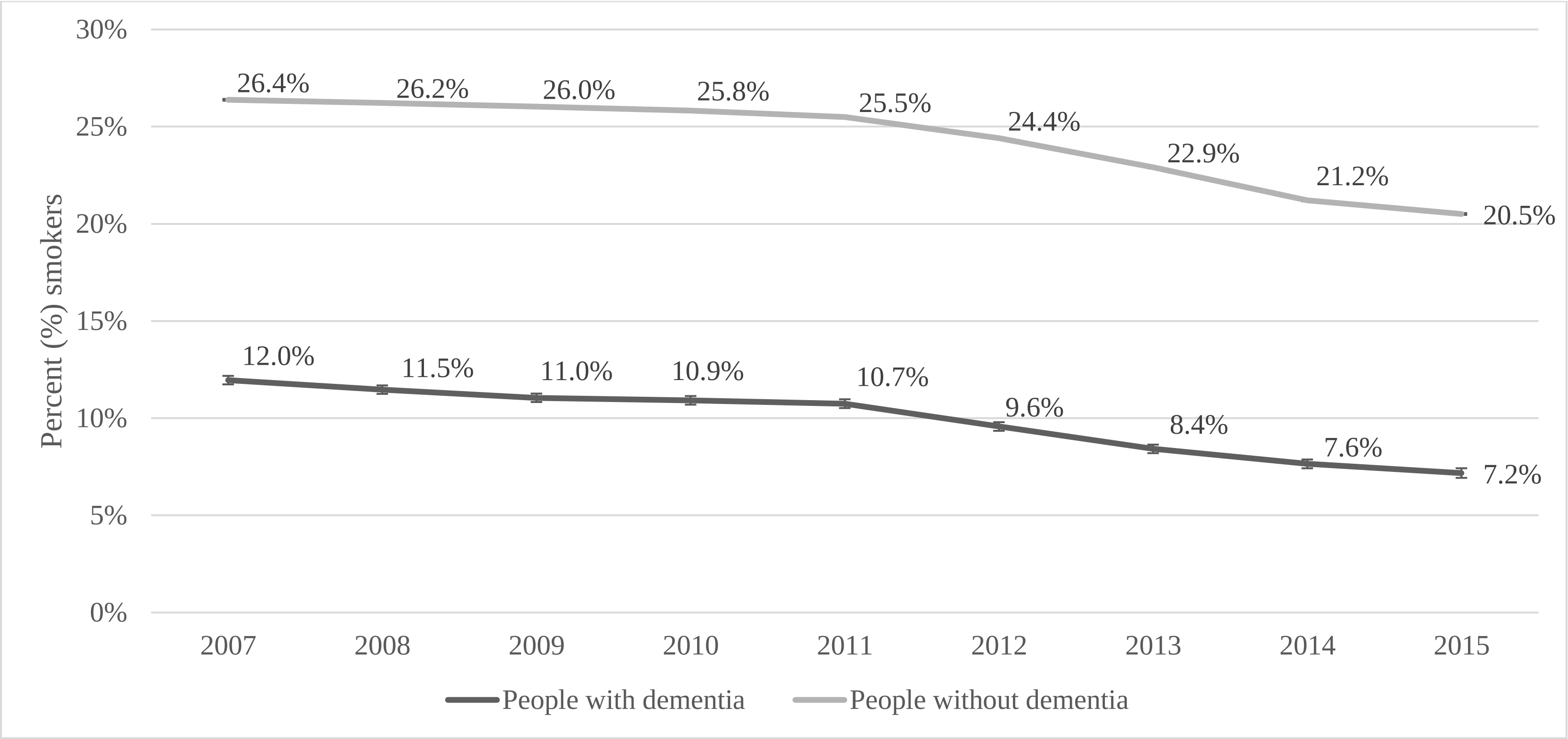

For peer review only - http://bmjopen.bmj.com/site/about/guidelines.xhtml

Figure 2. Prescription prevalence of varenicline or NRT in primary care, from 2007 to 2015, in smokers with dementia, compared to smokers without dementia

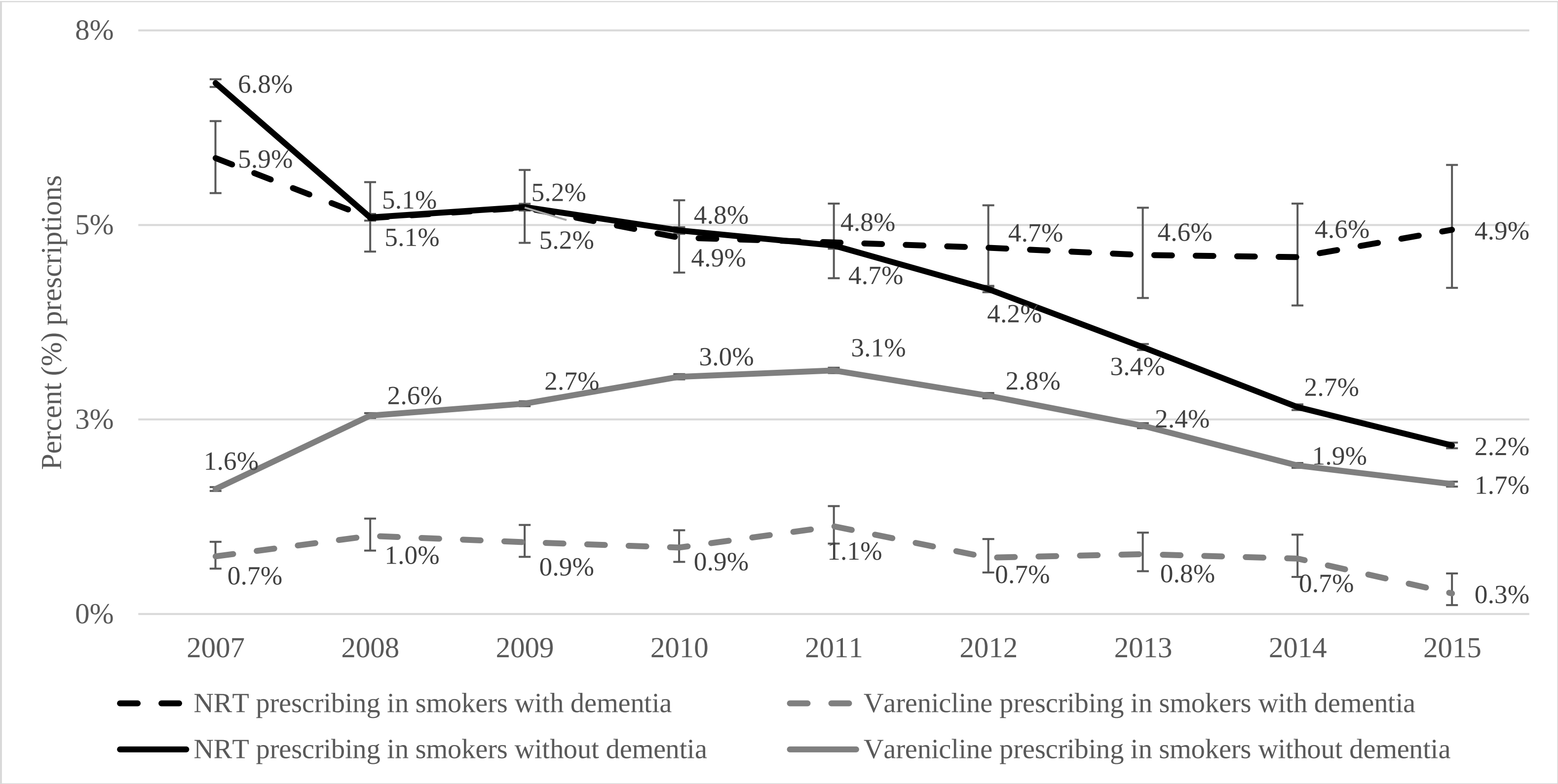

Figure 3. Percentage (%) of people with an electronic medical record indicating smoking cessation at up to 2-years follow-up, in people with and without dementia

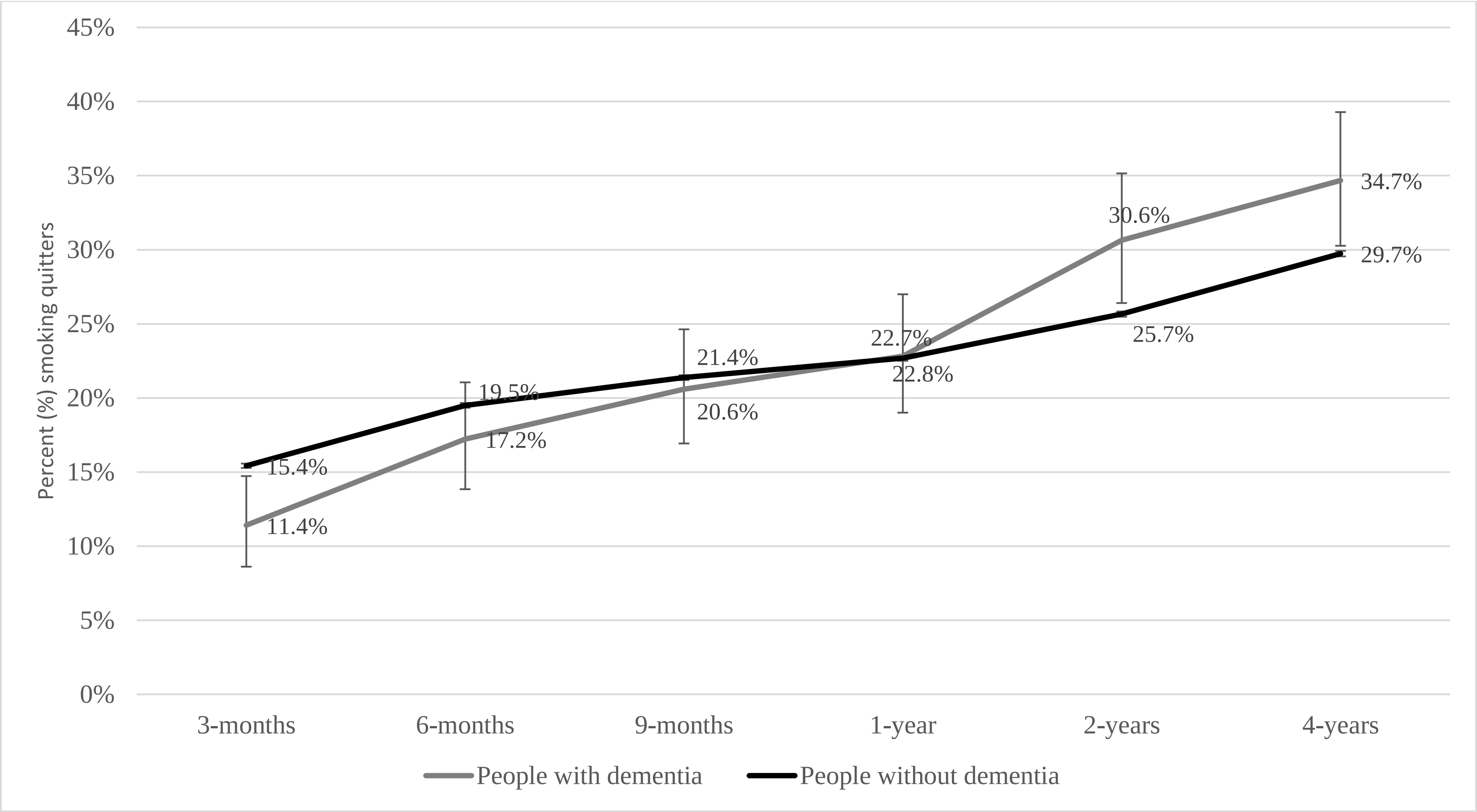

1

eFigure 1. Flow chart of eligible study participants as per protocol restrictions

Assessed for eligibility
N Records=2,877,296
N Patients=566,936

Excluded from analysis due to protocol restrictions (Davies et al. 2015)

	Prescriptions	Patients
Prescriptions issued to patients under the age of 15	6580	1944
Prescription issued after patients' registration period ended	0	0
Prescription issued before patients' registration period began	296490	53529
Prescription issued to patient aged 16 or 17	13154	4425
Prescription issued before 1st September 2006	822244	145382
Both varenicline and NRT were prescribed on the same day	8289	264
Smoking cessation medication was not prescribed by a general practitioner (GP)	141810	16168
Smoking cessation medication prescription had less than one year of historical follow-up data prior to prescription	163821	41112
Bupropion prescriptions	32997	6981
Prescriptions issued to patients who received a smoking cessation medication issued in the previous 18 months	1069188	30278
Patient had previously received an eligible smoking cessation medication prescription	55870	0
Prescribing GP had seen less than 10 patients.	18494	18494
Patients had less than 180 days of follow-up after 1st prescription.	13045	13045
Total excluded N=	2,641,982	331,622

Included in analysis
N patients=235,314

People with dementia
N patients=447

People without dementia
N patients=234,867

For peer review only

eFigure 2. Age and sex standardised percentage (%) of primary care patients with an electronic medical record indicating smoking, from 2007 to 2015, in people with or without dementia

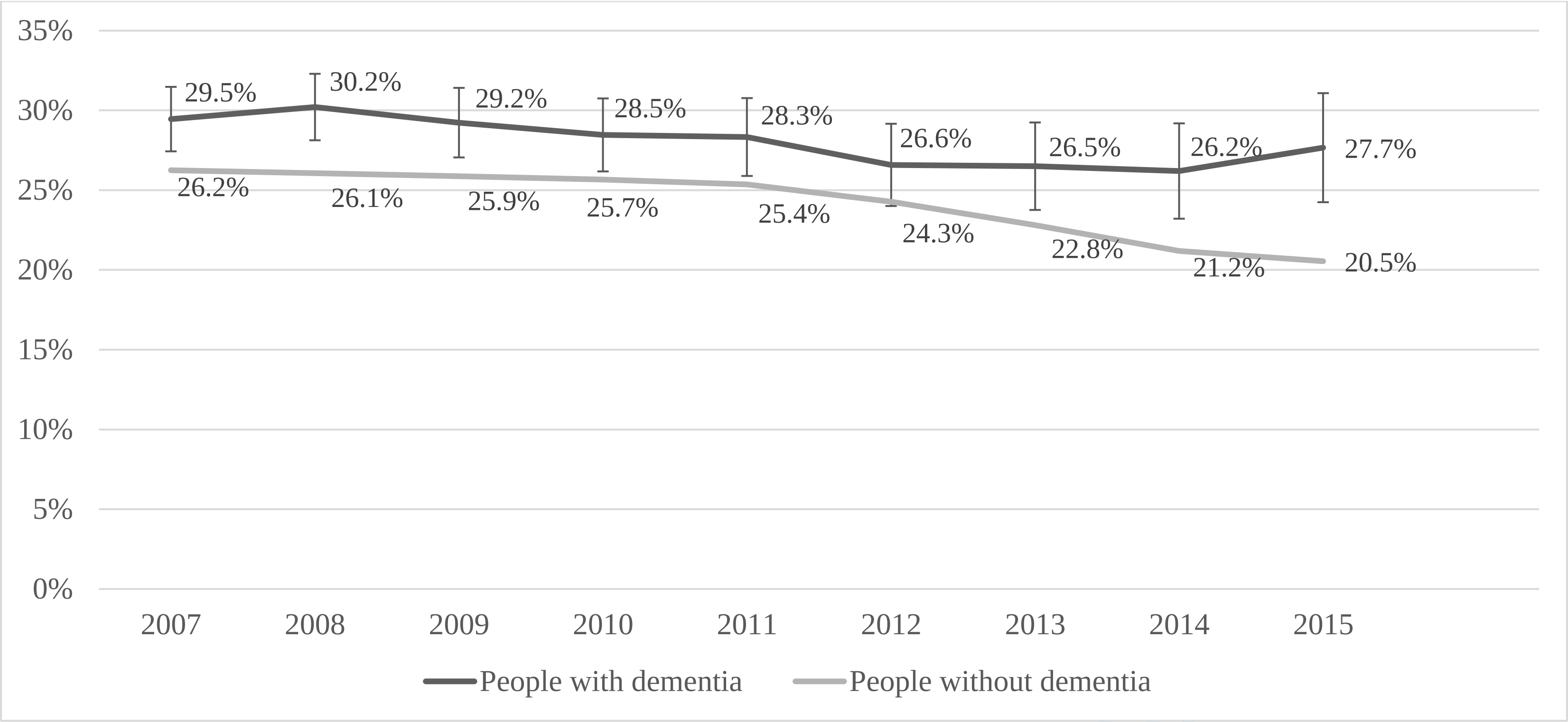

Note on age and sex standardisation

Smoking prevalence rates were directly age- and sex- standardised to account for differences in age and sex between groups with and without dementia. We used the CPRD population in 2015 as our standard population for calculating standardised smoking rates. Age was grouped into three categories (18-49, 50-59, 60+ years).

For peer review only - http://bmjopen.bmj.com/site/about/guidelines.xhtml

eTable 1. Distributions of imputed characteristics in the imputation datasets

Characteristic	People with dementia (N=447)							People without dementia (N=234,867)						
	NRT (N=409)		Varenicline (N=38)		Total		% of data imputed	NRT (N=159,327)		Varenicline (N=75,540)		Total		% of data imputed
	Mean	Standard error	Mean	Standard error	Mean	Standard error		Mean	Standard error	Mean	Standard error	Mean	Standard error	
Body mass index	24.6	0.28	26.6	1.02	24.7	0.27	10.3	26.4	0.02	26.5	0.02	26.4	0.01	14.1
Index of multiple deprivation	3.3	0.08	3.3	0.3	3.3	0.09	53.2	3.3	0.004	3.2	0.007	3.3	0.003	43.6

For peer review only - http://bmjopen.bmj.com/site/about/guidelines.xhtml

eTable 2. Raw numbers for smoking prevalence calculations amongst people with and without dementia, 2007-2015

**Year**	2007	2008	2009	2010	2011	2012	2013	2014	2015
People with dementia									
Numerator (number of smokers with dementia)	10,121	9,548	8,883	8,415	7,807	6,478	5,200	4,077	3,018
Denominator (number people with dementia)	84,647	83,265	80,419	77,085	72,662	67,692	61,771	53,326	42,075
People without dementia									
Numerator (number of smokers without dementia)	1,003,374	1,007,563	1,012,508	1,010,618	983,064	927,818	864,445	747,040	628,116
Denominator (number of people without dementia)	3,802,954	3,841,941	3,888,309	3,912,413	3,855,853	3,801,122	3,773,753	3,523,296	3,062,917

For peer review only

For peer review only - http://bmjopen.bmj.com/site/about/guidelines.xhtml

eTable 3. The likelihood of smokers with ever dementia diagnosis being prescribed varenicline versus NRT, as compared to smokers with no ever dementia diagnosis, N=235,314

	Partially adjusted odds ratio (95% confidence interval) †	Fully adjusted odds ratio (95% confidence interval) ††
Ever dementia diagnosis	0.21 (0.15 to 0.29)	0.26 (0.18 to 0.36)

‡Partially adjusted models were adjusted for: age, sex, year of first prescription.
†† Fully adjusted models were adjusted for: age, sex, days in history, IMD, number of GP visits 1-year prior to first prescription, BMI, year of first prescription, history of major physical morbidity (Charlson Index), alcohol misuse ever, drug misuse ever, depression ever, neurotic disorder ever, self-harm ever, antidepressant prescription ever, antipsychotic prescription ever, hypnotics/anxiolytics prescription ever, other psychotropic medication ever, and other behavioral/neurologic disorder ever. Missing BMI and IMD values were imputed using multiple imputation.
Models were estimated using cluster robust standard errors to account for potential clustering of patients between practices.

For peer review only

eTable 4. Number and percentage (%) of people with an electronic medical record indicating smoking cessation at 3, 6 and 9-months, and 1, 2, and 4-years follow-up, in people with and without dementia

	3-months	6-months	9-months	1-year	2-years	4-years
People without dementia N=234867	36223 (15.4%)	45796 (19.5%)	50193 (21.4%)	53263 (22.7%)	60257 (25.7%)	69842 (29.7%)
People with dementia N=447	51 (11.4%)	77 (17.2%)	92 (20.6%)	102 (22.8%)	137 (30.6%)	155 (34.7%)

For peer review only - http://bmjopen.bmj.com/site/about/guidelines.xhtml

eTable 5. Fully adjusted odds ratios and 95% confidence intervals for the association between the diagnosis of dementia and smoking cessation at 3, 6 and 9-months and 1, 2, and 4-years after prescription

Fully adjusted odds ratio (95% confidence interval) †						
	3-months	6-months	9-months	1-year	2-years	4-years
(N=235,314)	0.56 (0.41 to 0.75)	0.64 (0.49 to 0.83)	0.71 (0.56 to 0.90)	0.75 (0.60 to 0.94)	1.0 (0.81 to 1.23)	1.04 (0.85 to 1.26)

† Fully adjusted models were adjusted for: type of prescription, age, sex, days in history, IMD, number of GP visits 1-year prior to first prescription, BMI, year of first prescription, history of major physical morbidity (Charlson Index), alcohol misuse ever, drug misuse ever, depression ever, neurotic disorder ever, self-harm ever, antidepressant prescription ever, antipsychotic prescription ever, hypnotics/anxiolytics prescription ever, other psychotropic medication ever, and other behavioral/neurologic disorder ever. Missing BMI and IMD values were imputed using multiple imputation.

For peer review only

eTable 6. List of dementia medications codes used in study cohort

Prodcode	Product name
39363	Ebixa 20mg tablets (Lundbeck Ltd)
58937	Exelon 13.3mg/24hours transdermal patches (Novartis Pharmaceuticals UK Ltd)
6225	Memantine 10mg tablets
11751	Rivastigmine 3mg capsules
7329	Galantamine 20mg/5ml oral solution sugar free
60723	Rivastigmine 6mg capsules (Waymade Healthcare Plc)
58780	Voleze 9.5mg/24hours transdermal patches (Focus Pharmaceuticals Ltd)
39362	Ebixa tablets treatment initiation pack (Lundbeck Ltd)
56631	Rivastigmine 13.3mg/24hours transdermal patches
37132	Rivastigmine 9.5mg/24hours transdermal patches
56771	Rivastigmine 3mg capsules (Dr Reddy's Laboratories (UK) Ltd)
20404	Exelon 4.5mg capsules (Novartis Pharmaceuticals UK Ltd)
57171	Erastig 9.5mg/24hours transdermal patches (Teva UK Ltd)
61676	Donepezil 1mg/ml oral solution sugar free
24088	Reminyl XL 24mg capsules (Shire Pharmaceuticals Ltd)
11635	Galantamine 12mg tablets
60192	Galantex XL 8mg capsules (Creo Pharma Ltd)
57627	Erastig 4.6mg/24hours transdermal patches (Teva UK Ltd)
11654	Galantamine 8mg tablets
2930	Donepezil 5mg tablets
5616	Exelon 6mg capsules (Novartis Pharmaceuticals UK Ltd)
58969	Rivastigmine 4.6mg/24hours transdermal patches (A A H Pharmaceuticals Ltd)
48482	Galsya XL 8mg capsules (Consilient Health Ltd)
48442	Donepezil 5mg orodispersible tablets
55928	Exelon 4.5mg capsules (Waymade Healthcare Plc)
53882	Rivastigmine 2mg/ml oral solution
58709	Donepezil 10mg tablets (A A H Pharmaceuticals Ltd)
59871	Donepezil 10mg/5ml oral suspension

For peer review only

For peer review only - http://bmjopen.bmj.com/site/about/guidelines.xhtml

55720	Gatalin XL 24mg capsules (Aspire Pharma Ltd)
7361	Galantamine 24mg modified-release capsules
61476	Acumor XL 24mg capsules (Generics (UK) Ltd)
57139	Ebixa 10mg tablets (DE Pharmaceuticals)
62164	Alzest 9.5mg/24hours transdermal patches (Dr Reddy's Laboratories (UK) Ltd)
53922	Donepezil 10mg orodispersible tablets (Consilient Health Ltd)
36848	Aricept Evess 5mg orodispersible tablets (Eisai Ltd)
60493	Galantex XL 24mg capsules (Creo Pharma Ltd)
29288	Reminyl 4mg/ml oral solution (Shire Pharmaceuticals Ltd)
9966	Ebixa 5mg/pump actuation oral solution (Lundbeck Ltd)
58947	Donepezil 10mg tablets (Accord Healthcare Ltd)
5247	Aricept 10mg tablets (Eisai Ltd)
11716	Exelon 3mg capsules (Novartis Pharmaceuticals UK Ltd)
61920	Luventa XL 8mg capsules (Fontus Health Ltd)
10187	Galantamine 4mg tablets
37444	Exelon 4.6mg/24hours transdermal patches (Novartis Pharmaceuticals UK Ltd)
18587	Reminyl XL 8mg capsules (Shire Pharmaceuticals Ltd)
56421	Gatalin XL 8mg capsules (Aspire Pharma Ltd)
10255	Galantamine 8mg modified-release capsules
4597	Rivastigmine 1.5mg capsules
9854	Reminyl 4mg tablets (Shire Pharmaceuticals Ltd)
61385	Nemdatine 10mg tablets (Actavis UK Ltd)
11546	Exelon 1.5mg capsules (Novartis Pharmaceuticals UK Ltd)
14309	Galantamine 16mg modified-release capsules
37188	Aricept Evess 10mg orodispersible tablets (Eisai Ltd)
5334	Reminyl 12mg tablets (Shire Pharmaceuticals Ltd)
56709	Gatalin XL 16mg capsules (Aspire Pharma Ltd)
2931	Donepezil 10mg tablets
61921	Luventa XL 24mg capsules (Fontus Health Ltd)
11827	Rivastigmine 2mg/ml oral solution sugar free
37957	Exelon 9.5mg/24hours transdermal patches (Novartis Pharmaceuticals UK Ltd)

For peer review only

48015	Galsya XL 24mg capsules (Consilient Health Ltd)
53842	Aricept 5mg tablets (Waymade Healthcare Plc)
18800	Ebixa 10mg tablets (Lundbeck Ltd)
59993	Galantex XL 16mg capsules (Creo Pharma Ltd)
36976	Rivastigmine 4.6mg/24hours transdermal patches
56600	Donepezil 5mg tablets (Zentiva)
35088	Donepezil 10mg orodispersible tablets sugar free
5400	Aricept 5mg tablets (Eisai Ltd)
59330	Voleze 4.6mg/24hours transdermal patches (Focus Pharmaceuticals Ltd)
18062	Reminyl 8mg tablets (Shire Pharmaceuticals Ltd)
11752	Rivastigmine 4.5mg capsules
38976	Memantine 5mg+10mg+15mg+20mg Tablet
35179	Donepezil 5mg orodispersible tablets sugar free
60107	Donepezil 5mg tablets (Alliance Healthcare (Distribution) Ltd)
20140	Reminyl XL 16mg capsules (Shire Pharmaceuticals Ltd)
39240	Memantine 20mg tablets
18556	Exelon 2mg/ml oral solution (Novartis Pharmaceuticals UK Ltd)
9786	Rivastigmine 6mg capsules
11837	Memantine 10mg/ml oral solution sugar free
48443	Donepezil 10mg orodispersible tablets
61618	Nemdatine 20mg tablets (Actavis UK Ltd)
12843	Ginkyo 120mg tablets (Ceuta Healthcare Ltd)
30120	Ginkyo 50mg tablets (Ceuta Healthcare Ltd)
61128	HealthAid Ginko Vital (Biloba) 5g capsules (HealthAid Ltd)

For peer review only - http://bmjopen.bmj.com/site/about/guidelines.xhtml

eTable 7. List of dementia diagnoses codes used in study cohort

Medcode	Readterm
26270	[X]Lewy body dementia
44674	Senile dementia with depressive or paranoid features
19393	[X]Vascular dementia, unspecified
4693	[X] Unspecified dementia
25704	[X]Presenile dementia,Alzheimer's type
19477	Arteriosclerotic dementia
55313	[X]Other vascular dementia
30032	Presenile dementia with paranoia
18386	Senile dementia with paranoia
56912	Arteriosclerotic dementia with delirium
33707	Senile and presenile organic psychotic conditions
4357	[X] Senile dementia NOS
12710	Dementia annual review
41089	Senile dementia with depressive or paranoid features NOS
9509	[X]Dementia in Parkinson's disease
15165	Presenile dementia
49513	Presenile dementia with delirium
42602	Uncomplicated presenile dementia
7572	Lewy body disease
30706	[X]Dementia in Alzheimer's dis, atypical or mixed type
1916	Senile dementia
55467	Arteriosclerotic dementia with paranoia
9565	[X]Arteriosclerotic dementia
8934	[X]Subcortical vascular dementia
31016	[X]Mixed cortical and subcortical vascular dementia
43089	Uncomplicated arteriosclerotic dementia
11175	[X]Multi-infarct dementia
42279	Arteriosclerotic dementia NOS

For peer review only - http://bmjopen.bmj.com/site/about/guidelines.xhtml

29386	[X]Dementia in Alzheimer's disease, unspecified
38438	Presenile dementia NOS
8634	Multi infarct dementia
1917	Alzheimer's disease
61528	[X]Alzheimer's disease type 2
1350	Senile/presenile dementia
38678	[X]Dementia in Alzheimer's disease with late onset
27677	Presenile dementia with depression
46762	[X]Alzheimer's disease type 1
11379	[X]Senile dementia,Alzheimer's type
21887	Senile dementia with depression
2882	Senile or presenile psychoses NOS
25386	Dementia in conditions EC
49263	[X]Dementia in Alzheimer's disease with early onset
59122	[X]Other Alzheimer's disease
34944	[X] Primary degenerative dementia NOS
40805	Excepted from dementia quality indicators: Informed dissent
37015	Senile dementia with delirium
6578	[X]Vascular dementia
7323	Uncomplicated senile dementia
55838	[X]Predominantly cortical dementia
29512	Senile degeneration of brain
12621	[X]Dementia in other diseases classified elsewhere
7664	[X]Dementia in Alzheimer's disease
60059	[X]Primary degen dementia, Alzheimer's type, presenile onset
15249	Other senile and presenile organic psychoses
43346	[X]Primary degen dementia of Alzheimer's type, senile onset
53446	[X]Delirium superimposed on dementia
43292	Arteriosclerotic dementia with depression
32057	Alzheimer's disease with late onset
8195	[X]Alzheimer's dementia unspec

For peer review only - http://bmjopen.bmj.com/site/about/guidelines.xhtml

64267	[X]Dementia in other specified diseases classif elsewhere
46488	[X]Vascular dementia of acute onset
27759	[X] Senile dementia, depressed or paranoid type
16797	Alzheimer's disease with early onset
48501	[X] Presenile dementia NOS
109047	Antipsychotic drug therapy for dementia
28402	[X]Dementia in Pick's disease
54106	[X]Dementia in Creutzfeldt-Jakob disease
37014	[X]Dementia in Huntington's disease
41185	[X]Dementia in human immunodef virus [HIV] disease
11136	Pick's disease
62132	Drug-induced dementia
2731	Cerebral atrophy
54744	Cerebral degeneration due to cerebrovascular disease

For peer review only

STROBE Statement—Checklist of items that should be included in reports of ***cohort studies***

	Item No	Recommendation	Page
**Title and abstract**	1	(*a*) Indicate the study's design with a commonly used term in the title or the abstract	P3L43
		(*b*) Provide in the abstract an informative and balanced summary of what was done and what was found	P4L69
**Introduction**			
Background/rationale	2	Explain the scientific background and rationale for the investigation being reported	P5L92
Objectives	3	State specific objectives, including any prespecified hypotheses	P6L112
**Methods**			
Study design	4	Present key elements of study design early in the paper	P7L120
Setting	5	Describe the setting, locations, and relevant dates, including periods of recruitment, exposure, follow-up, and data collection	P7L136
Participants	6	(*a*) Give the eligibility criteria, and the sources and methods of selection of participants. Describe methods of follow-up	P7L136
		(*b*) For matched studies, give matching criteria and number of exposed and unexposed	NA
Variables	7	Clearly define all outcomes, exposures, predictors, potential confounders, and effect modifiers. Give diagnostic criteria, if applicable	P8-10L154-185
Data sources/ measurement	8*	For each variable of interest, give sources of data and details of methods of assessment (measurement). Describe comparability of assessment methods if there is more than one group	P7-8L136-152
Bias	9	Describe any efforts to address potential sources of bias	P11L192-195
Study size	10	Explain how the study size was arrived at	Supplementary eFigure 1
Quantitative variables	11	Explain how quantitative variables were handled in the analyses. If applicable, describe which groupings were chosen and why	P11L201-P12L218
Statistical methods	12	(*a*) Describe all statistical methods, including those used to control for confounding	P11L201-P12L218
		(*b*) Describe any methods used to examine subgroups and interactions	NA
		(*c*) Explain how missing data were addressed	P10L181-183, P11L192-195
		(*d*) If applicable, explain how loss to follow-up was addressed	NA
		(*e*) Describe any sensitivity analyses	NA
**Results**			
Participants	13*	(a) Report numbers of individuals at each stage of study—eg numbers potentially eligible, examined for eligibility, confirmed eligible, included in the study, completing follow-up, and analysed	Supplementary eFigure 1
		(b) Give reasons for non-participation at each stage	Supplementary eFigure 1
		(c) Consider use of a flow diagram	Supplementary eFigure 1
Descriptive data	14*	(a) Give characteristics of study participants (eg demographic,	Table 1

For peer review only - http://bmjopen.bmj.com/site/about/guidelines.xhtml

		clinical, social) and information on exposures and potential confounders	
		(b) Indicate number of participants with missing data for each variable of interest	Table 1
		(c) Summarise follow-up time (eg, average and total amount)	Table 1
Outcome data	15*	Report numbers of outcome events or summary measures over time	Supplementary eTables 2 and 4
Main results	16	(*a*) Give unadjusted estimates and, if applicable, confounder-adjusted estimates and their precision (eg, 95% confidence interval). Make clear which confounders were adjusted for and why they were included	Supplementary eTables 3 and 5
		(*b*) Report category boundaries when continuous variables were categorized	NA
		(*c*) If relevant, consider translating estimates of relative risk into absolute risk for a meaningful time period	NA
Other analyses	17	Report other analyses done—eg analyses of subgroups and interactions, and sensitivity analyses	NA
**Discussion**			
Key results	18	Summarise key results with reference to study objectives	P17L319-322
Limitations	19	Discuss limitations of the study, taking into account sources of potential bias or imprecision. Discuss both direction and magnitude of any potential bias	P15L264-276
Interpretation	20	Give a cautious overall interpretation of results considering objectives, limitations, multiplicity of analyses, results from similar studies, and other relevant evidence	P17L319-322
Generalisability	21	Discuss the generalisability (external validity) of the study results	P16L284-287
**Other information**			
Funding	22	Give the source of funding and the role of the funders for the present study and, if applicable, for the original study on which the present article is based	P18L324-335

*Give information separately for exposed and unexposed groups.

Note: An Explanation and Elaboration article discusses each checklist item and gives methodological background and published examples of transparent reporting. The STROBE checklist is best used in conjunction with this article (freely available on the Web sites of PLoS Medicine at http://www.plosmedicine.org/, Annals of Internal Medicine at http://www.annals.org/, and Epidemiology at http://www.epidem.com/). Information on the STROBE Initiative is available at http://www.strobe-statement.org.

For peer review only

Use of varenicline and nicotine replacement therapy in people with and without general practitioner-recorded dementia: retrospective cohort study of routine electronic medical records.

Journal:	*BMJ Open*
Manuscript ID	bmjopen-2018-027569.R2
Article Type:	Research
Date Submitted by the Author:	24-May-2019
Complete List of Authors:	Itani, Taha; School of Psychological Science,<br>Martin, Richard; University of Bristol, Social Medicine<br>Rai, Dheeraj; University of Bristol, Centre for Academic Mental Health, School of Social & Community Medicine<br>Jones, Tim; NIHR CLAHRC West,<br>Taylor, Gemma; University of Birmingham, Department of Primary Care Clinical Sciences<br>Thomas, Kyla; University of Bristol, School of Social and Community Medicine<br>Munafo, Marcus; University of Bristol, Experimental Psychology<br>Davies, Neil; University of Bristol,<br>Taylor, Amy; 5. School of Experimental Psychology University of Bristol,
**Primary Subject Heading**:	Smoking and tobacco
Secondary Subject Heading:	Epidemiology
Keywords:	Dementia < NEUROLOGY, Smoking cessation, Smoking prevalence

PAPER TITLE

Use of varenicline and nicotine replacement therapy in people with and without general practitioner-recorded dementia: retrospective cohort study of routine electronic medical records.

Authors

Taha Itani PhD[1,2]; Richard M Martin PhD [1,3,4]; Dheeraj Rai PhD[3,5,6], Tim Jones PhD[7]; Gemma MJ Taylor PhD[8] Kyla H Thomas PhD[9]; Marcus R Munafò PhD[1,2]; Neil M Davies PhD[1,4] and Amy E Taylor PhD[3,4]

Author affiliations

1 Medical Research Council Integrative Epidemiology Unit at the University of Bristol, BS8 2BN, United Kingdom.

2 UK Centre for Tobacco and Alcohol Studies, School of Psychological Science, University of Bristol, 12a Priory Road, Bristol, BS8 1TU, United Kingdom.

3 NIHR Biomedical Research Centre at the University Hospitals Bristol NHS Foundation Trust and the University of Bristol, United Kingdom.

4 Population Health Sciences, Bristol Medical School, University of Bristol, Oakfield Grove, Bristol, BS8 2BN, United Kingdom.

5 Centre for Academic Mental Health; Bristol Medical School, University of Bristol, Barley House, Oakfield Grove, Bristol, BS8 2BN, United Kingdom.

6 Avon & Wiltshire Partnership NHS Mental Health Trust, Bristol, UK

7 National Institute for Health Research Collaboration for Leadership in Applied Health Research and Care West (NIHR CLAHRC West) at University Hospitals Bristol NHS Foundation Trust, 9th Floor Whitefriars, Lewins Mead, Bristol, BS1 2NT, United Kingdom.

8 Addiction and Mental Health Group (AIM) Department of Psychology, University of Bath, Claverton Down, Bath, BA2 7AY.

9 Population Health Sciences, Bristol Medical School, University of Bristol, Canynge Hall, Bristol, BS8 2PS, United Kingdom

For peer review only - http://bmjopen.bmj.com/site/about/guidelines.xhtml

Author Positions

Taha Itani, Research Associate in Epidemiology; Richard M Martin, Professor of Clinical Epidemiology; Dheeraj Rai, Consultant Senior Lecturer in Psychiatry; Tim Jones, Senior Research Associate; Gemma MJ Taylor, Lecturer in Clinical Psychology; Kyla H Thomas, Consultant Senior Lecturer in Public Health Medicine and NIHR Postdoctoral Fellow; Marcus R Munafò, Professor of Biological Psychology; Neil M Davies, Senior Research Fellow and Amy E Taylor, Research Fellow.

Correspondence

Dr Taha Itani, ti17926@bristol.ac.uk

Word count

3,125

review only

For peer review only - http://bmjopen.bmj.com/site/about/guidelines.xhtml

ABSTRACT

Objectives: Our primary objective was to estimate smoking prevalence and prescribing rates of varenicline and NRT in people with and without GP-recorded dementia. Our secondary objective was to assess and compare quit rates of smokers with versus without general practitioner (GP)-recorded dementia who were prescribed varenicline or nicotine replacement therapy (NRT) for smoking cessation.

Design: A retrospective cohort study based on the analysis of electronic medical records within the Clinical Practice Research Datalink (2007-2015).

Setting: 683 general practices in England.

Participants: People with and without GP-recorded dementia, aged 18 years and have a code indicating that they are a current smoker.

Intervention: Index prescription of varenicline or NRT (from 1st September 2006).

Outcome measures: The primary outcomes were smoking prevalence and prescribing rates of varenicline and NRT (2007-2015). The secondary outcome was smoking cessation at 2 years.

Results: Age and sex-standardised prevalence of smoking was slightly higher in people with GP-recorded dementia than in those without. There were 235,314 people aged 18 years and above prescribed NRT or varenicline. Amongst smokers with GP-recorded dementia (N=447), 409 were prescribed NRT and 38 varenicline. Smokers with GP-recorded dementia were 74% less likely (95% confidence interval: 64% to 82%) to be prescribed varenicline than NRT, compared to smokers without GP-recorded dementia. Compared to people without GP-recorded dementia, people with GP-recorded dementia had consistently lower prescribing rates of varenicline from 2007 to 2015.

Two years after prescription, there was no clear evidence for a difference in the likelihood of smoking cessation after prescription of these medications between individuals with and without dementia (OR 1.0, 95% CI: 0.8, 1.2).

For peer review only

Conclusions: Between 2007 and 2015, people with GP-recorded dementia were less likely to be prescribed varenicline than those without dementia. Quit rates following prescription of either NRT or varenicline were similar in those with and without dementia.

Strengths and limitations of this study

- This study used primary care data from the Clinical Practice Research Datalink (CPRD) which are representative of the UK primary care population.
- Expert-reviewed codelists were developed to define both the exposure and the outcome which would reduce misclassification bias.
- Due to the small sample size of people with GP-recorded dementia, we were not able to test the relative effectiveness of varenicline versus NRT on smoking cessation using regression models.
- Data on smokers who purchase over-the-counter NRT were not available, and therefore the prevalence of NRT might be underestimated.

For peer review only

INTRODUCTION

Smoking is a leading cause of mortality and morbidity worldwide. About 12% of global deaths were linked to smoking in 2015.[1] There is substantial evidence that smoking is associated with an increased risk of developing dementia.[2 3] For instance, it is estimated that 14% of Alzheimer's disease (AD) cases worldwide are attributable to smoking.[4] Smoking is thought to accelerate the onset of dementia mainly via vascular risk factors such as narrowing of blood vessels in the heart and the brain, thereby triggering oxidative stress.[4 5]

Few studies report smoking prevalence among people with dementia. In a cross-sectional analysis of patients treated for AD in a neurology clinic during a 2-year period, past smoking prevalence was 29% (N=21/72).[6] In a case-control study of patients with vascular dementia, the rate of current tobacco use was 9% (N=17/198) as compared to 6% (N=11/199) in the control group.[7] Beyond the harmful health effects of smoking in people with dementia, there are concerns that smokers in this group may have a higher likelihood of fire accidents due to their compromised cognitive state.[8]

Since there are currently no available treatments to cure dementia, there is a growing interest in identifying modifiable risk factors for reducing the occurrence of the disease, to delay dementia onset, and reduce its burden.[4 9] Smoking cessation could potentially decrease the risk or slow the development of dementia[10] and could improve the quality of life of older adults through improved physical, and mental wellbeing.[9 11] Little is known about whether people with dementia are prescribed smoking cessation agents and whether they are effective in this group.

Based on a Cochrane review of 136 trials, it was reported that NRT (compared to placebo or no treatment) can help people who make a quit attempt to increase their chances of successfully

For peer review only - http://bmjopen.bmj.com/site/about/guidelines.xhtml

stopping smoking (Hartmann-Boyce et al., 2018). Data from observational studies [12 13] and meta-analyses of randomized controlled trials[14] indicate that varenicline is more effective than single form nicotine replacement therapy (NRT) for smoking cessation in the general population. However, it is unclear whether varenicline or NRT could help smokers with dementia to quit smoking.

Therefore in this study we aimed to: 1) describe the rates of smoking prevalence and smoking cessation medication prescribing amongst people with and without GP-recorded dementia in UK primary care settings from 2007 to 2015; and; 2) assess and compare associations of varenicline and NRT on smoking cessation in people with GP-recorded dementia, compared to those without, at 3, 6, 9 months and 1, 2, 4 years after first prescription.

For peer review only - http://bmjopen.bmj.com/site/about/guidelines.xhtml

METHODS

Data source and population

We conducted a retrospective cohort study using electronic medical records from 683 general practices in England from 2007 to 2015 using data from the Clinical Practice Research Database (CPRD). Patient data from the CPRD are broadly representative of the UK primary care population in terms of age, sex and ethnicity.[15] These data have been validated, audited, and quality checked.[16] The study's protocol (15_115R) was approved by the Independent Scientific Advisory Committee for MHRA Database Research (https://www.cprd.com/isac/).

Code lists

We defined variables using medical and product codes within the CPRD. All code lists were developed using a list from a previously published study.[12] These codelists were derived from the the British National Formulary (BNF) and the International Classification of Diseases (ICD-10) and then agreed upon by field experts (DR, KHT). A previous systematic review that checked the validity of coding of various diagnoses in what was then the General Practice Research Database (now CPRD) suggests that coding for dementia and Alzheimer's is relatively accurate.[17]

Study subjects

During the study period (2007 to 2015), we included people (aged ≥18 years) with information about their smoking status (either smokers or non-smokers) for smoking prevalence estimates, and we included smokers prescribed either varenicline or NRT for prescribing prevalence and for

For peer review only - http://bmjopen.bmj.com/site/about/guidelines.xhtml

comparing quit rates. We used an open cohort design, with new patients entering the cohort throughout the observation period.

For the primary objective, people were categorised as having dementia if: i) they had ever been diagnosed with dementia (based on ICD-10 diagnoses F00-F03), or ii) if they were prescribed dementia medications: (BNF chapter 4.11) (see supplementary file for a list of all Read and product codes used in this study). Then, the earliest record of GP-recorded dementia in the CPRD was taken forward ensuring that all records used were within the registration period of each patient. Patients with no records of the above-mentioned diagnosis/prescriptions were considered to have no GP-recorded dementia (for clarity, we hence forth refer to this as dementia).

For the secondary objective, we constructed a cohort of eligible first varenicline/NRT prescriptions (see eFigure 1 for a flow chart of numbers of patients excluded and reasons for exclusion). Within that cohort of eligible prescriptions, we considered individuals with dementia to be those with recorded Read codes for ever dementia/dementia medications prior to first varenicline/NRT prescription; we did this to ensure that a diagnosis of dementia preceded the exposure (prescription of a smoking cessation medicine).

Variables

Smoking and prescribing prevalence estimates

For smoking prevalence estimates, a patient's smoking status (aged ≥18 years) was defined by a record indicating smoking/non-smoking or prescription of NRT/varenicline in that year. In case of missing information about smoking, the patient's smoking status was carried forward until

For peer review only - http://bmjopen.bmj.com/site/about/guidelines.xhtml

there was evidence of a change in smoking status or carried backwards if smoking status was only recorded in the final year of registration. Records that were outside the registration period for each patient were excluded.

For peer review only

For peer review only - http://bmjopen.bmj.com/site/about/guidelines.xhtml

Exposure

Exposure was defined as prescription of varenicline or NRT (e.g., patches, etc. on prescription as opposed to over-the-counter, hence forth we refer to this as NRT).

Prescriptions used to define exposure groups were issued between September 1st, 2006 and August 31st, 2016, with no prior record of use of a related product in the preceding 18-months. We used the first treatment episode to ensure that intervention groups were "new users" of the medication.[18] We did not model multiple and repeated prescriptions of smoking cessation medications during follow-up because this is likely to be strongly related to patient characteristics.

Outcome: smoking cessation

Smoking cessation was defined as having an electronic record indicating a non-smoking status. The closest smoking record to each follow-up period was selected to determine each study participant's smoking status; i.e., the most recent smoking record identified between cohort entry and each follow-up period (e.g., 3-months, 6-months). People with missing smoking data (beyond 180 days) were assumed to be continuing smokers[19] which has been previously found to be robust in sensitivity analyses.[12]

For peer review only - http://bmjopen.bmj.com/site/about/guidelines.xhtml

Covariates

Covariates included patients' age at time of prescription, sex, index of multiple deprivation score (IMD), mean number of GP visits one year prior to first prescription, year of first prescription of a smoking cessation medication, body mass index (BMI), days registered in the CPRD, the Charlson Index (a measure of chronic illness),[20] alcohol misuse, history of mental disorder or psychoactive medication prescriptions, evidence of other psychoactive medication prescription or other less common psychiatric disorders. We used multiple imputation to handle missing data on BMI and IMD. This was done using the ICE command in Stata where we produced 20 imputed datasets (eTable 1). We included all exposures, covariates, and outcomes in the imputation model.[21]

Follow-up

The secondary outcome was smoking cessation at 2 years, and this was also assessed at 3, 6, 9 months and 1, and 4 years after first prescription of varenicline or NRT.

Statistical analysis

Smoking prevalence

Smoking rates were calculated by dividing the number of people with dementia who had Read codes indicating current smoking for each year between 2007 and 2015 by the total number of people with dementia and a smoking status code (indicating current or non/ex-smoking) each year between 2007 and 2015. For comparison, smoking prevalence was also estimated amongst people without dementia.

For peer review only - http://bmjopen.bmj.com/site/about/guidelines.xhtml

Prescribing prevalence

The prevalence of varenicline and NRT prescribing amongst current smokers was calculated by dividing the number of prescriptions each year from 2007 to 2015 (there were no varenicline prescriptions for patients with dementia in 2006) by the number of current smokers in each year. Prevalence was estimated for people with and without dementia. Individuals with missing smoking information were excluded from the denominators.

Association of varenicline and NRT prescriptions with smoking cessation

We had originally planned to compare the effectiveness of NRT and varenicline for smoking cessation in individuals with dementia. However, given the small numbers of individuals prescribed varenicline, we had insufficient power to conduct this analysis. Therefore, we compared the effectiveness of being prescribed either varenicline or NRT on smoking cessation in individuals with dementia compared to individuals without dementia. This was determined by estimating quit rates at each follow-up period for individuals prescribed either of these medications. This was calculated by dividing the number of non-smokers in each group by the total number of people in that group at each follow-up period. All analyses were conducted using Stata 14 MP.

Patient and Public Involvement

This study was based on the analysis of anonymised primary care data. No patients were involved during the design and analysis of this study.

For peer review only - http://bmjopen.bmj.com/site/about/guidelines.xhtml

RESULTS

Smoking prevalence and smoking cessation medication prescribing estimates

Unadjusted smoking prevalence amongst people with dementia steadily decreased from 11% (N=2,965/27,432 in 2007 to 7% (N=2,690/36,249) in 2015 (Figure 1). These estimates were consistently lower than in people without dementia, 26% (N= 1,010,530/ 3,860,169) in 2007 and 21% (N=628,444/3,068,743) in 2015 respectively (eTable 2). However, after age and sex standardization, the smoking prevalence amongst people with dementia was slightly higher than in people without (eFigure 2).

The rate of NRT prescribing in people without dementia was 7% (68,935/1,010,530) in 2007 which decreased to 2% (N=13626/628,444) by 2015, whereas NRT prescribing rates amongst people with dementia increased during the same period. Compared to people without dementia, people with dementia had lower prescribing rates of varenicline (Figure 2).

Smoking cessation amongst individuals prescribed NRT and Varenicline

Of the 235,314 people included in this analysis, 447 were people with dementia, whereas 234,867 were people without (eFigure 1). Overall, 159,736 smokers were prescribed NRT and 75,578 prescribed varenicline (Table 1). The mean age of people with dementia at the time of smoking medication prescription was about 72 years (SD=12.6), while that of people without dementia was 46 years (SD=14.8) People with dementia were about 25 years older, had more GP visits 1-year prior to the first prescription, suffered from more comorbidities, and received more mental health-related prescriptions than those without dementia (Table 1).

For peer review only - http://bmjopen.bmj.com/site/about/guidelines.xhtml

Smokers with dementia were 74% (95% confidence interval: 64% to 82%) less likely to be prescribed varenicline than NRT, compared to smokers without dementia (eTable 3). The proportion of people with and without dementia who quit smoking after being prescribed either varenicline or NRT increased throughout the study's follow-up period (3-months to 4- years). At 2 years follow-up, people with dementia were more likely to quit smoking (30.6%, 95% CI: 25.8% to 35.1%) than those without (25.7%, 95% CI: 25.4% to 25.8%) when prescribed either varenicline or NRT (Figure 3) (eTable 4). However, after adjusting for all covariates, we found no evidence for a difference in quit rates between individuals with and without dementia (OR=1.0, 95% CI: 0.81-1.23) (eTable 5).

For peer review only

For peer review only - http://bmjopen.bmj.com/site/about/guidelines.xhtml

DISCUSSION

People with dementia were less likely to be prescribed varenicline compared to those without dementia. There was no clear evidence for a difference in quit rates in individuals with and without dementia following prescription of NRT or varenicline.

A strength of this study is that we used primary care data from the CPRD which are representative of the UK primary care population.[15] Hence, smoking rates in people with dementia in this study are likely to be generalisable to the dementia population in the UK and in similar countries. Additionally, we used expert-reviewed codelists to define both the exposure and the outcome which would reduce misclassification bias (i.e., classifying people with dementia as people without and vice versa).

There are several limitations to this research. Due to the small sample size of people with dementia, we were not able to test the relative effectiveness of varenicline versus NRT on smoking cessation using regression models. We had no data on smokers who purchase over-the-counter NRT, therefore we might be underestimating the prevalence of NRT use, particularly amongst people without dementia. Hence, it is likely that the prevalence of NRT use amongst people without dementia is larger than the prevalence of NRT prescribing in this study. Moreover, outcome definition (smoking vs. non-smoking status) was based on self-reported data rather than biochemical verification of smoking status. Additionally, social desirability bias may occur when unsuccessful quitters don't disclose their smoking status truthfully. We also relied on point estimates (i.e. smoking status reported at a single timepoint) for making conclusions about smoking status. This may not have captured long-term abstinence. In other words, it is possible that smoking status may have fluctuated between GP visits. A further limitation is having no information about patient adherence in taking their prescribed smoking cessation medications.

For peer review only - http://bmjopen.bmj.com/site/about/guidelines.xhtml

We are not aware of previous population-based studies that estimated the smoking rates amongst people with dementia as the available evidence has been limited to small cross-sectional studies. For instance, a community study in China found that about 17% (N=69/186) of the elderly sample with dementia were current smokers compared to 25% (N=415/1664) in people without dementia.[22] Results from the Toyama dementia survey in Japan show that only 4% of people with dementia smoked compared to 10% in those without.[23] This high variability in the results points to the need for larger and more representative studies in people with dementia to be conducted. Meanwhile, we found that smoking prevalence has decreased steadily amongst people without dementia, from 26% in 2007 to 21% in 2015. This was fairly similar to the general population in England as evidenced by results from the Smoking Toolkit Study (24.2% in 2007 to 18.7% in 2015)[24] and therefore speaks to the external validity of our study.

We observed a low prevalence of varenicline prescribing during our study period in people with dementia. Our estimate for individuals without dementia was consistent with findings from a previous study that examined the use of varenicline for smoking cessation treatment in UK primary care using data from THIN database in 2011. Compared to our results from that year, our estimates appear slightly lower (1.1% versus 1.8% in the THIN study).[25] While NRT prescribing rates increased from 4% in 2007 to 5% in 2015 in people with dementia, these rates declined over time in people without dementia. A recent report from the British Lung Foundation found that NRT prescribing through primary care in England has dropped about 75% during the last 10 years. That was mainly due to cuts to public health funding that would have adversely impacted specialist stop smoking services.[26]

Our study is among the first to investigate longer term smoking cessation after being prescribed varenicline and NRT amongst individuals with dementia in a real world setting. Our results

For peer review only - http://bmjopen.bmj.com/site/about/guidelines.xhtml

suggest that both varenicline and NRT could produce long term smoking cessation in people with dementia as they do in those without. Almost one third of smokers with dementia quit smoking after 2 years follow-up. Regardless of the smoking cessation medication prescribed to people with dementia, it is important to acknowledge that achieving smoking cessation in this group may carry health benefits which would potentially improve their general health status and and/or extend life expectancy.[27] This should ideally be coupled with improving diet quality and increasing physical activites that may shield quitters from weight gain after smoking cessation.[28]

It is not clear why individuals with dementia are less likely to be prescribed varenicline than NRT compared to individuals without. Previous clinical and observational studies have established that varenicline is superior to single form NRT in achieving smoking cessation in different groups.[12 29 30] Additionally, varenicline did not seem to be associated with an increased risk of documented cardiovascular events, depression, or self-harm when compared with NRT in primary care in England.[31] On the other hand, a recent study based on CPRD data concluded that NRT appears to increase cardiovascular events for patients prescribed NRT, compared with those receiving smoking cessation advice after 52 weeks of follow-up.[32] This was consistent with the evidence shown by a meta-analysis of 120 studies involving 177, 390 individuals.[33] It is possible that GPs are less likely to prescribe varenicline to individuals with dementia because of lower likelihood of adherence; in a recent systematic review of the literature, older patients with dementia were found to have a low level of medication adherence.[34]

In summary, age- and sex-adjusted smoking prevalence amongst individuals with dementia was similar to those without dementia and smoking cessation rates were similar following prescription of smoking cessation medications between these groups. However, individuals with dementia were less likely to be prescribed varenicline than individuals without dementia.

For peer review only - http://bmjopen.bmj.com/site/about/guidelines.xhtml

Funding

This research was supported by Global Research Awards for Nicotine Dependence (GRAND), an independently reviewed competitive grants program supported by Pfizer, to the University of Bristol. The funder had no role in study design, data collection and analysis, decision to publish, or preparation of the manuscript.

KHT is funded by a National Institute for Health Research postdoctoral fellowship (PDF-2017-10-068). TJ receives funding from the National Institute for Health Research Collaboration for Leadership in Applied Health Research and Care (NIHR CLAHRC) West. The views expressed are those of the authors and not necessarily those of the NHS, the NIHR or the Department of Health. RMM is supported by the NIHR Bristol Biomedical Research Centre, a partnership between the University Hospitals Bristol NHS Foundation Trust and the University of Bristol; and by a Cancer Research UK Programme Grant (C18281/A19169). GT is funded by a Cancer Research UK Population Researcher Postdoctoral Fellowship Award (C56067/A21330).

Data statement

This study is based in part on data from the Clinical Practice Research Datalink obtained under licence from the UK Medicines and Healthcare Products Regulatory Agency. The data are provided by patients and collected by the NHS as part of their care and support. The interpretation and conclusions contained in this study are those of the authors alone.

Data and analytic code availability

Codelists that were used for this study are available in the supplementary file (eTables 6 and 7).

For peer review only - http://bmjopen.bmj.com/site/about/guidelines.xhtml

Contributorship Statement

TI contributed to data cleaning, data analysis, interpretation of results and writing the manuscript. RMM, GT, ND, AT and KT contributed to study conceptualization, study design, interpretation of results, data analysis and writing the manuscript. MM, and DR contributed to study conceptualization, study design, interpretation of results and writing the manuscript. TJ extracted the data and contributed to writing the manuscript. TI, AT, and ND had full access to all of the data in the study and takes responsibility for the integrity of the data and the accuracy of the data analysis.

For peer review only

Figure legends

Figure 1. Percentage (%) of primary care patients with an electronic medical record indicating smoking, from 2007 to 2015, in people with or without dementia

Figure 2. Prescription prevalence of varenicline or NRT in primary care, from 2007 to 2015, in smokers with dementia, compared to smokers without dementia

Figure 3. Percentage (%) of people with an electronic medical record indicating smoking cessation at up to 2 years follow-up, in people with and without dementia

For peer review only - http://bmjopen.bmj.com/site/about/guidelines.xhtml

References

1. Reitsma MB, Fullman N, Ng M, et al. Smoking prevalence and attributable disease burden in 195 countries and territories, 1990-2015: a systematic analysis from the Global Burden of Disease Study 2015. *The Lancet* 2017;389(10082):1885-906. doi: 10.1016/S0140-6736(17)30819-X
2. Almeida OP, Hulse GK, Lawrence D, et al. Smoking as a risk factor for Alzheimer's disease: contrasting evidence from a systematic review of case-control and cohort studies. *Addiction* 2002;97(1):15-28.
3. Anstey KJ, von Sanden C, Salim A, et al. Smoking as a risk factor for dementia and cognitive decline: a meta-analysis of prospective studies. *Am J Epidemiol* 2007;166(4):367-78. doi: 10.1093/aje/kwm116
4. McKenzie J, Bhatti L, Tursan d'Espaignet E. WHO Tobacco Knowledge Summaries: Tobacco and dementia: World Health Organization, 2014.
5. Swan GE, Lessov-Schlaggar CN. The effects of tobacco smoke and nicotine on cognition and the brain. *Neuropsychol Rev* 2007;17(3):259-73. doi: 10.1007/s11065-007-9035-9
6. Saito EK, Diaz N, Chung J, et al. Smoking history and Alzheimer's disease risk in a community-based clinic population. *Journal of Education and Health Promotion* 2017;6:24. doi: 10.4103/jehp.jehp_45_15
7. Takahashi PY, Caldwell CR, Targonski PV. Effect of alcohol and tobacco use on vascular dementia: a matched case control study. *Vascular health and risk management* 2011;7:685-91. doi: 10.2147/vhrm.s26194 [published Online First: 2011/12/06]
8. Heward M, Kelly F. Research and education to understand fire risks associated with dementia: A collaborative case study (innovative practice). *Dementia (London, England)* 2018:1471301218790850. doi: 10.1177/1471301218790850 [published Online First: 2018/08/03]
9. Cataldo JK. Clinical implications of smoking and aging: breaking through the barriers. *J Gerontol Nurs* 2007;33(8):32-41.
10. Choi D, Choi S, Park SM. Effect of smoking cessation on the risk of dementia: a longitudinal study. *Annals of Clinical and Translational Neurology* 2018;5(10):1192-99. doi: doi:10.1002/acn3.633
11. Taylor G, McNeill A, Girling A, et al. Change in mental health after smoking cessation: systematic review and meta-analysis. *BMJ* 2014;348:g1151. doi: 10.1136/bmj.g1151
12. Taylor GMJ, Taylor AE, Thomas KH, et al. The effectiveness of varenicline versus nicotine replacement therapy on long-term smoking cessation in primary care: a prospective cohort study of electronic medical records. *Int J Epidemiol* 2017;46(6):1948-57. doi: 10.1093/ije/dyx109
13. Brose LS, West R, Stapleton JA. Comparison of the effectiveness of varenicline and combination nicotine replacement therapy for smoking cessation in clinical practice. *Mayo Clin Proc* 2013;88(3):226-33. doi: 10.1016/j.mayocp.2012.11.013
14. Cahill K, Stevens S, Perera R, et al. Pharmacological interventions for smoking cessation: an overview and network meta-analysis. *Cochrane Database Syst Rev* 2013(5):CD009329. doi: 10.1002/14651858.CD009329.pub2
15. Herrett E, Gallagher AM, Bhaskaran K, et al. Data Resource Profile: Clinical Practice Research Datalink (CPRD). *Int J Epidemiol* 2015;44(3):827-36. doi: 10.1093/ije/dyv098

For peer review only - http://bmjopen.bmj.com/site/about/guidelines.xhtml

16. Herrett E, Thomas SL, Schoonen WM, et al. Validation and validity of diagnoses in the General Practice Research Database: a systematic review. *Br J Clin Pharmacol* 2010;69(1):4-14. doi: 10.1111/j.1365-2125.2009.03537.x
17. Khan NF, Harrison SE, Rose PW. Validity of diagnostic coding within the General Practice Research Database: a systematic review. *Br J Gen Pract* 2010;60(572):e128-36. doi: 10.3399/bjgp10X483562
18. Hernan MA, Alonso A, Logan R, et al. Observational studies analyzed like randomized experiments: an application to postmenopausal hormone therapy and coronary heart disease. *Epidemiology* 2008;19(6):766-79. doi: 10.1097/EDE.0b013e3181875e61
19. West R, Hajek P, Stead L, et al. Outcome criteria in smoking cessation trials: proposal for a common standard. *Addiction* 2005;100(3):299-303. doi: 10.1111/j.1360-0443.2004.00995.x
20. Khan NF, Perera R, Harper S, et al. Adaptation and validation of the Charlson Index for Read/OXMIS coded databases. *BMC Fam Pract* 2010;11:1. doi: 10.1186/1471-2296-11-1
21. Sterne JA, White IR, Carlin JB, et al. Multiple imputation for missing data in epidemiological and clinical research: potential and pitfalls. *BMJ* 2009;338:b2393. doi: 10.1136/bmj.b2393
22. Deng J, Cao C, Jiang Y, et al. Prevalence and effect factors of dementia among the community elderly in Chongqing, China. *Psychogeriatrics : the official journal of the Japanese Psychogeriatric Society* 2018;18(5):412-20. doi: 10.1111/psyg.12343 [published Online First: 2018/05/16]
23. Nakahori N, Sekine M, Yamada M, et al. A pathway from low socioeconomic status to dementia in Japan: results from the Toyama dementia survey. *BMC Geriatr* 2018;18(1):102. doi: 10.1186/s12877-018-0791-6
24. Smoking in England (2017). Latest trends on smoking in England from the Smoking Toolkit Study. Available at: http://www.smokinginengland.info/sts-documents/
25. Huang Y, Lewis S, Britton J. Use of varenicline for smoking cessation treatment in UK primary care: an association rule mining analysis. *BMC Public Health* 2014;14:1024. doi: 10.1186/1471-2458-14-102426. British Lung Foundation (2018). Available at: https://cdn.shopify.com/s/files/1/0221/4446/files/Tobacco_Report_July_2018_PDF.pdf?5789129449046780869&_ga=2.142198583.422598499.1547211481-1798507517.1544436747
27. Jha P, Ramasundarahettige C, Landsman V, et al. 21st-century hazards of smoking and benefits of cessation in the United States. *N Engl J Med* 2013;368(4):341-50. doi: 10.1056/NEJMsa1211128
28. Hu Y, Zong G, Liu G, et al. Smoking Cessation, Weight Change, Type 2 Diabetes, and Mortality. *N Engl J Med* 2018;379(7):623-32. doi: 10.1056/NEJMoa1803626
29. Anthenelli RM, Benowitz NL, West R, et al. Neuropsychiatric safety and efficacy of varenicline, bupropion, and nicotine patch in smokers with and without psychiatric disorders (EAGLES): a double-blind, randomised, placebo-controlled clinical trial. *Lancet* 2016;387(10037):2507-20. doi: 10.1016/S0140-6736(16)30272-0
30. Chang PY, Shiu MN, Yuan YT, et al. Comparative effectiveness of varenicline and nicotine replacement therapy for smoking cessation in older and younger smokers: a prospective cohort in Taiwan. *Nicotine Tob Res* 2017 doi: 10.1093/ntr/ntx275

For peer review only

For peer review only - http://bmjopen.bmj.com/site/about/guidelines.xhtml

31. Kotz D, Viechtbauer W, Simpson C, et al. Cardiovascular and neuropsychiatric risks of varenicline: a retrospective cohort study. *Lancet Respir Med* 2015;3(10):761-8. doi: 10.1016/S2213-2600(15)00320-3
32. Dollerup J, Vestbo J, Murray-Thomas T, et al. Cardiovascular risks in smokers treated with nicotine replacement therapy: a historical cohort study. *Clinical Epidemiology* 2017;9:231-43. doi: 10.2147/CLEP.S127775
33. Hartmann-Boyce J, Chepkin SC, et al. Nicotine replacement therapy versus control for smoking cessation.*Cochrane Database of Systematic Reviews* 2018. doi: 10.1002/14651858.CD000146.pub5
34. El-Saifi N, Moyle W, Jones C, et al. Medication Adherence in Older Patients With Dementia: A Systematic Literature Review. *J Pharm Pract* 2018;31(3):322-34. doi: 10.1177/0897190017710524

For peer review only

For peer review only - http://bmjopen.bmj.com/site/about/guidelines.xhtml

Table 1. Baseline characteristics of people with or without dementia by exposure group, N (%)

Characteristic	People with dementia (N=447)			People without dementia (N=234,867)		
	NRT (N=409)	Varenicline (N=38)	Total	NRT (N=159,327)	Varenicline (N=75,540)	Total
Age at time of first prescription[1]	71.1 (12.2)	66.2 (15.1)	70.7 (12.6)	46.2 (15.5)	44.4 (13.2)	45.6 (14.8)
Sex (male)	186 (45.5%)	19 (50.0%)	205 (45.9%)	73,674 (46.2%)	37,676 (49.9%)	111,350 (47.4%)
Index of multiple deprivation score (IMD)[2†]	3	4	3	3	3	3
Number of GP visits 1-year prior to first prescription[1]	11.5 (9.0)	15.3 (9.9)	11.8 (9.1)	8.9 (7.4)	7.3 (6.1)	8.4 (7.0)
BMI[1†]	24.6 (5.1)	25.7 (6.3)	24.7 (5.4)	26.5 (5.7)	26.5 (5.4)	26.5 (5.6)
Year of first prescription[2]	2010	2010	2010	2009	2010	2009
Days of history[1]	3,573.8 (2181.2)	3,450.3 (2327.4)	3,563.3 (2191.5)	3,052.9 (1907.1)	3,164.8 (1986.2)	3,088.9 (1933.6)
Comorbidity ever (Charlson Index)	354 (86.6%)	28 (73.7%)	382 (85.5%)	59,489 (37.3%)	24,017 (31.8%)	83,506 (35.6%)
Alcohol misuse ever	104 (25.4%)	11 (29.0%)	115 (25.7%)	13,890 (8.7%)	4,759 (6.3%)	18,649 (7.9%)
Self-harm ever	67 (16.4%)	9 (23.7%)	76 (17.0%)	17,232 (10.8)	6,652 (8.8%)	23,884 (10.2%)
Ever anxiety and stress related disorders	151 (36.9%)	16 (42.1%)	167 (37.4%)	44,381 (27.9%)	17,377 (23.0%)	61,758 (26.3%)
Other behavioural/neurologic disorder ever	30 (7.3%)	6 (15.8%)	36 (8.1%)	8,693 (5.5%)	2,956 (3.9%)	11,649 (5.0%)
Ever depression	217 (53.1%)	26 (68.4%)	243 (54.4%)	65,343 (41.0%)	26,097 (34.6%)	91,440 (38.9%)
Ever antidepressants	273 (66.7%)	28 (73.7%)	301 (67.3%)	79.584 (50.0%)	32,230 (42.7%)	111,814 (47.6%)
Ever antipsychotics	175 (42.8%)	13 (34.2%)	188 (42.1%)	28,972 (18.2%)	9,792 (13.0%)	38,764 (16.5%)
Ever hypnotics/anxiolytics	238 (58.2%)	20 (52.6%)	258 (57.7%)	60,092 (37.7%)	25,134 (33.3%)	85,226 (36.3%)

1 Data presented are mean and standard deviation. 2 Data presented are median. †Missing data: BMI data was missing for 14.1% (N= 33,059); IMD data was missing for 43.6% (N= 102,657).

For peer review only - http://bmjopen.bmj.com/site/about/guidelines.xhtml

Figure 1. Percentage (%) of primary care patients with an electronic medical record indicating smoking, from 2007 to 2015, in people with or without dementia

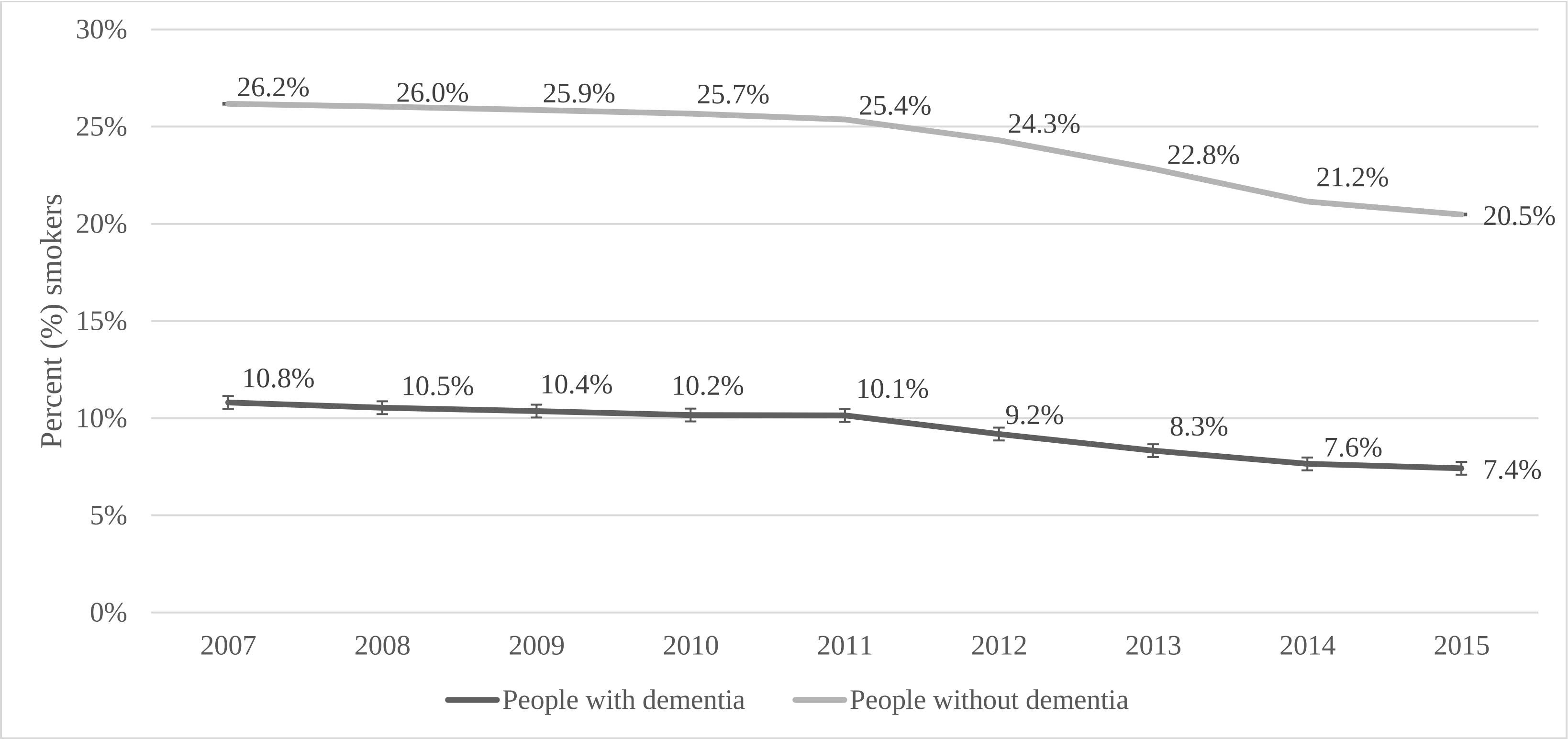

Figure 2. Prescription prevalence of varenicline or NRT in primary care, from 2007 to 2015, in smokers with dementia, compared to smokers without dementia

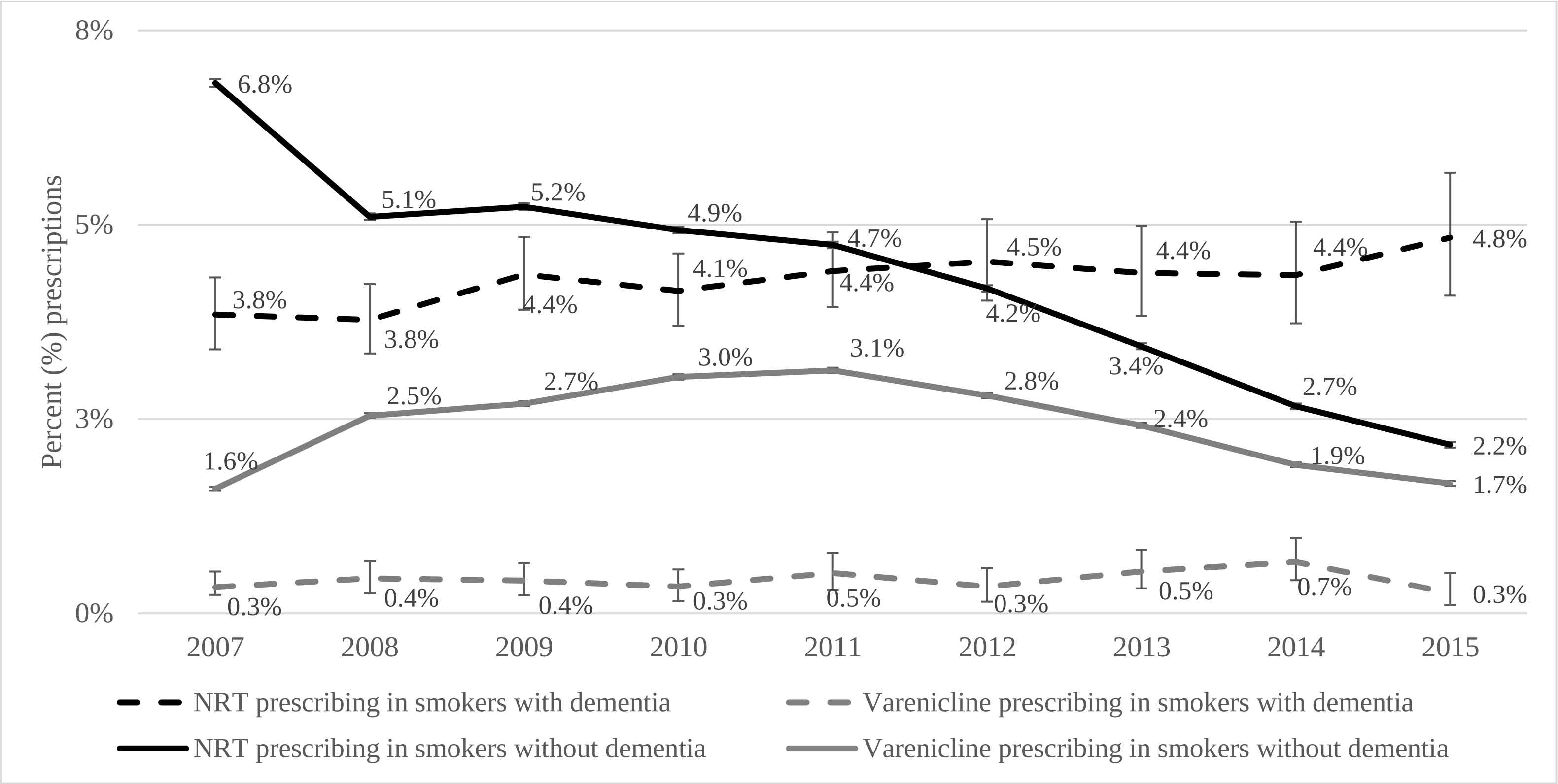

Figure 3. Percentage (%) of people with an electronic medical record indicating smoking cessation at up to 2-years follow-up, in people with and without dementia

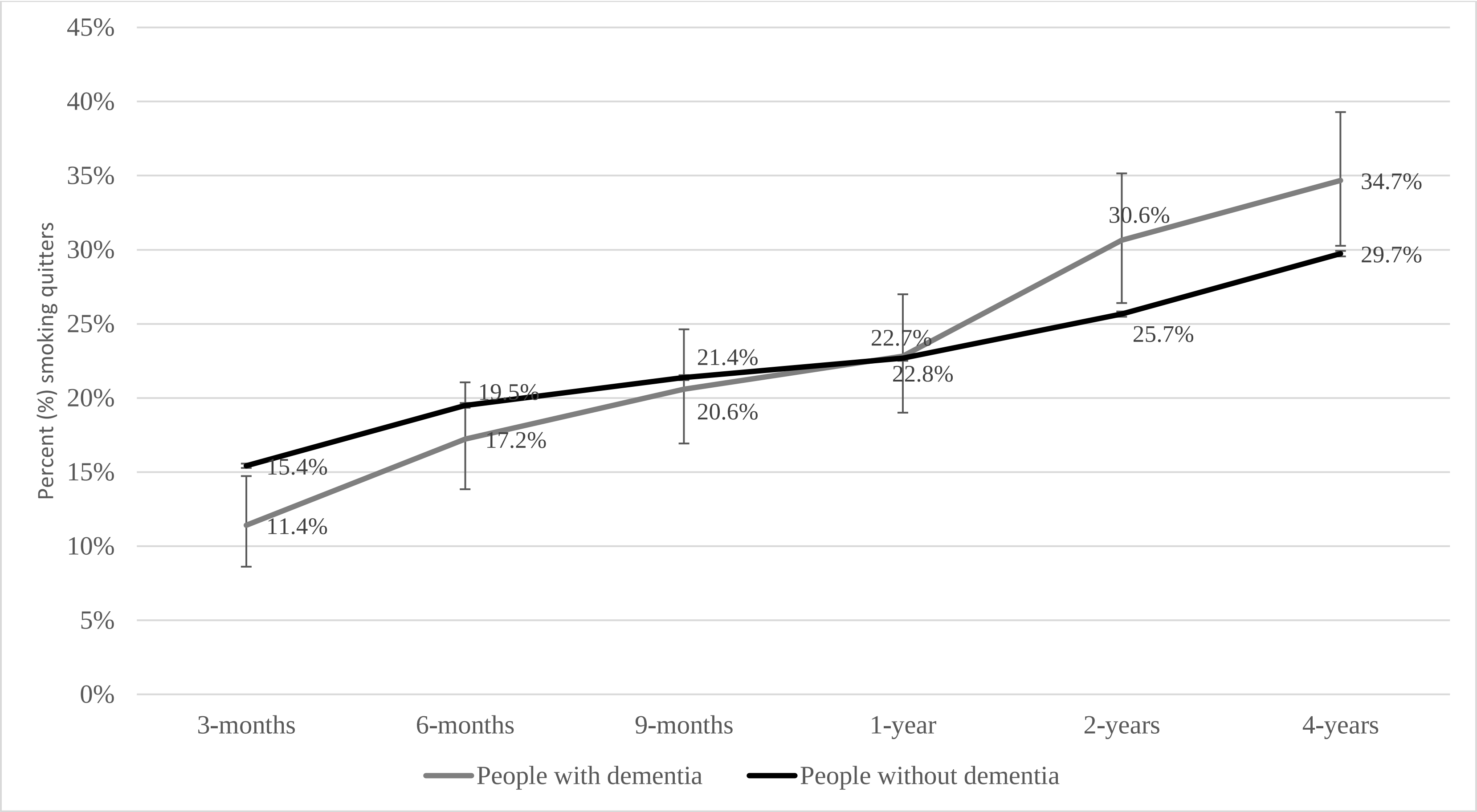

eFigure 1. Flow chart of eligible study participants as per protocol restrictions

Assessed for eligibility
N Records=2,877,296
N Patients=566,936

Excluded from analysis due to protocol restrictions (Davies et al. 2015)

	Prescriptions	Patients
Prescriptions issued to patients under the age of 15	6580	1944
Prescription issued after patients' registration period ended	0	0
Prescription issued before patients' registration period began	296490	53529
Prescription issued to patient aged 16 or 17	13154	4425
Prescription issued before 1st September 2006	822244	145382
Both varenicline and NRT were prescribed on the same day	8289	264
Smoking cessation medication was not prescribed by a general practitioner (GP)	141810	16168
Smoking cessation medication prescription had less than one year of historical follow-up data prior to prescription	163821	41112
Bupropion prescriptions	32997	6981
Prescriptions issued to patients who received a smoking cessation medication issued in the previous 18 months	1069188	30278
Patient had previously received an eligible smoking cessation medication prescription	55870	0
Prescribing GP had seen less than 10 patients.	18494	18494
Patients had less than 180 days of follow-up after 1st prescription.	13045	13045
Total excluded N=	2,641,982	331,622

Included in analysis
N patients=235,314

People with dementia
N patients=447

People without dementia
N patients=234,867

For peer review only

eFigure 2. Age and sex standardised percentage (%) of primary care patients with an electronic medical record indicating smoking, from 2007 to 2015, in people with or without dementia

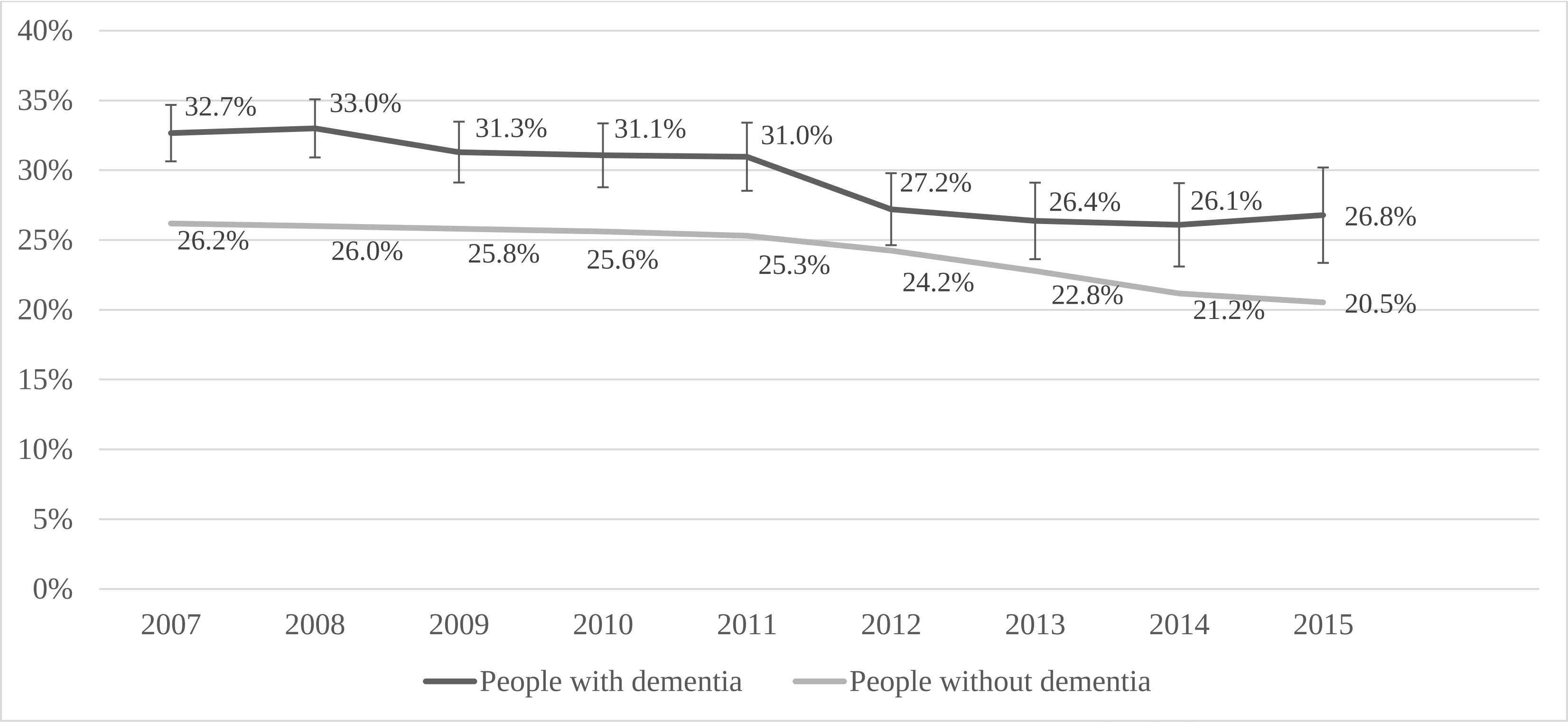

Note on age and sex standardisation

Smoking prevalence rates were directly age- and sex- standardised to account for differences in age and sex between groups with and without dementia. We used the CPRD population in 2015 as our standard population for calculating standardised smoking rates. Age was grouped into three categories (18-49, 50-59, 60+ years).

eTable 1. Distributions of imputed characteristics in the imputation datasets

Characteristic	People with dementia (N=447)							People without dementia (N=234,867)						
	NRT (N=409)		Varenicline (N=38)		Total		% of data imputed	NRT (N=159,327)		Varenicline (N=75,540)		Total		% of data imputed
	Mean	Standard error	Mean	Standard error	Mean	Standard error		Mean	Standard error	Mean	Standard error	Mean	Standard error	
Body mass index	24.6	0.28	26.6	1.02	24.7	0.27	10.3	26.4	0.02	26.5	0.02	26.4	0.01	14.1
Index of multiple deprivation	3.3	0.08	3.3	0.3	3.3	0.09	53.2	3.3	0.004	3.2	0.007	3.3	0.003	43.6

For peer review only - http://bmjopen.bmj.com/site/about/guidelines.xhtml

eTable 2. Raw numbers for smoking prevalence calculations amongst people with and without dementia, 2007-2015

**Year**	2007	2008	2009	2010	2011	2012	2013	2014	2015
People with dementia									
Numerator (number of smokers with dementia)	2,965	3,126	3,326	3,495	3,678	3,513	3,335	3,033	2,690
Denominator (number people with dementia)	27,432	29664	32,090	34,391	36,284	38,251	40,043	39,665	36,249
People without dementia									
Numerator (number of smokers without dementia)	1,010,530	1,013,985	1,018,065	1,015,538	987,193	930,783	866,310	748,084	628,444
Denominator (number of people without dementia)	3,860,169	3,895,542	3,936,638	3,955,107	3,892,231	3,830,563	3,795,481	3,536,957	3,068,743

For peer review only

eTable 3. The likelihood of smokers with ever dementia diagnosis being prescribed varenicline versus NRT, as compared to smokers with no ever dementia diagnosis, N=235,314

	Partially adjusted odds ratio (95% confidence interval) †	Fully adjusted odds ratio (95% confidence interval) ††
Ever dementia diagnosis	0.21 (0.15 to 0.29)	0.26 (0.18 to 0.36)

‡Partially adjusted models were adjusted for: age, sex, year of first prescription.
†† Fully adjusted models were adjusted for: age, sex, days in history, IMD, number of GP visits 1-year prior to first prescription, BMI, year of first prescription, history of major physical morbidity (Charlson Index), alcohol misuse ever, drug misuse ever, depression ever, neurotic disorder ever, self-harm ever, antidepressant prescription ever, antipsychotic prescription ever, hypnotics/anxiolytics prescription ever, other psychotropic medication ever, and other behavioral/neurologic disorder ever. Missing BMI and IMD values were imputed using multiple imputation.
Models were estimated using cluster robust standard errors to account for potential clustering of patients between practices.

For peer review only

For peer review only - http://bmjopen.bmj.com/site/about/guidelines.xhtml

eTable 4. Number and percentage (%) of people with an electronic medical record indicating smoking cessation at 3, 6 and 9-months, and 1, 2, and 4-years follow-up, in people with and without dementia

	3-months	6-months	9-months	1-year	2-years	4-years
People without dementia N=234867	36223 (15.4%)	45796 (19.5%)	50193 (21.4%)	53263 (22.7%)	60257 (25.7%)	69842 (29.7%)
People with dementia N=447	51 (11.4%)	77 (17.2%)	92 (20.6%)	102 (22.8%)	137 (30.6%)	155 (34.7%)

For peer review only

For peer review only - http://bmjopen.bmj.com/site/about/guidelines.xhtml

eTable 5. Fully adjusted odds ratios and 95% confidence intervals for the association between the diagnosis of dementia and smoking cessation at 3, 6 and 9-months and 1, 2, and 4-years after prescription

Fully adjusted odds ratio (95% confidence interval) †						
	3-months	6-months	9-months	1-year	2-years	4-years
(N=235,314)	0.56 (0.41 to 0.75)	0.64 (0.49 to 0.83)	0.71 (0.56 to 0.90)	0.75 (0.60 to 0.94)	1.0 (0.81 to 1.23)	1.04 (0.85 to 1.26)

† Fully adjusted models were adjusted for: type of prescription, age, sex, days in history, IMD, number of GP visits 1-year prior to first prescription, BMI, year of first prescription, history of major physical morbidity (Charlson Index), alcohol misuse ever, drug misuse ever, depression ever, neurotic disorder ever, self-harm ever, antidepressant prescription ever, antipsychotic prescription ever, hypnotics/anxiolytics prescription ever, other psychotropic medication ever, and other behavioral/neurologic disorder ever. Missing BMI and IMD values were imputed using multiple imputation.

For peer review only

eTable 6. List of dementia medications codes used in study cohort

Prodcode	Product name
39363	Ebixa 20mg tablets (Lundbeck Ltd)
58937	Exelon 13.3mg/24hours transdermal patches (Novartis Pharmaceuticals UK Ltd)
6225	Memantine 10mg tablets
11751	Rivastigmine 3mg capsules
7329	Galantamine 20mg/5ml oral solution sugar free
60723	Rivastigmine 6mg capsules (Waymade Healthcare Plc)
58780	Voleze 9.5mg/24hours transdermal patches (Focus Pharmaceuticals Ltd)
39362	Ebixa tablets treatment initiation pack (Lundbeck Ltd)
56631	Rivastigmine 13.3mg/24hours transdermal patches
37132	Rivastigmine 9.5mg/24hours transdermal patches
56771	Rivastigmine 3mg capsules (Dr Reddy's Laboratories (UK) Ltd)
20404	Exelon 4.5mg capsules (Novartis Pharmaceuticals UK Ltd)
57171	Erastig 9.5mg/24hours transdermal patches (Teva UK Ltd)
61676	Donepezil 1mg/ml oral solution sugar free
24088	Reminyl XL 24mg capsules (Shire Pharmaceuticals Ltd)
11635	Galantamine 12mg tablets
60192	Galantex XL 8mg capsules (Creo Pharma Ltd)
57627	Erastig 4.6mg/24hours transdermal patches (Teva UK Ltd)
11654	Galantamine 8mg tablets
2930	Donepezil 5mg tablets
5616	Exelon 6mg capsules (Novartis Pharmaceuticals UK Ltd)
58969	Rivastigmine 4.6mg/24hours transdermal patches (A A H Pharmaceuticals Ltd)
48482	Galsya XL 8mg capsules (Consilient Health Ltd)
48442	Donepezil 5mg orodispersible tablets
55928	Exelon 4.5mg capsules (Waymade Healthcare Plc)
53882	Rivastigmine 2mg/ml oral solution
58709	Donepezil 10mg tablets (A A H Pharmaceuticals Ltd)
59871	Donepezil 10mg/5ml oral suspension

For peer review only

55720	Gatalin XL 24mg capsules (Aspire Pharma Ltd)
7361	Galantamine 24mg modified-release capsules
61476	Acumor XL 24mg capsules (Generics (UK) Ltd)
57139	Ebixa 10mg tablets (DE Pharmaceuticals)
62164	Alzest 9.5mg/24hours transdermal patches (Dr Reddy's Laboratories (UK) Ltd)
53922	Donepezil 10mg orodispersible tablets (Consilient Health Ltd)
36848	Aricept Evess 5mg orodispersible tablets (Eisai Ltd)
60493	Galantex XL 24mg capsules (Creo Pharma Ltd)
29288	Reminyl 4mg/ml oral solution (Shire Pharmaceuticals Ltd)
9966	Ebixa 5mg/pump actuation oral solution (Lundbeck Ltd)
58947	Donepezil 10mg tablets (Accord Healthcare Ltd)
5247	Aricept 10mg tablets (Eisai Ltd)
11716	Exelon 3mg capsules (Novartis Pharmaceuticals UK Ltd)
61920	Luventa XL 8mg capsules (Fontus Health Ltd)
10187	Galantamine 4mg tablets
37444	Exelon 4.6mg/24hours transdermal patches (Novartis Pharmaceuticals UK Ltd)
18587	Reminyl XL 8mg capsules (Shire Pharmaceuticals Ltd)
56421	Gatalin XL 8mg capsules (Aspire Pharma Ltd)
10255	Galantamine 8mg modified-release capsules
4597	Rivastigmine 1.5mg capsules
9854	Reminyl 4mg tablets (Shire Pharmaceuticals Ltd)
61385	Nemdatine 10mg tablets (Actavis UK Ltd)
11546	Exelon 1.5mg capsules (Novartis Pharmaceuticals UK Ltd)
14309	Galantamine 16mg modified-release capsules
37188	Aricept Evess 10mg orodispersible tablets (Eisai Ltd)
5334	Reminyl 12mg tablets (Shire Pharmaceuticals Ltd)
56709	Gatalin XL 16mg capsules (Aspire Pharma Ltd)
2931	Donepezil 10mg tablets
61921	Luventa XL 24mg capsules (Fontus Health Ltd)
11827	Rivastigmine 2mg/ml oral solution sugar free
37957	Exelon 9.5mg/24hours transdermal patches (Novartis Pharmaceuticals UK Ltd)

For peer review only - http://bmjopen.bmj.com/site/about/guidelines.xhtml

48015	Galsya XL 24mg capsules (Consilient Health Ltd)
53842	Aricept 5mg tablets (Waymade Healthcare Plc)
18800	Ebixa 10mg tablets (Lundbeck Ltd)
59993	Galantex XL 16mg capsules (Creo Pharma Ltd)
36976	Rivastigmine 4.6mg/24hours transdermal patches
56600	Donepezil 5mg tablets (Zentiva)
35088	Donepezil 10mg orodispersible tablets sugar free
5400	Aricept 5mg tablets (Eisai Ltd)
59330	Voleze 4.6mg/24hours transdermal patches (Focus Pharmaceuticals Ltd)
18062	Reminyl 8mg tablets (Shire Pharmaceuticals Ltd)
11752	Rivastigmine 4.5mg capsules
38976	Memantine 5mg+10mg+15mg+20mg Tablet
35179	Donepezil 5mg orodispersible tablets sugar free
60107	Donepezil 5mg tablets (Alliance Healthcare (Distribution) Ltd)
20140	Reminyl XL 16mg capsules (Shire Pharmaceuticals Ltd)
39240	Memantine 20mg tablets
18556	Exelon 2mg/ml oral solution (Novartis Pharmaceuticals UK Ltd)
9786	Rivastigmine 6mg capsules
11837	Memantine 10mg/ml oral solution sugar free
48443	Donepezil 10mg orodispersible tablets
61618	Nemdatine 20mg tablets (Actavis UK Ltd)
12843	Ginkyo 120mg tablets (Ceuta Healthcare Ltd)
30120	Ginkyo 50mg tablets (Ceuta Healthcare Ltd)
61128	HealthAid Ginko Vital (Biloba) 5g capsules (HealthAid Ltd)

For peer review only - http://bmjopen.bmj.com/site/about/guidelines.xhtml

eTable 7. List of dementia diagnoses codes used in study cohort

medcode	readcode	readterm
26270	Eu02500	[X]Lewy body dementia
44674	E002.00	Senile dementia with depressive or paranoid features
19393	Eu01z00	[X]Vascular dementia, unspecified
4693	Eu02z00	[X] Unspecified dementia
25704	Eu00011	[X]Presenile dementia,Alzheimer's type
19477	E004.00	Arteriosclerotic dementia
55313	Eu01y00	[X]Other vascular dementia
30032	E001200	Presenile dementia with paranoia
18386	E002000	Senile dementia with paranoia
56912	E004100	Arteriosclerotic dementia with delirium
33707	E00..00	Senile and presenile organic psychotic conditions
4357	Eu02z14	[X] Senile dementia NOS
12710	6AB..00	Dementia annual review
41089	E002z00	Senile dementia with depressive or paranoid features NOS
9509	Eu02300	[X]Dementia in Parkinson's disease
15165	E001.00	Presenile dementia
49513	E001100	Presenile dementia with delirium
42602	E001000	Uncomplicated presenile dementia
7572	F116.00	Lewy body disease
30706	Eu00200	[X]Dementia in Alzheimer's dis, atypical or mixed type
1916	E00..11	Senile dementia
55467	E004200	Arteriosclerotic dementia with paranoia
9565	Eu01.11	[X]Arteriosclerotic dementia
8934	Eu01200	[X]Subcortical vascular dementia
31016	Eu01300	[X]Mixed cortical and subcortical vascular dementia
43089	E004000	Uncomplicated arteriosclerotic dementia
11175	Eu01100	[X]Multi-infarct dementia
42279	E004z00	Arteriosclerotic dementia NOS
29386	Eu00z00	[X]Dementia in Alzheimer's disease, unspecified
38438	E001z00	Presenile dementia NOS
8634	E004.11	Multi infarct dementia
1917	F110.00	Alzheimer's disease
61528	Eu00013	[X]Alzheimer's disease type 2
1350	E00..12	Senile/presenile dementia
38678	Eu00100	[X]Dementia in Alzheimer's disease with late onset
27677	E001300	Presenile dementia with depression
46762	Eu00111	[X]Alzheimer's disease type 1
11379	Eu00112	[X]Senile dementia,Alzheimer's type
21887	E002100	Senile dementia with depression
2882	E00z.00	Senile or presenile psychoses NOS
25386	E041.00	Dementia in conditions EC
49263	Eu00000	[X]Dementia in Alzheimer's disease with early onset
59122	Fyu3000	[X]Other Alzheimer's disease
34944	Eu02z13	[X] Primary degenerative dementia NOS

For peer review only - http://bmjopen.bmj.com/site/about/guidelines.xhtml

40805	9hD1.00	Excepted from dementia quality indicators: Informed dissent
37015	E003.00	Senile dementia with delirium
6578	Eu01.00	[X]Vascular dementia
7323	E000.00	Uncomplicated senile dementia
55838	Eu01111	[X]Predominantly cortical dementia
29512	F112.00	Senile degeneration of brain
12621	Eu02.00	[X]Dementia in other diseases classified elsewhere
7664	Eu00.00	[X]Dementia in Alzheimer's disease
60059	Eu00012	[X]Primary degen dementia, Alzheimer's type, presenile onset
15249	E00y.00	Other senile and presenile organic psychoses
43346	Eu00113	[X]Primary degen dementia of Alzheimer's type, senile onset
53446	Eu04100	[X]Delirium superimposed on dementia
43292	E004300	Arteriosclerotic dementia with depression
32057	F110100	Alzheimer's disease with late onset
8195	Eu00z11	[X]Alzheimer's dementia unspec
64267	Eu02y00	[X]Dementia in other specified diseases classif elsewhere
46488	Eu01000	[X]Vascular dementia of acute onset
27759	Eu02z16	[X] Senile dementia, depressed or paranoid type
16797	F110000	Alzheimer's disease with early onset
48501	Eu02z11	[X] Presenile dementia NOS
109047	8BPa.00	Antipsychotic drug therapy for dementia
106311	8CMZ.00	Dementia care plan
103445	8Hla.00	Referral to dementia care advisor
44341	9hD..00	Exception reporting: dementia quality indicators
28402	Eu02000	[X]Dementia in Pick's disease
54106	Eu02100	[X]Dementia in Creutzfeldt-Jakob disease
37014	Eu02200	[X]Dementia in Huntington's disease
41185	Eu02400	[X]Dementia in human immunodef virus [HIV] disease
5931	1461	H/O: dementia
11136	F111.00	Pick's disease
62132	E02y100	Drug-induced dementia
2731	F11z.11	Cerebral atrophy
54744	F11x200	Cerebral degeneration due to cerebrovascular disease

For peer review only

For peer review only - http://bmjopen.bmj.com/site/about/guidelines.xhtml

STROBE Statement—Checklist of items that should be included in reports of ***cohort studies***

	Item No	Recommendation	Page
**Title and abstract**	1	(*a*) Indicate the study's design with a commonly used term in the title or the abstract	P3L43
		(*b*) Provide in the abstract an informative and balanced summary of what was done and what was found	P4L69
**Introduction**			
Background/rationale	2	Explain the scientific background and rationale for the investigation being reported	P5L92
Objectives	3	State specific objectives, including any prespecified hypotheses	P6L112
**Methods**			
Study design	4	Present key elements of study design early in the paper	P7L120
Setting	5	Describe the setting, locations, and relevant dates, including periods of recruitment, exposure, follow-up, and data collection	P7L136
Participants	6	(*a*) Give the eligibility criteria, and the sources and methods of selection of participants. Describe methods of follow-up	P7L136
		(*b*) For matched studies, give matching criteria and number of exposed and unexposed	NA
Variables	7	Clearly define all outcomes, exposures, predictors, potential confounders, and effect modifiers. Give diagnostic criteria, if applicable	P8-10L154-185
Data sources/ measurement	8*	For each variable of interest, give sources of data and details of methods of assessment (measurement). Describe comparability of assessment methods if there is more than one group	P7-8L136-152
Bias	9	Describe any efforts to address potential sources of bias	P11L192-195
Study size	10	Explain how the study size was arrived at	Supplementary eFigure 1
Quantitative variables	11	Explain how quantitative variables were handled in the analyses. If applicable, describe which groupings were chosen and why	P11L201-P12L218
Statistical methods	12	(*a*) Describe all statistical methods, including those used to control for confounding	P11L201-P12L218
		(*b*) Describe any methods used to examine subgroups and interactions	NA
		(*c*) Explain how missing data were addressed	P10L181-183, P11L192-195
		(*d*) If applicable, explain how loss to follow-up was addressed	NA
		(*e*) Describe any sensitivity analyses	NA
**Results**			
Participants	13*	(a) Report numbers of individuals at each stage of study—eg numbers potentially eligible, examined for eligibility, confirmed eligible, included in the study, completing follow-up, and analysed	Supplementary eFigure 1
		(b) Give reasons for non-participation at each stage	Supplementary eFigure 1
		(c) Consider use of a flow diagram	Supplementary eFigure 1
Descriptive data	14*	(a) Give characteristics of study participants (eg demographic,	Table 1

For peer review only - http://bmjopen.bmj.com/site/about/guidelines.xhtml

		clinical, social) and information on exposures and potential confounders	
		(b) Indicate number of participants with missing data for each variable of interest	Table 1
		(c) Summarise follow-up time (eg, average and total amount)	Table 1
Outcome data	15*	Report numbers of outcome events or summary measures over time	Supplementary eTables 2 and 4
Main results	16	(*a*) Give unadjusted estimates and, if applicable, confounder-adjusted estimates and their precision (eg, 95% confidence interval). Make clear which confounders were adjusted for and why they were included	Supplementary eTables 3 and 5
		(*b*) Report category boundaries when continuous variables were categorized	NA
		(*c*) If relevant, consider translating estimates of relative risk into absolute risk for a meaningful time period	NA
Other analyses	17	Report other analyses done—eg analyses of subgroups and interactions, and sensitivity analyses	NA
**Discussion**			
Key results	18	Summarise key results with reference to study objectives	P17L319-322
Limitations	19	Discuss limitations of the study, taking into account sources of potential bias or imprecision. Discuss both direction and magnitude of any potential bias	P15L264-276
Interpretation	20	Give a cautious overall interpretation of results considering objectives, limitations, multiplicity of analyses, results from similar studies, and other relevant evidence	P17L319-322
Generalisability	21	Discuss the generalisability (external validity) of the study results	P16L284-287
**Other information**			
Funding	22	Give the source of funding and the role of the funders for the present study and, if applicable, for the original study on which the present article is based	P18L324-335

*Give information separately for exposed and unexposed groups.

Note: An Explanation and Elaboration article discusses each checklist item and gives methodological background and published examples of transparent reporting. The STROBE checklist is best used in conjunction with this article (freely available on the Web sites of PLoS Medicine at http://www.plosmedicine.org/, Annals of Internal Medicine at http://www.annals.org/, and Epidemiology at http://www.epidem.com/). Information on the STROBE Initiative is available at http://www.strobe-statement.org.

For peer review only
